# Differential Colonization and Succession of Microbial Communities in Rock and Soil Substrates on a Maritime Antarctic Glacier Forefield

**DOI:** 10.3389/fmicb.2020.00126

**Published:** 2020-02-07

**Authors:** Isaac Garrido-Benavent, Sergio Pérez-Ortega, Jorge Durán, Carmen Ascaso, Stephen B. Pointing, Ricardo Rodríguez-Cielos, Francisco Navarro, Asunción de los Ríos

**Affiliations:** ^1^Departamento de Biogeoquímica y Ecología Microbiana, Museo Nacional de Ciencias Naturales, CSIC, Madrid, Spain; ^2^Departamento de Micología, Real Jardín Botánico, CSIC, Madrid, Spain; ^3^Centre for Functional Ecology, Department of Life Sciences, University of Coimbra, Coimbra, Portugal; ^4^Yale-NUS College, National University of Singapore, Singapore, Singapore; ^5^Department of Biological Sciences, National University of Singapore, Singapore, Singapore; ^6^ETSI de Telecomunicación, Departamento de Señales, Sistemas y Radiocomunicaciones, Universidad Politécnica de Madrid, Madrid, Spain; ^7^ETSI de Telecomunicación, Departamento de Matemática Aplicada a las TIC, Universidad Politécnica de Madrid, Madrid, Spain

**Keywords:** Antarctica, Livingston Island, algae, bacteria, fungi, geomicrobiology, chronosequence, primary succession

## Abstract

Glacier forefields provide a unique chronosequence to assess microbial or plant colonization and ecological succession on previously uncolonized substrates. Patterns of microbial succession in soils of alpine and subpolar glacier forefields are well documented but those affecting high polar systems, including moraine rocks, remain largely unexplored. In this study, we examine succession patterns in pioneering bacterial, fungal and algal communities developing on moraine rocks and soil at the Hurd Glacier forefield (Livingston Island, Antarctica). Over time, changes were produced in the microbial community structure of rocks and soils (ice-free for different lengths of time), which differed between both substrates across the entire chronosequence, especially for bacteria and fungi. In addition, fungal and bacterial communities showed more compositional consistency in soils than rocks, suggesting community assembly in each niche could be controlled by processes operating at different temporal and spatial scales. Microscopy revealed a patchy distribution of epilithic and endolithic lithobionts, and increasing endolithic colonization and microbial community complexity along the chronosequence. We conclude that, within relatively short time intervals, primary succession processes at polar latitudes involve significant and distinct changes in edaphic and lithic microbial communities associated with soil development and cryptogamic colonization.

## Introduction

Glacier forefields are among the most appealing settings for investigating changes in microbial community composition and structure over time (Chapin et al., 1994; Nemergut et al., 2007; Zumsteg et al., 2012; Brown and Jumpponen, 2014; Jiang et al., 2018). The establishment in these areas of chronosequences (i.e., a set of spatially arranged sites representing a temporal sequence of colonization) makes possible the characterization of primary succession and pedogenic processes (Matthews, 1992; Sigler et al., 2002; Walker et al., 2010). Hence, the study of chronosequences has provided insights into the theory of ecological succession. Studies in subpolar and alpine regions have suggested that microbial succession in soils after glacial retreat and the associated biogeochemical transformations pave the way for the colonization of vascular plants (Zumsteg et al., 2012; Brown and Jumpponen, 2014; Fernández-Martínez et al., 2017). In contrast, there is a limited knowledge on the diversity, dynamics and functional role of microbial communities in remote high polar ecosystems, such as Antarctica, where the role of plants in late successional stages is replaced with that of cryptogams, i.e., lichens and bryophytes (Sancho and Valladares, 1993; Øvstedal and Smith, 2001; Peat et al., 2007; Ochyra et al., 2008; Favero-Longo et al., 2012).

High-throughput DNA sequencing technologies allow diversity and community assembly patterns for various microbial groups to be examined during ecological succession. In Antarctica, these techniques have been applied during the last decade to describe the taxonomic and functional diversity of bacterial and eukaryotic (fungal, and rarely algal) communities inhabiting particular habitats, such as soils (e.g., Wang et al., 2015; Czechowski et al., 2016; Newsham et al., 2016; Baeza et al., 2017), rocks (Wei et al., 2016; Archer et al., 2017), ice-covered lakes (Kwon et al., 2017), and cryoconite holes (Sommers et al., 2017). Studies of Newsham et al. (2016) and Sommers et al. (2017) further explored variations in these communities along climatic and biogeochemical gradients. Compared to Arctic and high mountainous regions, where changes in microbial diversity and community differentiation in glacier forefield soil chronosequences have been extensively documented (Schütte et al., 2010; Brown and Jumpponen, 2014; Kazemi et al., 2016; Fernández-Martínez et al., 2017; Jiang et al., 2018), only few studies have investigated microbial diversity dynamics during ecological succession processes in Antarctic deglaciated areas, and these have focused mostly on bacteria (Pessi et al., 2012, 2015; Bajerski and Wagner, 2013; Yan et al., 2017; Kim et al., 2019). These works suggested that glacier forefields at these latitudes were hotspots for soil bacterial diversity, with high metabolic potentials, and highlighted the differential contribution of abundant and rare bacteria, as well as particular soil attributes and spatial heterogeneity, to community shifts. However, to the best of our knowledge, there is no study exploring microbial community assembly and dynamics in Antarctica that have simultaneously dealt with different substrates (e.g., moraine rocks and soils) and organisms. In fact, very little is known about microbial diversity and succession on the lithic habitat (Hoppert et al., 2004; Pointing and Belnap, 2012; de los Ríos et al., 2014). Consistently with other non-polar dryland habitats (Lee et al., 2016), pioneering studies in Antarctica suggested that the edaphic and lithic niches differed in microbial community composition (Pointing et al., 2009; Makhalanyane et al., 2013; Yung et al., 2014; Van Goethem et al., 2016). Evidence based on the rock colonization rate after biocide treatments suggests that changes in lithobiontic communities may occur even over short periods of time (Cámara et al., 2011), but how these differences are established and evolve over time for different types of microorganisms remains unexplored.

To explore how microbial primary succession processes occur in different substrates in a setting devoid of vascular plants, in this study we examine the moraine rocks and soils of a retreating glacier chronosequence in maritime Antarctica. Through Illumina amplicon sequencing, we first independently assessed the diversity, structure, and temporal dynamics of succession in three microbial groups (bacteria, fungi, and algae) and then determined whether these groups showed a similar response to increasing times of being ice-free. Our working hypothesis was that, after the initial colonization of rocks and soils by a common regional pool of microorganisms (Fierer et al., 2010; Frey et al., 2013), significant differences between edaphic and lithobiontic community structure would only be apparent when more specialized communities were established later in the succession. Electron microscopy was used to identify specific features of the dynamics of moraine rock colonization at the different succession stages and also the potential contributions of lithobiontic communities to primary succession. Soil characteristics were also considered when trying to identify the drivers of community composition in the edaphic niche. The findings of this multidomain diversity study of edaphic and lithobiontic microorganisms provide insight into the microbial diversity and primary succession established in a highly threatened polar environment, as is Antarctica.

## Materials and Methods

### Study Area

**FIGURE 1:**
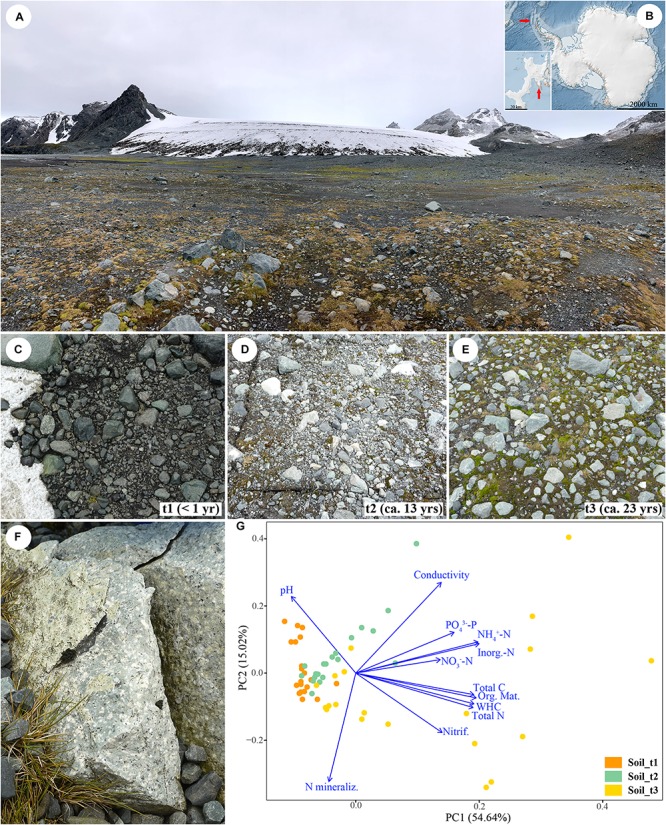
Overview of the Sally Rocks tongue of the Hurd Glacier **(A)** located on Livingston Island, South Shetland Islands, Antarctica **(B)**. Arrows indicate the geographic situation of the island (Antarctica map), and the Hurd Peninsula (inset). Sampling sites ranging from bare soils close to the glacier terminus (t1, **C**) to areas increasingly dominated by mosses that represent ca. 13 (t2, **D**) and 23 (t3, **E**) years since glacial retreat. **(F)** Detail of a sampled rock. **(G)** Ordination graph illustrating the outcome of a Principal Component Analysis (PCA) of soil data from 60 samples distributed in a three-stage chronosequence and considering 12 explanatory variables. The first two principal components explained 69.66% of total variation.

Livingston Island (62° 39′ S, 60° 21′ W) is the second largest island in the South Shetlands, a mountainous and extensively glaciated archipelago located in maritime Antarctica (Figure 1A). In contrast with continental Antarctica, the South Shetland Islands are under the effect of eastward cyclonic depression fluxes providing increased rainfall (ca. 500 mm/year), less severe temperatures, and high interannual snow variability (Bockheim and Hall, 2002; de Pablo et al., 2017). Accordingly, the climate of this archipelago can be classified as cold moist maritime which, from a phytogeographic viewpoint, facilitates the formation of semi-deserts dominated by cryptogams (mosses and lichens) with the sporadic presence of the two Antarctic phanerogams belonging to the genera *Deschampsia* and *Colobanthus* (reviewed in Peat et al., 2007). Our study focused on ice-free areas of the Livingston Island, which occupy ca. 10% of its surface and extend over the lower altitudes close to the seashore in many peninsular regions (Ferreira et al., 2017). Specifically, we investigated microbial community assembly in the forefield of the Sally Rocks tongue of the Hurd Glacier, which is part of the Hurd Peninsula ice cap (62° 39.42′ S, 60° 19.25′ W; Navarro et al., 2009). Soils in this area are classified as cryosols and their geochemical and mineralogical traits suggest they originated mainly from mechanical bedrock disintegration and also some leaching (Navas et al., 2008). According to Smellie et al. (1984), the bedrock is composed of graywackes, arenites and shales of the Miers Bluff Formation with a possible Triassic origin.

### Experimental Design

Soil and rock samples were collected during January 2014 along a three-stage chronosequence representing < 1, ca. 13 and 23 years since ice retreat. The glacier retreat dates are known because they correspond to measurements of glacier front positions determined *in situ* by the authors using Global Navigation Satellite System (<1 and 13 years) or from aerial photographs (23 years), both made during periods when the snow cover had melted away (Rodríguez-Cielos et al., 2016; Machío et al., 2017). These sites ranged from bare soils close to the glacier terminus to 23-years ice-free areas dominated by mosses (Figure 1B). At each one, we selected 20 replicate, 1 × 1 m, 4–5 m apart sampling points following a zig-zag sampling along the position of the glacier front at 2014, 2001, and 1991. One composite soil sample comprised of three randomly collected subsamples was obtained at each sampling point. The upper soil layer (0–5 cm) was sampled after removing the cryptogamic cover (mosses) and immediately preserved in RNA*later* solution (Invitrogen Corp., Carlsbad, CA, United States). Small rock fragments were excised from randomly selected moraine igneous rocks (volcanic tuff) collected in the same sampling points (Figure 1C), using a geological hammer and rock chisel that had been surface-sterilized with ethanol. Fresh soil and dry rock samples were stored in sterile Whirl-Pak^®^ plastic bags and kept at −20°C until processing. A total of 120 samples (60 rock, 60 soil) were gathered and processed in laboratory in May 2014.

### Abiotic Soil Attributes

Soils were sieved (2 mm mesh size), homogenized and analyzed for by gravimetric moisture by oven-drying the samples at 60°C to constant mass. Soil organic matter content was determined by loss on ignition at 450°C for 4 h (Nelson and Summers, 1996). Soil water holding capacity (WHC) and conductivity were estimated following Bower and Wilcox (1965) and Rey and Jarvis (2006) and respectively. Soil total carbon (C) and nitrogen (N) content were estimated by dry combustion with an elemental analyzer (LECO TruSpec CN). We measured soil pH using a pH-meter and a soil-to-water ratio of 1:2.5 (mass/volume). Soil inorganic N (NH_4_^+^-N and NO_3_^–^-N) and PO_4_^3–^-P were estimated colorimetrically from 1M KCl and 0.5M Na_2_CO_3_ extracts respectively (Moir and Tiessen, 2007; Durán et al., 2009). We carried out 12-day, constant-moisture laboratory incubations to estimate potential soil N transformation rates (Durán et al., 2017). In short, fresh soil subsamples (∼100 g) were incubated in 2 L glass jars following a temperature curve (−15°C, −10°C, −5°C, 0°C, +5°C, +10°C, +15°C) in darkness. Potential N mineralization and nitrification rates were estimated by assessing the increase in total inorganic N (NH_4_^+^-N + NO_3_^–^-N) and in NO_3_^–^-N, respectively, over the incubation period. Descriptive statistics were calculated for each variable along the chronosequence, and a Principal Component Analysis (PCA) based on a correlation matrix was performed to graphically represent measured variables and samples in an ordination biplot. Analyses were conducted in R (R Core Team, 2016). We explored the differences among successional stages using the permutational ANOVA (PERMANOVA) approach (Anderson et al., 2008) and *a posteriori* permutational pairwise comparisons using Primer 6 and PERMANOVA + (Primer-E Ltd, Plymouth, United Kingdom). A maximum of 999 permutations were used to obtain pseudo-F and *p*-values.

### DNA Extraction, PCR Amplification and High-Throughput Sequencing

Genomic DNA was extracted from rock powder obtained through mechanical grinding in an Agatha mortar as well as soil samples using the PowerSoil DNA Isolation Kit (MO BIO Laboratories, Carlsbad, CA, United States) according to the manufacturer’s protocol. DNA quality and concentration were measured using a NanoDrop ND 1000 spectrophotometer (Thermo Fisher Scientific^TM^). DNA amplification was done using the barcoding universal primers 27F and 338R spanning the V1–V2 hyper-variable regions of the bacterial 16S rRNA genes (Lane, 1991; Fierer et al., 2008), the primer pair ITS1F_KYO2–ITS2_KYO2 (Toju et al., 2012) spanning the *ITS1* region of the fungal nuclear ribosomal internal transcribed spacer, and the pair rbcL650–rbcL830 (Nozaki et al., 1995) spanning a fragment of the RuBisCO-encoding gene for eukaryotic algae. Primers had the universal tags CS1 and CS2 attached to their ends. Amplifications were done using GE Healthcare illustra^TM^ PuReTaq Ready-To-Go^TM^ PCR Beads (Amersham Biosciences) that were added to reaction mixtures containing: 3 μl DNA, 1.5 μl of each primer (10 μM) and 19 μl of sterile H2O (Sigma^TM^). Two different PCR reactions were carried out for algae, bacteria and fungi at different temperatures, in order to increase the range of organisms yielded by the PCR (Schmidt et al., 2013). The reaction conditions were as follows: denaturation at 95°C for 5 min, followed by 35 cycles (30 for bacteria) of denaturation at 94°C for 1 min, annealing at 50 and 54°C (fungi), 52 and 54°C (bacteria), and 54 and 58°C (algae) for 1 min, and extension at 72°C for 1 min (final extension at 72°C for 7 min). Amplicons obtained at different annealing temperatures were visualized by agarose gel electrophoresis and subsequently pooled at equimolar concentrations. PCR products were sent to the Unidad de Genómica (Fundación Parque Científico de Madrid, Spain) for library preparation. Individual libraries were analyzed using a Bioanalyzer 2100 (Agilent, Madrid) to estimate the size and concentration of the specific PCR products. Individual barcodes were appended to each sample. Paired-end sequencing was performed on an Illumina MiSeq sequencer (2 × 250 bp, San Diego, CA, United States) using the MiSeq Reagent Kit (v3). Raw reads were demultiplexed and barcode sequences were removed by the sequencing center.

### Analysis of Bacterial 16S rRNA and Algal *rbcL* Data

Illumina data for bacteria and algae were analyzed using QIIME v.1.9.1 (Caporaso et al., 2010) following a standard operating procedure implemented in the Microbiome Helper virtual box (Comeau et al., 2017)^1^ with some modifications. Briefly, overlapping paired-end reads were merged using PEAR v.0.9.19 (Zhang et al., 2014). Subsequently, FastQC (Andrews, 2010) was used to calculate quality metrics for the stitched reads, and then the FASTx-Toolkit (Gordon and Hannon, 2010) was run to filter out reads that had an average quality score less than Q28 over at least 90% of the read. In addition, we filtered out reads shorter than 280 bp (bacteria) or 120 bp (algae) that did not contain matching 5′ and 3′ sequences to the appropriate forward and reverse primers with BBMap v.35.85 (Bushnell, 2014), as well as reads with any ambiguous base (“N”). Primer sequences were also trimmed from read ends. Approximately 82% (bacteria) and 90% (algae) of reads were retained per sample. Then, *de novo* and reference-based chimera checking were combined to screen out potential chimeric reads using VSEARCH v.1.11.1 (Rognes et al., 2016). Lastly, non-chimeric reads showing an extremely long sequence length (>320 bp bacteria; >150 bp algae) were removed using SeqKit (Shen et al., 2016) to avoid the inference of spurious Operational Taxonomic Units (OTUs). This step removed ca. 1.7% (bacteria) and 4.7% (algae) of all non-chimera sequences.

Following these filtering steps, the remaining sequences were used for open-reference OTU picking (Rideout et al., 2014) at 97% genetic similarity cut-off. For this purpose, we used QIIME scripts hosted in the Microbiome Helper framework (Comeau et al., 2017). Specifically, SortMeRNA v.2.0 (Kopylova et al., 2012) was used for the reference OTU picking steps (with sortmerna_coverage = 0.8), and SumaClust v.1.0.00 (Mercier et al., 2013) for the *de novo* OTU picking steps (with 10% of the failures subsampled). Taxonomic affinities for representative bacterial OTU sequences were simultaneously assigned based on the SILVA database (release 128, Quast et al., 2012). Algal taxonomic profiles were retrieved from a customized *rbcL* sequence database generated from GenBank data. We then removed OTUs that contained less than 0.1% of the total sequences in order to compensate for MiSeq run-to-run bleed-through (Comeau et al., 2017). Thus, OTUs with more than 67 (bacteria) and 74 (algae) sequences were kept. Finally, OTUs assigned to sequences from eukaryotic organelles (mitochondria or chloroplast) in the bacterial dataset were filtered out. Scripts and used commands are available in Supplementary Data Sheet S1.

### Analysis of Fungal *ITS1* Data

Fungal data were processed with the automated bioinformatics pipeline PIPITS v. 1.5.0 (Gweon et al., 2015). Briefly, raw read pairs were joined at the overlapping region and then quality filtered with the FASTx-Toolkit. The *ITS1* region was extracted with ITSx (Bengtsson-Palme et al., 2013). Subsequently, short (<100 bp) and unique sequences (singletons) were filtered out to avoid technical artifacts causing an overestimate of the number of species (Dickie, 2010), and the remaining sequences were clustered into operational taxonomic units (OTUs) at 97% sequence similarity with VSEARCH v.2.4.3 (Rognes et al., 2016). Identification and filtering of chimeric sequences was done with UCHIME v.4.2.40 (Edgar et al., 2011) using the UNITE database as reference (update 28 June 2017, Kõljalg et al., 2013). The taxonomic assignment of OTUs was performed with the RDP naïve Bayesian Classifier (Wang et al., 2007) based also on the latest version of the UNITE database. Thereafter, the OTU abundance table obtained from PIPITS was selected for downstream analyses. These were done in QIIME v.1.9.1 (Caporaso et al., 2010) and consisted first in improving the resolution of taxonomic assignments based on a GenBank-customized database for fungal *ITS1* using the script *assign_taxonomy.py*. Then, the OTUs assigned to organisms other than fungi, the unclassified, and those considered to have a low confidence (i.e., identified by fewer than 0.1% of the reads, which is the estimated amount of sample bleed-through between runs on the Illumina Miseq, Comeau et al., 2017) were removed from the final dataset. As a consequence, fungal OTUs with more than 66 sequences were kept.

### Amplicon Sequence Variant (ASV) Identification

The Divisive Amplicon Denoising Algorithm (DADA2, Callahan et al., 2016) was used to infer Amplicon Sequence Variants (henceforth ASVs) that differed from each other at least by a single nucleotide. Therefore, by avoiding binning reads coarsely into OTUs, the analysis of microbial community composition and succession patterns in the edaphic and lithic niches could be performed at a finer resolution (Callahan et al., 2017). Abundance tables containing inferred variants and read counts per samples for bacteria, fungi and algae were obtained using R scripts available in the Microbiome Helper virtual box (Comeau et al., 2017)^2^. For the fungal data, we added an ITSx extraction step with ITXs (Bengtsson-Palme et al., 2013) using a script made available by J. L. Darcy at GitHub^3^. These tables were converted into BIOM format, and then were further processed to remove extremely short/long bacterial and algal sequences with SeqKit (Shen et al., 2016), and to assign taxonomy metadata to ASVs based on the same databases mentioned above. We also filtered the final ASV tables by removing variants that represented < 0.005% of the total read abundance on a per-sample basis (Callahan et al., 2016; Dal Grande et al., 2018). All these last steps were implemented under the QIIME v.1.9.1 (Caporaso et al., 2010) framework. Scripts and used commands are available in Supplementary Data Sheet S1.

### Microbial Diversity and Statistical Analyses

Taxonomic profiles of microbial communities at the level of phylum (bacteria, fungi), class (fungi) and order (algae) were generated using default settings in QIIME v.1.9.1 (see Supplementary Data Sheet S1). The numbers of OTUs and ASVs that were shared between substrate types and succession stages were calculated with *jvenn* (Bardou et al., 2014). To identify OTUs and ASVs that were differently abundant in rock and soil, we first plotted taxa abundances across sample groups (lithic vs. edaphic niches) and then compared them with the non-parametric Wilcoxon-rank test. Additional support for differential abundances was computed with DEseq2 (Love et al., 2014). Alpha-diversity metrics (OTU richness, Pielou’s Evenness, Shannon and Simpson indices, and Faith’s phylogenetic diversity, PD) were calculated based on community matrices with a rarefied read depth of 38530 (bacteria), 13520 (fungi), and 32665 (algae). Samples with extremely low sequencing depth (outliers) were also removed prior to these calculations. It must be noted that Faith’s PD could only be computed for bacteria and algae because the high number of polymorphisms in fungal ITS sequences precluded building a reliable alignment. Beta-diversity analyses used OTU tables normalized by the cumulative sum scaling (CSS) method, which has been shown to outperform other methods for marker gene survey data (Paulson et al., 2013). Non-metric multidimensional scaling (NMDS) ordinations were computed on the basis of Bray-Curtis dissimilarities to illustrate differences in the composition of microbial communities across sample categories. To generate statistical support for these differences, we ran ANalysis Of SIMilarities (ANOSIM; Clarke, 1993) as well as the non-parametric Adonis test (Anderson, 2001) with 999 permutations. To evaluate if substrates differed in their dispersion, an analysis of multivariate homogeneity (PERMDISP, Anderson, 2006) was run with the function ‘betadisper’ implemented in the R package “vegan” (Oksanen et al., 2016) using default parameters. Beta-diversity was further surveyed to quantify the turnover (i.e., OTU replacement) and nestedness (i.e., OTU loss) along the three-stage chronosequence independently for each niche with the function ‘beta.multi’ of the R package “betapart” using the Sørensen dissimilarity index (Baselga and Orme, 2012; Baselga, 2017). Lastly, beta-phylodiversity was assessed using unweighted and weighted UniFrac distance matrices (Lozupone and Knight, 2005) and visualized through NMDS. Methods used to analyze alpha- and beta-diversity in ASV datasets recapitulated those used for OTUs. All analyses were conducted in the Microbiome Helper (Comeau et al., 2017) and Calypso v.8.56 software tool (Zakrzewski et al., 2016)^4^ environments unless otherwise indicated. All tests regarded p-values below 0.05 as significant.

Relationships between environmental variables and soil microbial community structure were examined and graphically represented using a distance-based redundancy analysis (db-RDA, Legendre and Anderson, 1999). Prior to building the constrained ordination plot, each environmental variable was inspected to determine whether they required transformation. Thus, organic matter, NH_4_^+^-N, inorganic N, conductivity, and total C and N were log-transformed. The remaining variables were left untransformed. Then, significant relationships between OTU-based Bray-Curtis similarity matrices and environmental variables were subjected to a Mantel test, using the Spearman correlation method and 999 permutations. The BIOENV analysis was performed to identify the subset of environmental variables with the best correlation to community data considering also the possible collinearity between them. Finally, a db-RDA was computed for each studied microbial group, and to assess the significance of the individual axes and environmental variables, an ANOVA test based on 500 permutations was run. All analyses were conducted in R using the package “vegan” (Oksanen et al., 2016) and the used scripts are available in Supplementary Data Sheet S1.

### Electron Microscopy Analysis of Lithobiontic Communities

Colonized rock samples were processed for scanning electron microscopy in backscattered electron mode (SEM-BSE) following (Wierzchos and Ascaso, 1994). Briefly, rock-colonized fragments were fixed in glutaraldehyde (3% v/v) and osmium tetroxide solutions (1% w/v), dehydrated in a graded ethanol series (from 30 to 100% v/v) and embedded in LR White resin. Blocks of resin-embedded rock-colonized samples were finely polished, carbon-coated and observed using a FEI INSPECT microscope.

## Results

### Microbial Diversity, Community Structure and Succession

120, 114, and 88 positive amplifications were achieved for bacteria, fungi, and algae respectively (further details on Illumina sequencing shown in Supplementary Table S1). PCR amplifications using fungal and algal-targeted primers were unsuccessful for some rock samples from the first succession stage (the area closest to the glacier front). In general, our sequencing achieved average coverage of more than 50000 high-quality sequences per sample (Supplementary Table S2). Furthermore, rarefaction curves indicated that sequencing depth was sufficient to identify the majority of OTUs and ASVs within microbial communities at each succession stage and niche (Supplementary Figures S1, S2).

**FIGURE 2:**
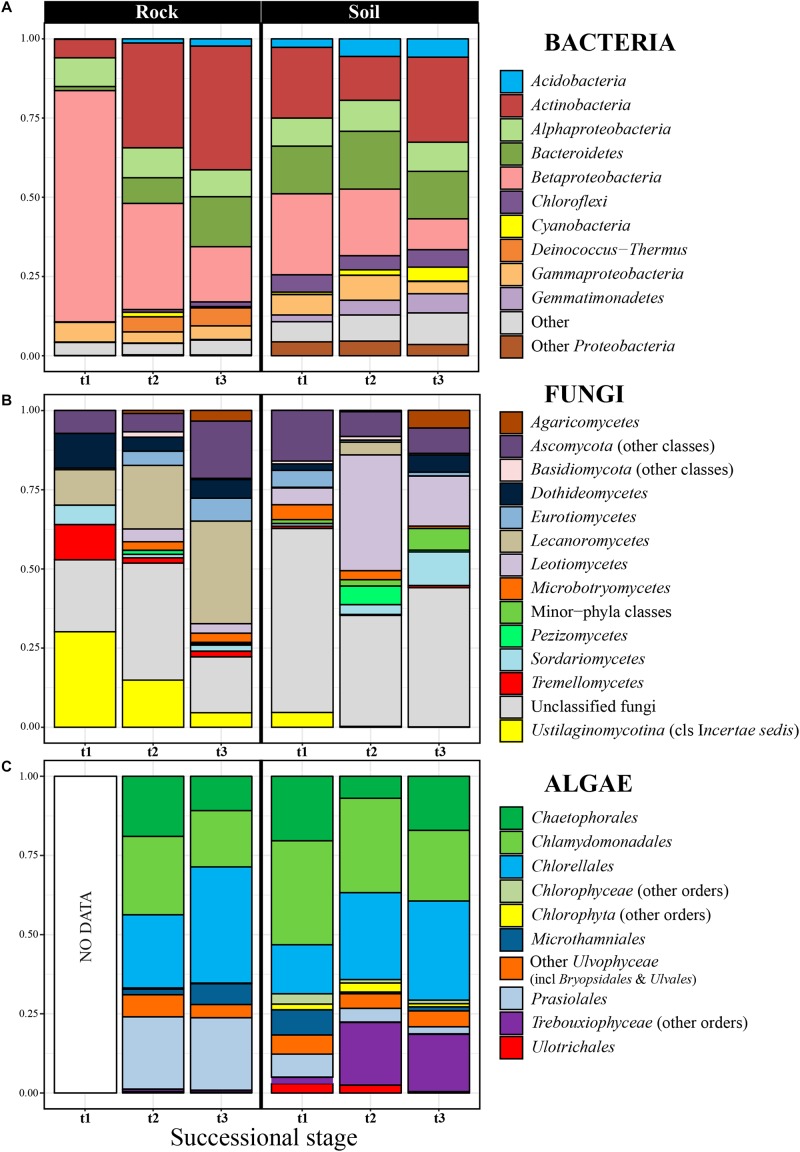
Relative abundance of bacterial phyla **(A)**, fungal classes **(B)**, and algal orders **(C)** in rocks and soil at the three considered successional stages. Each taxon proportion was calculated as the arithmetic mean of several replicate samples (see Supplementary Figures S3, S7, S8 to check proportions per replicate). The bacterial phylum *Proteobacteria* was explicitly subdivided into its three most abundant classes in our dataset (*Alpha*-, *Beta*- and *Gammaproteobacteria*).

The 5070 bacterial OTUs inferred for the entire Hurd Glacier forefield (Sally Rocks tongue, Figure 1) segregated in 31 phyla, the most abundant being *Proteobacteria* and *Actinobacteria* (up to 71% of total diversity), followed by *Bacteroidetes*, *Chloroflexi*, *Acidobacteria*, *Deinococcus*-*Thermus*, *Gemmatimonadetes*, and *Cyanobacteria* (Figure 2A and Supplementary Figure S3). The *Blastocatellia* and *Solibacteres* (*Acidobacteria*), KD4-96 (*Chloroflexi*), *Cyanobacteria* and *Gemmatimonadales* (*Gemmatimonadetes*) were differentially more abundant in edaphic than lithobiontic communities, and the reverse was true for the order *Deinococcales* within *Deinococcus*-*Thermus* (Supplementary Figures S3B, S4A, S5A). The orders *Holophagales* and *Acidobacteriales* (phylum *Acidobacteria*) were only present in soils. In the edaphic communities, the relative abundance of *Burkholderiales* (*Betaproteobacteria*) and *Flavobacteriia* (*Bacterioidetes*) decreased along the chronosequence, whereas *Gemmatimonadales* increased (Figure 2A and Supplementary Figure S3). Similarly, the relative abundance of *Burkholderiales* showed a steep decrease along the rock chronosequence, whereas the relative abundance of the *Actinobacteria* (especially the *Micrococcales* and *Propionibacteriales*), *Flavobacteriia*, and *Cytophagia* (*Bacteroidetes*) increased toward the latter succession stage.

Nine fungal phyla were encountered among the 1210 OTUs found in the edaphic and lithobiontic communities. Of these, the most abundant phylum was the *Ascomycota* (47%), with *Lecanoromycetes* (*Caliciaceae*, *Umbilicariaceae*, and *Lecanoraceae*) and *Leotiomycetes* (*Myxotrichaceae* and *Pseudeurotiaceae*) as dominant classes and families (Figure 2B and Supplementary Figures S6, S7). The genera of lichenized ascomycetes *Amandinea*, *Austroplaca*, *Buellia*, *Mastodia*, *Umbilicaria*, and *Verrucaria*, and the non-lichenized *Antarctomyces* and *Pseudogymnoascus* showed relevant abundances (data not shown). The *Basidiomycota* comprised the 14% of OTUs, dominating the classes *Agaricomycetes*, *Microbotryomycetes* and an *incertae sedis* group within *Ustilaginomycotina* including the family *Malasseziaceae*. The *Ascomycota* and *Basidiomycota* showed similar abundance in communities from both habitats, while basal phyla such as the *Chytridiomycota*, *Morteriellomycota*, and *Rozellomycota* prevailed in edaphic communities (Supplementary Figure S6). At the class level, the *Agaricomycetes*, *Leotiomycetes* and *Microbotryomycetes* were differentially more abundant in edaphic communities than in lithobiontic ones, but the relative abundance of *Lecanoromycetes* was higher in the latter (Supplementary Figures S4B, 5B). Succession patterns in edaphic communities were found only for the *Microbotryomycetes* and *Sordariomycetes* which decreased and increased respectively along the chronosequence (Figure 2B and Supplementary Figure S7). The *Eurotiomycetes* and *Lecanoromycetes* were more abundant in the intermediate succession stage, whereas the *Agaricomycetes* in the latest succession stage. In contrast, in lithobiontic communities, a clear increase in abundance with time was found for *Eurotiomycetes* and *Lecanoromycetes* and the reverse pattern for *Tremellomycetes* and *Ustilaginomycetes*. The proportion of inferred OTUs that could not be taxonomically classified below Kingdom level was high in general (ca. 37%) and at each succession stage.

The inferred 512 algal OTUs were assigned to the *Chlorophyta* and *Streptophyta*. The most abundant *Chlorophyta* orders were the trebouxiophycean *Chlorellales*, *Chaetophorales*, *Prasiolales*, and *Microthamniales* and the chlorophycean *Chlamydomonadales* (Figure 2C and Supplementary Figure S8). The *Prasiolales*, *Ulotrichales*, and *Ulvales* were differentially more abundant in lithobiontic than in edaphic communities, whereas the abundance of *Bryopsidales* and *Sphaeropleales* was higher for edaphic communities (Supplementary Figures S4C, S5C). The relative abundance of *Microthamniales* and *Prasiolales* decreased with time in edaphic communities, opposed to the increase shown by other *Trebouxiophyceae* orders. In lithobiontic communities, the *Microthamniales* was markedly more abundant in the latest succession stage (Figure 2C and Supplementary Figure S8).

In order to gain additional insight on microbial diversity, we also assessed it using ASVs. Estimated diversity for bacteria, fungi and algae were 14994, 2004, and 630 ASVs respectively. Subtle differences in the proportion of the some taxonomic groups were detected when comparing community structure using ASVs and OTUs (Supplementary Figures S3, S6–S8). Due to the assignment based upon absolute sequence similarity, the percentages of shared ASVs between the edaphic and lithic niches were markedly lower than those obtained using OTU data (Table 1). Specifically, the number of shared bacterial and algal OTUs in these two communities was over 50%, while in the alternative analysis they shared less than 16% of ASVs. This was reflected for all successional stages in this study (Supplementary Figure S9). The analysis of OTU data showed that most lithobiontic bacteria and fungi were unique to, or shared between the latter two stages, and that a high proportion of soil bacterial, fungal and algal OTUs were shared among the three succession stages.

**TABLE 1:** Proportion of OTUs and ASVs that are unique to either the edaphic or lithic niche, or shared between these niches.

	**Bacteria**			**Fungi**			**Algae**		
	**(OTUs: 5070; ASVs: 14994)**			**(OTUs: 1210; ASVs: 2004)**			**(OTUs: 512; ASVs: 630)**		
			
	**Unique to Rock**	**Unique to Soil**	**Shared**	**Unique to Rock**	**Unique to Soil**	**Shared**	**Unique to Rock**	**Unique to Soil**	**Shared**
OTUs	3.2	31.5	65.3	11.4	51.6	37	2.2	7.8	90
ASVs	17.7	72.1	10.2	13.6	71.9	14.5	50.8	33.5	15.7

**FIGURE 3:**
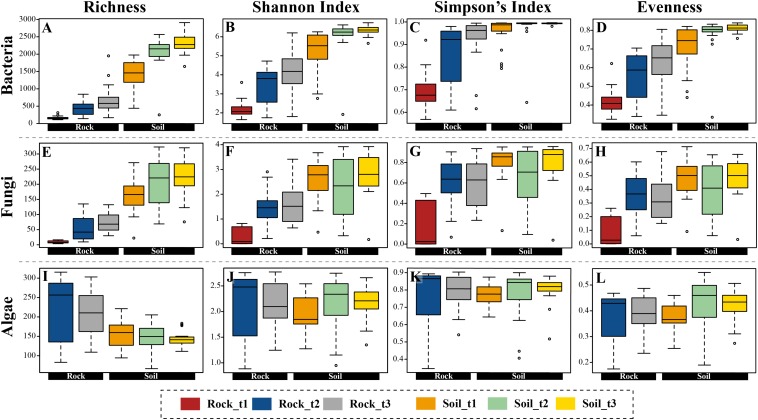
Boxplots representing four alpha-diversity estimators (richness, Shannon and Simpson indices, and evenness) for bacteria **(A–D)**, fungi **(E–H)**, and algae **(I–L)** calculated with OTU data, and arranged according to substrate type (rocks, soil) and successional stage.

General indicators of diversity, including richness, Shannon and Simpson indices (Figure 3 and Supplementary Figure S10) and Faith’s PD (Supplementary Figure S11) were higher in edaphic than in lithobiontic bacterial and fungal communities (*p*-value < 0.05), and increased over distance from glacial terminus in both types of communities. For algae, these estimators were comparable for both substrates, except for OTU richness which was higher in lithobiontic than in edaphic communities (*p*-value < 0.05). Finally, whereas soil communities of bacteria and fungi tended to be more even in terms of taxon contribution to overall abundance than lithobiontic communities, a clear pattern of increasing evenness through time in both niches was only revealed for bacteria (Figure 3).

**FIGURE 4:**
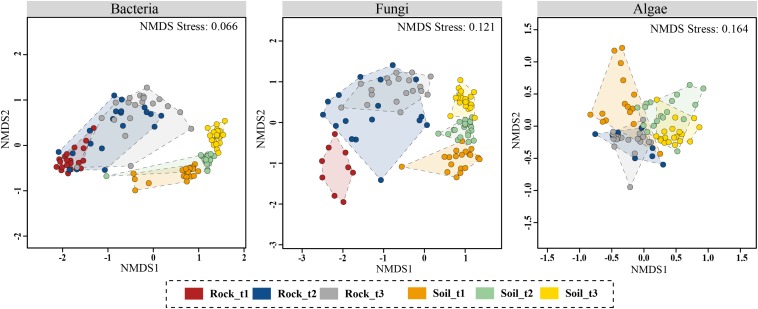
Non-metric multidimensional scaling (NMDS) ordination plots of Bray–Curtis dissimilarities for bacterial, fungal and algal communities across sample categories (i.e., substrate type plus successional stage). Analyses were based on OTU data matrices. Results using ASV data are Supplementary Figure S12.

NMDS ordinations constructed based on Bray–Curtis dissimilarities confirmed that bacterial and fungal community structure differed between moraine rocks and soil in the Hurd Glacier forefield (Figure 4 and Supplementary Figure S12). Community differentiation between substrates was corroborated statistically for all three organismal groups (see ANOSIM and ADONIS results in Table 2). These statistical tests also revealed differentiation among edaphic and lithic fungal and bacterial communities at different succession stages, except for lithobiontic algal communities. Furthermore, sample ordinations incorporating phylogenetic distance data between observed bacterial and algal OTUs revealed substrate-related community differentiation as well (see UniFrac NMDS, Supplementary Figure S13). In general, the different ordination plots illustrated contrasting levels of sample dispersion between niches. Multivariate homogeneity tests indeed showed that lithobiontic bacterial and fungal communities were considerably and significantly more variable in their intra-OTU composition than the edaphic ones (Supplementary Figure S14). The opposite pattern was detected for algae, but it needs to be interpreted with caution because only two successional stages were analyzed for this group of microorganisms. Finally, variation in OTU composition along the investigated chronosequence for the three organismal groups showed moderate (lithic niche) to low (edaphic niche) β_SOR_ values (Table 3). The turnover (β_SIM_) component dominated variation in fungal and algal edaphic communities, whereas the nestedness (β_SNE_) did so in bacterial lithobiontic communities. Comparable contributions of turnover and nestedness were found for fungal lithobiontic and bacterial edaphic communities. Algal composition dissimilarities in rocks were negligible.

**TABLE 2:** ANOSIM and Adonis tests results based on OTU and ASV data.

	**Data used**	**ANOSIM Global test**		**Adonis test**	
		***R***	***p*-value**	***R*^2^**	***p*-value**
**Overall edaphic and lithic communities**					
Bacteria	OTUs	0.807	0.001	0.295	0.001
	ASVs	0.802	0.001	0.179	0.001
Fungi	OTUs	0.746	0.001	0.174	0.001
	ASVs	0.698	0.001	0.131	0.001
Algae	OTUs	0.335	0.001	0.147	0.001
	ASVs	0.381	0.001	0.138	0.001
**Edaphic communities across the three-stage chronosequence**					
Bacteria	OTUs	0.718	0.001	0.388	0.001
	ASVs	0.728	0.001	0.287	0.001
Fungi	OTUs	0.831	0.001	0.352	0.001
	ASVs	0.805	0.001	0.275	0.001
Algae	OTUs	0.622	0.001	0.288	0.001
	ASVs	0.713	0.001	0.328	0.001
**Lithic communities across the three-stage chronosequence**					
Bacteria	OTUs	0.531	0.001	0.287	0.001
	ASVs	0.494	0.001	0.258	0.001
Fungi	OTUs	0.524	0.001	0.167	0.001
	ASVs	0.437	0.001	0.125	0.001
Algae	OTUs	0.02	0.198	0.036	0.238
	ASVs	0.048	0.078	0.045	0.112

*Tests were focused on revealing variation in bacterial, fungal and algal composition in overall edaphic and lithic communities, and in the edaphic/lithic communities across the three-stage chronosequence in the Hurd Glacier forefront.*

**TABLE 3:** Compositional dissimilarities among bacterial, fungal and algal communities developing on rocks and soils.

	**Lithobiontic community**			**Edaphic community**		
		
	**Bacteria**	**Fungi**	**Algae**	**Bacteria**	**Fungi**	**Algae**
β_βSOR_	0.480	0.576	0.029	0.175	0.280	0.186
β_βSIM_	0.160	0.291	0.027	0.094	0.245	0.158
β_βSNE_	0.320	0.285	0.002	0.081	0.035	0.028

*Analyses were done using OTU matrices. Rows: β_βSOR_, *Sørensen* index of compositional dissimilarity; β_βSIM_, turnover component of compositional dissimilarity; β_βSNE_, nestedness component of compositional dissimilarity.*

### Soil Attributes and Edaphic Microbial Communities

The soil chronosequence was characterized by increases in organic matter, total C and N content, as well as in inorganic N and P pools, potential nitrification rates and WHC with time since exposure (Supplementary Table S3 and Supplementary Figure S15). In contrast, the chronosequence displayed a decreasing pH with time since exposure (Supplementary Figure S15). The PCA identified clear patterns in soil sample attributes along the first principal component (PC1) explaining 54.64% of the variation in sample data (Figure 1D); thus, samples of each successional stage grouped together, although those belonging to the late successional stage were highly dispersed. Inorganic N, NH_4_^+^-N, organic matter, WHC and total C and N were the most important variables (high correlation with PC1). The lower-order PC2 explained 15.02% of the variation in sample abiotic data. PC2 seemed related to N mineralization, pH and conductivity.

**FIGURE 5:**
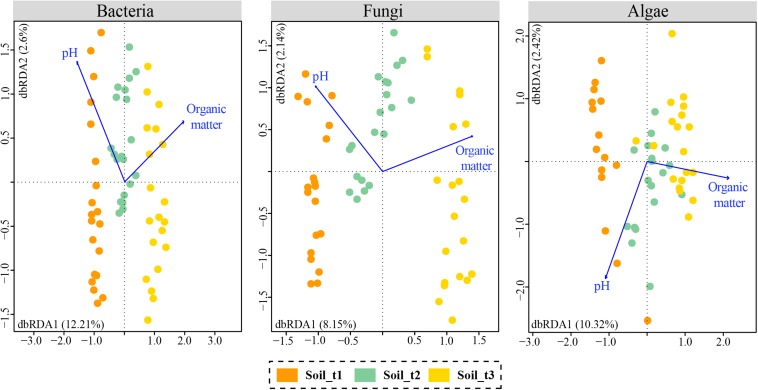
Distance based redundancy analyses (db-RDAs) with selected edaphic variables that explained most of the variability in soil communities of bacteria, fungi and algae. Samples of each of the three successional stages are colored differently.

A Mantel test revealed overall significant relationships between the recorded soil attributes and community OTU composition (*p*-value < 0.05, Supplementary Table S4). According to BIOENV analyses, the variables with the highest correlation with OTU communities’ composition were organic matter and pH for bacteria and fungi, and organic matter for algae (Supplementary Table S5). Db-RDA analyses were performed using these selected variables (Figure 5). The variable pH had to be added to the ordination of algal data for effective calculation of the two dbRDA constrained axes. Total variation in bacterial, fungal and algal data explained by dbRDA1 and dbRDA2 were 14.81, 10.29, and 12.74%, respectively. The three separate db-RDA graphs revealed that organic matter and to a lesser extent pH were correlated with axis 1 which explained most of the variability in microbial communities and separated samples of each successional stage in the studied glacier forefield. Results of ANOVA tests assessing the significance of the individual axes and environmental variables are in Supplementary Table S6.

### Atmosphere-Lithosphere Interface

**FIGURE 6:**
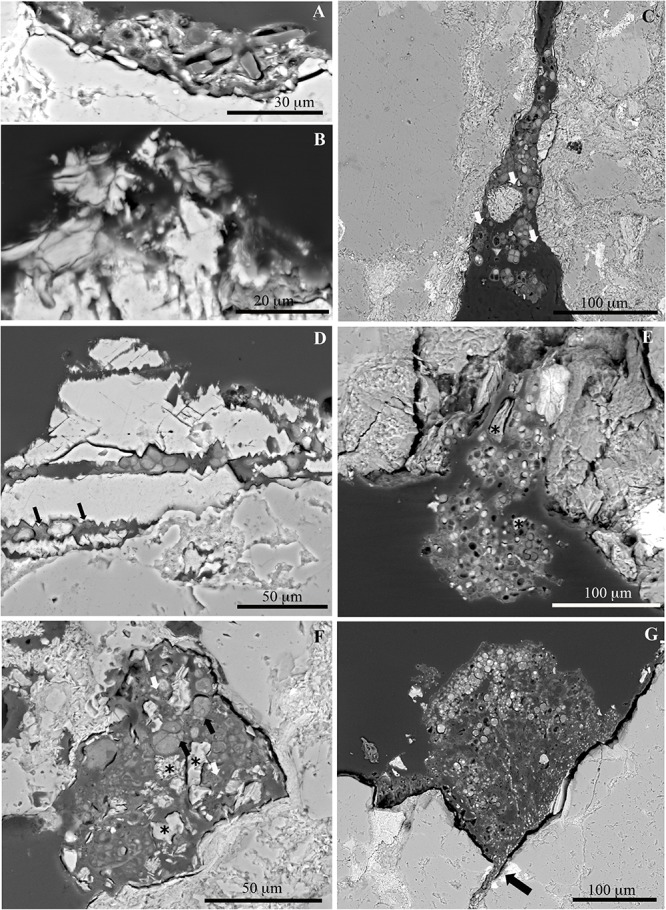
SEM-BSE images of different succession stages of microbial and lichen colonization of moraine rocks. Diatom cells **(A)** and grain mineral matrix **(B)** situated on the rock surface at rocks close to the glacier forefront. Endolithic cyanobacteria-dominated communities **(C)** and algal communities **(D)** at rocks being ice-free for ca. 13 years. Arrows indicate the presence of non-photosynthetic bacterial cells in both microbial communities. Endolithic cyanobacteria-dominated communities in fissures **(E)**, and mixed microbial communities inside epilithic cavities **(F)** of rocks being ice-free for ca. 23 years; these contain small mineral fragments (^∗^) which could be the result of microbial-mineral interactions. Black arrows in **(F)** indicate lichen mycobiont-photobiont interactions. Colonization of an epilithic lichen thallus on a rock being ice free for ca. 23 years **(G)**, showing endolithic penetration of mycobiont cells (black arrow).

Consistent colonization of rocks from the area close to the glacier front was not observed, and only some scattered epilithic microbial cells were detected in some samples (Figure 6A), which might correspond with deposited cells coming from the glacier. However, the presence of other indistinguishable small microbial cells immersed in the fine grain mineral matrix observed in the rock surface cannot be discarded in some samples (Figure 6B). Lithobiontic communities were frequently found in endolithic habitats of rocks being ice-free for 13 years (Figures 6C,D), and were dominated by photosynthetic microorganisms. Non-photosynthetic bacteria were closely associated to cyanobacteria (white arrows in Figure 6C) and algal cells present in the rock fissures (black arrows in Figure 6D). Endolithic colonization was revealed in existing rock fissures or pores (Figure 6C) at this succession stage, with crowded microbial cells filling totally some of them (Figure 6D) and multiple close microbial-mineral interactions (Figures 6C,D). Morphologically more diverse microbial communities were detected on rocks from areas being ice-free for 23 years. Some of these communities were dominated by cyanobacteria (Figure 6E), but in other areas, cyanobacterial cells coexisted with algal and fungal cells (black arrows in Figure 6F). These mixed communities were detected especially in endolithic positions. Some cavities and fissures were occupied by algal-fungal lichen associations (Figure 6F). Interactions between algal and fungal partners were extensively found (e.g., penetrating haustoria, black arrows in Figure 6F). Non-photosynthetic bacteria were also present in the proximity of algal and fungal cells (white arrows in Figure 6F). In these colonized cavities, it also frequent to observe small mineral fragments (asterisks in Figures 6E,F) which could be the result of microbial-mineral interactions. Epilithic lichen thalli were also observed in some rocks from the latest succession stage (Figure 6G). Mycobiont cells were observed penetrating through thin fissures under the lichen thalli (arrow in Figure 6G), thus revealing clear impacts of fungal cells on the rock surface.

## Discussion

Our results showed, for the first time, that communities of bacteria, fungi and algae assemble differently in moraine rocks and soils during primary succession in a geographically singular and highly vulnerable ecosystem: the forefield of the Sally Rocks glacier tongue (Hurd Glacier, Livingston Island, maritime Antarctica). Although the number of works exploring microbial diversity and succession dynamics in glacier forefields has increased in recent years, especially for bacteria, to our knowledge no studies had yet compared how the three studied organismal groups assemble simultaneously in the lithic and edaphic niches and whether differences in community structure between these were constant over time.

The comprehensive analyses of the microbiome of rocks and soils revealed that the *Proteobacteria* and *Actinobacteria* were two of the most abundant bacterial phyla, as occurs with other (non-)polar and alpine retreating glacier forefields (Babalola et al., 2009; Zumsteg et al., 2012; Bajerski and Wagner, 2013; Makhalanyane et al., 2013; Brown and Jumpponen, 2014; Pessi et al., 2015; Fernández-Martínez et al., 2017; Jiang et al., 2018). These classes are known to include a number of phototrophic, photoheterotrophic and chemolithotropic taxa able to thrive in the oligotrophic niches represented by recently exposed rocks and soils (Kersters et al., 2006; Rime et al., 2016; Wei et al., 2016). Our data showed an uneven and generally low abundance of *Cyanobacteria*, a scenario in agreement with results of Ji et al. (2016), who revealed low abundances of *Cyanobacteria* coupled with a predominance of *Chloroflexi*, *Actinobacteria* and *Proteobacteria* in ice-free soils in the Mitchell Peninsula (East Antarctica). These results contrast with findings of previous studies in Antarctica and elsewhere (Hodkinson et al., 2003; Pointing et al., 2009; Liu et al., 2016; Archer et al., 2017; Fernández-Martínez et al., 2017), although these included different substrates with often unknown exposure times. This may be indicating that, at least in our studied Antarctic glacier forefield, *Cyanobacteria* require longer times after ice retreat to settle more profusely. This is especially true for the lithic niche, as suggested by our results of electron microscopy showing colonization of these autotrophic bacteria only after ∼13 years since glacial retreat. Alternatively, it is also likely that in our study area these bacteria colonize other non-sampled microhabitats such as the hypolithic (e.g., Cowan et al., 2010; Wei et al., 2016), or are associated with moss-dominated cryptogamic covers (e.g., Arróniz-Crespo et al., 2014). It is however remarkable that the *Burkholderiales* (a group of diazotroph *Betaproteobacteria*) were more abundant at the first successional states of both substrates, suggesting that these bacteria could play an important role for initial colonization through nitrogen fixation.

Our data showed that, among the studied microeukaryotic groups, the fungal phyla *Ascomycota* and *Basidiomycota* dominated the study area likely due to the highly diverse ecological functions they can play: decomposers, parasites, or mutualists (Yergeau et al., 2007; Honegger, 2012; Tedersoo et al., 2014; Wei et al., 2015). The unraveled taxonomic diversity is in accordance with previous studies that used culture-dependent and high-throughput sequencing data to explore several Antarctic habitats (Bridge and Newsham, 2009; Arenz et al., 2014; Baeza et al., 2017). For instance, various genera of (non-)lichenized ascomycetes detected in our analyses had already been reported in the same or nearby regions in morphology-based and culture-dependent studies (Sancho et al., 1999; Søchting et al., 2004; Bridge et al., 2009; Turchetti et al., 2011). It may be highlighted that electron microscopy revealed that lichen associations were already present in rocks that otherwise showed no apparent colonization by lichen thalli on their surface. The lower relative abundance of *Chytridiomycota* parallels with that of *Cyanobacteria*, and this correlation makes all sense since the latter are common hosts of numerous chytrid species (Gerphagnon et al., 2013; Ishida et al., 2015). Interestingly, we recovered a remarkable proportion of fungal phylotypes with no taxonomic affiliation below Kingdom level, an issue found in other fungal metabarcoding studies using *ITS1* (Cox et al., 2016; Archer et al., 2019). This is likely due to a combined effect of the lack of sequence data for many already described (Antarctic) fungi, and the existence of potential endemic taxa new for science (Bridge and Spooner, 2012).

The different groups of chlorophyte algae found in our study seem to be ubiquitous in deglaciated areas (Fernández-Martínez et al., 2017 and references therein), and are shared with polar biological soil crusts (Rippin et al., 2018). Several green algal lichen photobiont genera were found in our dataset, such as *Trebouxia*, *Prasiola*, *Diplosphaera*, and *Chloroidium* (Darienko et al., 2010; Thüs et al., 2011; Garrido-Benavent et al., 2017). Some of these genera have also been found free-living in lithic niches (Yung et al., 2014) and at subglacial and exposed geothermal sites (Fraser et al., 2018) in Continental Antarctica. Other free-living algae detected may constitute cysts or propagules of ice- and snow-dominating groups such as the *Chlamydomonadales* and *Chlorellales* (Brown et al., 2016; Anesio et al., 2017). Moreover, the presence of algal phylotypes of typical marine intertidal and shallow subtidal zones belonging into the *Bryopsidales* and *Ulvales* (Pellizzari et al., 2017) may be explained by the unique geographical display of our studied glacier foreland, located near the seashore.

Our results demonstrated that lithobionthic and edaphic bacterial community differentiation occurs along the studied chronosequence at both the taxonomic and community structure levels in maritime Antarctica. These results are in line with previous findings by Pointing et al. (2009), Makhalanyane et al. (2013), Yung et al. (2014), and Van Goethem et al. (2016), which compared lithic and edaphic mature communities in climatically more severe Antarctic regions. Further, our study showed that differences in community composition were evident even at the earliest succession stage. Differential selection for taxa in the two niches is likely related to intrinsic (e.g., metabolism or physiology) and extrinsic (e.g., microclimate and biotic interactions) factors (Chan et al., 2013; Makhalanyane et al., 2013; Cámara et al., 2014; de los Ríos et al., 2014). For example, the predominant phyla *Chloroflexi* and *Acidobacteria* in the studied soils, which may have a crucial ecological role in contributing to nutrient input through anoxygenic photosynthesis (Bryant et al., 2012; Ji et al., 2016), are likely favored by occasional flooding due to ice melting. Similarly, the observed dominance of *Gemmatimonadetes* in the edaphic niche, supported by previous findings by Niederberger et al. (2008) and Babalola et al. (2009), may be linked to their important role in soil biogeochemical processes through phosphorous metabolism (Zhang et al., 2003), hence assisting early ecosystem development in this deglaciated area (Knelman et al., 2014), as confirmed by the observed increases in soil phosphate availability with time. In contrast, the phylum *Deinococcus*-*Thermus* and the *Sphingomonadales* (*Alphaproteobacteria*) were significantly more abundant on rocks, where they may benefit from using sugar alcohols and organic acids released by lichenized fungi, molds and algae (Siebert and Hirsch, 1988; Brewer and Fierer, 2018).

Similar intrinsic and extrinsic factors may be invoked when discussing the edaphic and lithic community differentiation that was revealed for the microeukaryotic groups. Thus, we found that the *Agaricomycetes*, *Leotiomycetes*, and *Microbotryomycetes* (basidiomycete yeasts) were more abundant on the edaphic niche. Members of these classes (e.g., *Agaricales*, *Helotiales*, and *Thelebolales*) are likely acting as saprotrophs, i.e., decomposers of organic matter, and not as mycorrhizal partners because the studied landscape is virtually devoid of vascular plants (Jumpponen, 2003; Dong et al., 2016). Together with yeasts, which are chemoheterotrophic, these fungi seem to be prevalent even close to glacial terminus, where our analyses of soil attributes indicated low organic matter and total carbon contents. As argued by Zdanowski et al. (2013), which studied the forefield Ecology Glacier (King George Island, maritime Antarctica), these soil contents may have their origin in supraglacial meltwater, ancient subglacial sediment carbon, or aeolian deposition. Moreover, the increased abundance of aquatic lineages in *Chytridiomycota* and *Rozellomycota* in soils over rocks could be favored be occasional flooding of the glacier forefield as ice melts (Shearer et al., 2007; Lara et al., 2010). In contrast, the *Lecanoromycetes* were clearly more abundant on rocks than soil and their abundance increased over time. The higher abundances of the algal genera *Prasiola* (*Prasiolales*) and *Trebouxia* (*Microthamniales*) in the lithic substrate indicate potential lichen symbiotic associations with *Lecanoromycetes* (de los Ríos et al., 2014; Garrido-Benavent et al., 2017). Together with mosses, lichens are the most conspicuous biotic element of the Antarctic terrestrial landscapes (Peat et al., 2007), and in particular, the number of lichens of Hurd Peninsula in Livingston Island well exceeds 200 taxa, many of which grow preferentially, and profusely, on rocks (Søchting et al., 2004). Lichen associations have been indeed revealed by electron microscopy analyses occupying endolithic microhabitats after 23 years of glacial retreat although lichen thalli themselves were not observed colonizing the rock surface at the analyzed chronosequence. Finally, our data recapitulate previous findings of the algal order *Sphaeropleales* being a typical component of the soil algal biota (Broady, 1984; Fučíková et al., 2014), occasionally favored by flooding (Sciuto et al., 2015), as well as the ulothricacean algae being rock colonizers (Schmidt and Darcy, 2015).

Succession patterns of bacterial and fungal communities in moraine rocks and soils differed along the entire chronosequence. Our ordination results revealed a minimal community overlap among succession stages along the soil chronosequence, whereas the communities of rocks at the second stage (∼13 years since glacial retreat) were intermixed with either the first or third stage, or both. Lithobiontic bacterial and fungal communities were more variable in their intra-OTU composition than the edaphic communities as well. Altogether, these findings indicate that the drivers of microbial community assembly in these niches could be either distinct and/or operate at different spatial and temporal scales. Our analyses of electron microscopy showing a patchy distribution of the epilithic and endolithic colonization and substantial colonization only after 13 years of exposure point toward this direction. The bioreceptivity of rocks to endolithic colonization is markedly influenced by physical and chemical rock properties (Guillitte, 1995), of which texture, or porosity, may be crucial (Cámara et al., 2014). Indeed, the different rock microhabitats host different microbial communities (de los Ríos et al., 2014; Yung et al., 2014). Abiotic and biotic interactions occurring in rock microhabitats evolve with time toward specific microbial microniches (Pointing and Belnap, 2012), which could facilitate the establishment of more specialized microbial communities. Hence, the initial assembling and subsequent successional changes in community structure in the lithic niche are more controlled by the time being ice-free than in the spatially more homogenous soils.

A high diversity in terms of the number of ASVs, compared to that of OTUs, was revealed for all three groups of organisms which is intrinsically related to the nature of the DADA2 algorithm, which preserves granular biological information that is likely excluded via the use of clustering algorithms (Callahan et al., 2017). Taxonomic assignments and community structure were consistent across the two approaches (OTUs and ASVs). However, we found contrasting numbers of shared OTUs and ASVs between the edaphic and lithic niches, which suggests that in spite of the gross sharing of bacterial, algal and to a lesser extent fungal OTUs, these niches, independently, host a greater proportion of unique genetic diversity. Several processes, such as selection, dispersal, speciation, or drift may be at the basis of our finding (Vellend, 2010; Nemergut et al., 2013). In particular, ecological speciation predicts that populations adapted to contrasting environments would undergo reproductive isolation as a result of ecologically-based divergent selection (Rundle and Nosil, 2005; Schluter, 2009). The lithic and edaphic substrates are in fact different in several aspects, the most obvious related to microenvironmental factors, which exert strong and different selection pressures in each substrate (de los Ríos et al., 2003, 2007; Chan et al., 2013; Wei et al., 2016). Evidences for ecological speciation in microorganisms are being gradually compiled. In the Atacama Desert, Meslier et al. (2018) have recently revealed a high specificity of endolithic bacterial communities to physico-chemically different rocks. In Antarctica, a number of studies have uncovered an unprecedented genetic and taxonomic diversity of fungi, algae and cyanobacteria colonizing rocks, even in the most climatically extreme habitats (Selbmann et al., 2005; de los Ríos et al., 2014; Yung et al., 2014).

Combined with selection, the stochastic process of dispersal (Nemergut et al., 2013) may contribute to the observed differences in microbial diversity between substrates along the succession (Makoto and Wilson, 2019). In our studied glacier forefield, dispersal is likely driven either by wind or glacial melt water (Kappen and Straka, 1988; Marshall, 1996a, b; Šabacká et al., 2012), although the abundant fauna in the area may have an equally significant impact. Selection during transit and at sink positions may ultimately depend on the microbial group (Wilkinson et al., 2012; Archer et al., 2019), and substrate geomorphology (Pointing and Belnap, 2012). Thus, it is expected that more constraints to dispersal exist for large lichen propagula, such as soredia and isidia, or algal cysts, than for minuscule bacterial cells. Limitations to fungal dispersal in terrestrial habitats seem indeed relevant in other Antarctic regions (Archer et al., 2019). The patchy distribution of rocks in the studied area and the spatially heterogeneous distribution of rock microhabitats likely exert a stronger filter on the ability of air- and water-borne microorganisms to colonize the lithic substrate compared to the edaphic substrate. These differences in spatial distribution of potential microhabitats determine subsequent community dynamics as suggested by the higher β-diversity (β_*SOR*_) for lithobiontic than edaphic bacterial and fungal communities in spite of the generally lower α-diversity indices of lithic communities.

Beyond the persistence of community differentiation between substrates along the chronosequence, our study demonstrated that lithic and edaphic microbial communities changed over time, and that community composition changes were steeper for bacteria and fungi than algae. These changes were concomitant with a general increase of alpha-diversity estimators and, in soils, with the decrease in pH and increases in soil nutrient availability and, specially, organic matter and C content. In general, these results concur with those of previous works studying soil chronosequences in retreating glaciers (Hodkinson et al., 2003; Schütte et al., 2010; Brown and Jumpponen, 2014; Kazemi et al., 2016; Fernández-Martínez et al., 2017; Jiang et al., 2018), and therefore support exposure time as a reliable predictor of microbial succession in polar regions as well. We additionally showed that the relative contribution of the two components of β-diversity (turnover and nestedness, *sensu* Baselga and Orme, 2012) to community variation for the three organismal groups was different in the two niches. Most interestingly, our results indicated that taxa replacement and loss contributed equally to variation in fungal lithic and bacterial edaphic communities. However, recently deglaciated rocks hosted a subset of the bacteria found in rocks being ice-free for longer (nested structure), and edaphic community variation of fungi and algae was basically dominated by taxa replacement (turnover) along the chronosequence. However, diving further into the mechanics of community variation for each studied organismal group is out of the scope of the present work. Finally, our study demonstrated that significant changes in microbial community composition may occur in polar latitudes within shorter temporal intervals (∼23 years chronosequence, as opposed to >50 years in the studies listed above) and without the involvement of vascular plants. In line with our findings, Nemergut et al. (2007) revealed dramatic shifts in bacterial community composition in a ∼20-year chronosequence of three unvegetated soils from a receding glacier in southeastern Peru. A similar pattern was found for fungi in the pioneering study of Jumpponen (2003) in a forefront devoid of plants in the Lyman Glacier (Washington, United States), but this study indicated a decrease in fungal diversity along succession stages. Each study, however, used different methods, preventing in-depth comparisons with ours.

## Conclusion

In conclusion, as we anticipated, the edaphic and lithic niches of this maritime Antarctic glacier forefield hosted bacterial and fungal communities showing contrasting succession patterns. However, our hypothesis was refuted in part as community structural differences were present since the start of succession (within a year of deglaciation). These findings suggest a critical window for the microbial colonization of exposed surfaces. This is important for understanding biological turnover and predicting the response of microbial communities to climate change in Antarctic systems.

## Data Availability Statement

The datasets generated for this study can be found in the NCBI Sequence Read Archive with the following BioProject numbers: bacteria (soils: PRJNA592870; rocks: PRJNA592961), fungi (soils: PRJNA592910; rocks: PRJNA592919), and algae (soils: PRJNA592949; rocks: PRJNA592957), as well as in the Supplementary Material.

## Author Contributions

IG-B carried out the data analyses and wrote the manuscript. JD co-analyzed the data of soil attributes. RR-C and FN provided crucial data on the ages of rock and soils in the Antarctic chronosequence. CA and SP contributed to the manuscript edition. AR, SP-O, and JD designed the study and the sampling strategy, participated in the fieldwork, and contributed to improve the manuscript edition.

## Conflict of Interest

The authors declare that the research was conducted in the absence of any commercial or financial relationships that could be construed as a potential conflict of interest.

## Footnotes

1 https://github.com/mlangill/microbiome_helper/wiki/16S-standard-operating-procedure

2 https://github.com/mlangill/microbiome_helper/wiki

3 https://github.com/darcyj

4 http://cgenome.net/wiki/index.php/Calypso
