# Supplementary material for: Differential Colonization and Succession of Microbial Communities in Rock and Soil Substrates on a Maritime Antarctic Glacier Forefield
Source: Front Microbiol. 2020 Feb 7;11:126. doi: 10.3389/fmicb.2020.00126 (PMC7018881; doi:10.3389/fmicb.2020.00126)
Supplement: Supplementary file 1 [file Data_Sheet_1.pdf]

Supplementary Material

Differential colonization and succession of microbial communities in rock and soil substrates on a maritime Antarctic glacier forefield

Isaac Garrido-Benavent, Sergio Pérez-Ortega, Jorge Durán, Carmen Ascaso, Stephen B. Pointing, Ricardo Rodríguez-Cielos, Francisco Navarro and Asunción de los Ríos

*** Correspondence:**
Isaac Garrido-Benavent
igbenavent@mncn.csic.es

This document includes the commands used to construct OTU and ASV tables (in BIOM format) for bacteria, fungi and algae. The analyses were conducted in a Mac OS X El Capitán with 8 Gb Ram. I installed the Microbiome Helper virtual box v. 0.3 (Comeau et al., 2017) which is based on QIIME1 (Caporaso et al., 2010) and that includes also dada2 scripts (Callahan et al., 2016). (**pages 1-48**)

Additionally, it includes the R scripts used to analyse the soil abiotic variables separately, and combined with the biological abundance data (**pages 49-69**) .

I) INFERENCE OF 97% Operational Taxonomic Units (OTUs)

Ia) Operating procedure for analyzing bacterial 16S data to infer 97% OTUs using the Microbiome Helper virtual box (Comeau et al., 2017; https://github.com/mlangill/microbiome_helper/wiki/16S-standard-operating-180 procedure) with some custom modifications. **(page 2)**

Ib) Operating procedure for analyzing fungal *ITS1* data to infer 97% OTUs using PIPITS v. 1.5.0 (Gweon et al., 2015) and QIIME1 as implemented in the Microbiome Helper virtual box (Comeau et al., 2017; https://github.com/mlangill/microbiome_helper/wiki/16S-standard-operating-180 procedure) with some custom modifications. **(page 11)**

Ic) Operating procedure for analyzing fungal *rbcL* data to infer 97% OTUs using the Microbiome Helper virtual box (Comeau et al., 2017; https://github.com/mlangill/microbiome_helper/wiki/16S-standard-operating-180 procedure), with some custom modifications. **(page 21)**

II) INFERENCE OF Amplicon Sequence Variants (ASVs)

IIa) Operating procedure for analyzing bacterial 16S data to infer ASVs using the Microbiome Helper virtual box (Comeau et al., 2017; https://github.com/mlangill/microbiome_helper/wiki/16S-standard-operating-180 procedure) and dada2 (Callahan et al., 2016) scripts, with some custom modifications. **(page 32)**

IIb) Operating procedure for analyzing fungal *ITS1* data to infer ASVs using Microbiome Helper virtual box (Comeau et al., 2017) and dada2 (Callahan et al., 2016) scripts, with some custom modifications. **(page 37)**

IIc) Operating procedure for analyzing fungal *rbcL* data to infer ASVs using the Microbiome Helper virtual box (Comeau et al., 2017; https://github.com/mlangill/microbiome_helper/wiki/16S-standard-operating-180 procedure) and dada2 (Callahan et al., 2016) scripts, with some custom modifications. **(page 44)**

Ia) Operating procedure for analyzing bacterial 16S data to infer 97% OTUs using the Microbiome Helper virtual box (Comeau et al., 2017; https://github.com/mlangill/microbiome_helper/wiki/16S-standard-operating-180 procedure) with some custom modifications

Date: 26-APR-2018; Author: Isaac Garrido Benavent; Software: MV Microbiome Helper amplicon v 0.3

Primer used for amplification of the V1-V2 region within the 16AS:

27F: AGAGTTTGATCMTGGCTCAG and 338R: CATGCTGCCTCCCGTAGGAGT

#########

source /home/mh_user/anaconda2/bin/activate qiime1 #activating Qiime1 within Microbiome Helper virtual box

#########<u>Check paired-end data and order them according to sample name</u>

cd ~/Desktop/clus_97_bacteria

mkdir 1_bac_sorted_fastqs

parallel -j 20 'cat {} | paste - - - - | sort -k1,1 -t " " | tr "\t" "\n" > 1_bac_sorted_fastqs/{/.}.sorted.fastq' ::: rawdata/*.fastq

#########<u>Stitching paired-end reads</u>

run_pear.pl -p 3 -o 2_stitched_reads 1_bac_sorted_fastqs/*fastq ## ("-p 3" indicates this job should be run on 3 CPU and "-o 2_stitched_reads" indicates the output folder)

##########<u>Obtaining quality metrics of stitched reads with FASTQC</u>

mkdir 3_fastqc_out_combined

cat 2_stitched_reads/*.assembled.fastq | fastqc -t 1 stdin -o 3_fastqc_out_combined

cd 3_fastqc_out_combined ##after this step, files must be renamed

mv stdin_fastqc.html combined_fastqc.html

mv stdin_fastqc.zip combined_fastqc.zip

###########<u>Filtering reads</u>

cd ~/Desktop/clus_97_bacteria

read_filter.pl -f AGAGTTTGATCMTGGCTCAG -r ACTCCTACGGGAGGCAGCATG -q 28 -p 90 -l 280 -thread 3 -o 4_filtered_reads -c both -t 2_stitched_reads/*.assembled*fastq

#########<u>Conversion to FASTA</u>

run_fastq_to_fasta.pl -p 2 -o 5_fasta_files 4_filtered_reads/*fastq #this command also eliminates sequences with any "N"

#########Removal of chimeric reads using two consecutive strategies: *de novo*, and reference-based

1) *De novo* chimaera removal

mkdir 6_fasta_files_derep

mkdir 7_non_denovo_chimeras

mkdir 8_non_denovo_chimeras_rerep

parallel -j 2 'vsearch --derep_fulllength {} --sizeout --output 6_fasta_files_derep/{/.}.derep.fasta' ::: 5_fasta_files/*fasta

parallel --eta -j 2 'vsearch --uchime_denovo {} --nonchimeras 7_non_denovo_chimeras/{/.}.nonchimera.fasta 2> 7_non_denovo_chimeras/{/.}.nonchimera.log' ::: 6_fasta_files_derep/*.derep.fasta

parallel -j 2 'vsearch --rereplicate {} --output 8_non_denovo_chimeras_rerep/{/.}.rerep.fasta' ::: 7_non_denovo_chimeras/*fasta

#2) Chimaera removal based on a reference database (here I use a 2016 version of the RDP dataset which also includes Archaea)

chimera_filter.pl -type 1 -thread 3 -db /home/shared/rRNA_db/RDP_trainset16_022016.fa 8_non_denovo_chimeras_rerep/*fasta

#########Formatting the input Fasta files and generate a single Fasta file with adequate labels (before this, eliminate any hyphen in file names, and replace it with an underscore. Prepare also a mapping file, see webpage of Microbiome Helper virtual box)

add_qiime_labels.py -i 9_non_chimeras/ -m mapbac.txt -c FileInput -o 10_combined_fasta

##########Remove any further sequence artifact (e.gg extremely long sequence reads) using seqkit (Shen et al. 2016).

sudo conda install -c bioconda seqkit #this command installs seqkit

#Generate some sequence statistics (e.g. length in bp) before and after trimming with seqkit.

cd ~/Desktop/clus_97_bacteria

count_seqs.py -i 10_combined_fasta/combined_seqs.fna -o 10_combined_fasta/1_stat_combined_seqs.txt

```
cd 10_combined_fasta

cat combined_seqs.fna | seqkit seq | seqkit stats >> 2_seq_stats_previous_trimming.txt

cat combined_seqs.fna | seqkit seq -m 250 -M 320 | seqkit stats >> 3_seq_stats_after_trimming.txt

seqkit seq -m 250 -M 320 combined_seqs.fna > 4_lengthfiltered_combined_seqs.fna

cd ..

count_seqs.py -i 10_combined_fasta/4_lengthfiltered_combined_seqs.fna -o 10_combined_fasta/5_after_seqkit_stat_combined_seqs.txt
```

########Run open-reference OTU picking pipeline: using SORTMERNA and the most up-to-date version of SILVA database

```
mkdir ~/Desktop/clus_97_bacteria/11_clustering

cd ~/Desktop/clus_97_bacteria/11_clustering

echo "pick_otus:threads 3" >> clustering_params_silva.txt

echo "pick_otus:enable_rev_strand_match True" >> clustering_params_silva.txt

echo "pick_otus:sortmerna_coverage 0.8" >> clustering_params_silva.txt

echo "pick_otus:similarity 0.97" >> clustering_params_silva.txt

echo "align_seqs:template_fp /home/shared/rRNA_db/SILVA_128_QIIME_release/core_alignment/core_alignment_SILVA128.fna" >> clustering_params_silva.txt

echo "filter_alignment:allowed_gap_frac 0.80" >> clustering_params_silva.txt

echo "filter_alignment:entropy_threshold 0.10" >> clustering_params_silva.txt

echo "filter_alignment:suppress_lane_mask_filter True" >> clustering_params_silva.txt

echo "assign_taxonomy:reference_seqs_fp /home/shared/rRNA_db/SILVA_128_QIIME_release/rep_set/rep_set_all/97/97_otus.fasta" >> clustering_params_silva.txt

echo "assign_taxonomy:id_to_taxonomy_fp /home/shared/rRNA_db/SILVA_128_QIIME_release/taxonomy/taxonomy_all/97/taxonomy_7_levels.txt" >> clustering_params_silva.txt

pick_open_reference_otus.py -i /home/mh_user/Desktop/clus_97_bacteria/10_combined_fasta/4_lengthfiltered_combined_seqs.fna -o /home/mh_user/Desktop/clus_97_bacteria/11_clustering/ -p /home/mh_user/Desktop/clus_97_bacteria/clus_97_bacteria/clustering_params_silva.txt -m sortmerna_sumaclust -r
```

/home/shared/rRNA_db/SILVA_128_QIIME_release/rep_set/rep_set_16S_only/97/97_otus_16S.fasta -s 0.1 -v --min_otu_size 1

##########Remove low confidence OTUs

remove_low_confidence_otus.py -i /home/mh_user/Desktop/clus_97_bacteria/11_clustering/otu_table_mc1_w_tax_no_pynast_failures.biom -o /home/mh_user/Desktop/clus_97_bacteria/11_clustering/otu_table_high_conf.biom

##########Comparing the results of the two BIOM files

cd ~/Desktop/clus_97_bacteria

biom summarize-table -i 11_clustering/otu_table_mc1_w_tax_no_pynast_failures.biom -o 11_clustering/otu_table_mc1_w_tax_no_pynast_failures_summary.txt

biom summarize-table -i 11_clustering/otu_table_high_conf.biom -o 11_clustering/otu_table_high_conf_summary.txt

###########Inspecting the final OTU table that includes taxonomic labels

biom convert -i /home/mh_user/Desktop/clus_97_bacteria/11_clustering/otu_table_high_conf.biom -o /home/mh_user/Desktop/clus_97_bacteria/11_clustering/otu_table_definitiva.txt --to-tsv --header-key taxonomy

###########Remove OTUs corresponding labelled as “Chloroplast” and “Mitochondria”

filter_taxa_from_otu_table.py -i /home/mh_user/Desktop/clus_97_bacteria/11_clustering/otu_table_high_conf.biom -o /home/mh_user/Desktop/clus_97_bacteria/11_clustering/otu_table_definitiva2_wout_mito_chloro.biom -n D_2__Chloroplast,D_4__Mitochondria

###########Check again

biom convert -i /home/mh_user/Desktop/clus_97_bacteria/11_clustering/otu_table_definitiva2_wout_mito_chloro.biom -o /home/mh_user/Desktop/clus_97_bacteria/11_clustering/otu_table_definitiva2_wout_mito_chloro.txt --to-tsv --header-key taxonomy

############Order samples within the BIOM table according to a user-specific criterion, Summarize and Plot taxonomy:

cd ~/Desktop/clus_97_bacteria

sort_otu_table.py -i /home/mh_user/Desktop/clus_97_bacteria/11_clustering/otu_table_definitiva2_wout_mito_chloro.biom -o

/home/mh_user/Desktop/clus_97_bacteria/11_clustering/otu_table_definitiva2_wout_mito_chloro_sorted.biom -l /home/mh_user/Desktop/clus_97_bacteria/11_clustering/sample_id_list.txt

mkdir 12_sum_plot_tax

summarize_taxa.py -i /home/mh_user/Desktop/clus_97_bacteria/11_clustering/otu_table_definitiva2_wout_mito_chloro_sorted.biom -L 2 -o /home/mh_user/Desktop/clus_97_bacteria/12_sum_plot_tax/1_phylum/ #

summarize_taxa.py -i /home/mh_user/Desktop/clus_97_bacteria/11_clustering/otu_table_definitiva2_wout_mito_chloro_sorted.biom -L 3 -o /home/mh_user/Desktop/clus_97_bacteria/12_sum_plot_tax/2_class/

summarize_taxa.py -i /home/mh_user/Desktop/clus_97_bacteria/11_clustering/otu_table_definitiva2_wout_mito_chloro_sorted.biom -L 4 -o /home/mh_user/Desktop/clus_97_bacteria/12_sum_plot_tax/3_order/

plot_taxa_summary.py -i /home/mh_user/Desktop/clus_97_bacteria/12_sum_plot_tax/1_phylum/otu_table_definitiva2_wout_mito_chloro_sorted_L2.txt -l phylum -c pie,bar,area -o 1_phylum_charts/

plot_taxa_summary.py -i /home/mh_user/Desktop/clus_97_bacteria/12_sum_plot_tax/2_class/otu_table_definitiva2_wout_mito_chloro_sorted_L3.txt -l class -c pie,bar,area -o 2_class_charts/

plot_taxa_summary.py -i /home/mh_user/Desktop/clus_97_bacteria/12_sum_plot_tax/3_order/otu_table_definitiva2_wout_mito_chloro_sorted_L4.txt -l order -c pie,bar,area -o 3_order_charts/

##############Rarify reads prior to estimating alpha-diversity statistics

cd ~/Desktop/clus_97_bacteria

mkdir 13_final_otu_tables_rarefaction

single_rarefaction.py -i 11_clustering/otu_table_definitiva2_wout_mito_chloro_sorted.biom -o 13_final_otu_tables_rarefaction/otu_table_rarified.biom -d 38530

##############Diversity analyses (1): alpha-diversity (with and without rarefaction)

cd ~/Desktop/clus_97_bacteria

mkdir 14_alpha_diversity

alpha_diversity.py -i 11_clustering/otu_table_definitiva2_wout_mito_chloro.biom -m ace,chao1,observed_otus,goods_coverage,shannon,simpson -o 14_alpha_diversity/alpha_diversity_metrics_not_rarif.txt

alpha_diversity.py -i 13_final_otu_tables_rarefaction/otu_table_rarified.biom -m ace,chao1,observed_otus,goods_coverage,shannon,simpson -o 14_alpha_diversity/alpha_diversity_metrics_rarif.txt

#Rarefaction curves for each metric:

cd ~/Desktop/clus_97_bacteria/14_alpha_diversity

echo 'alpha_diversity:metrics observed_species,shannon,chao1' > parameters_alphaDiver.txt

cd ~/Desktop/clus_97_bacteria/

alpha_rarefaction.py -i 13_final_otu_tables_rarefaction/otu_table_rarified.biom -o 14_alpha_diversity/rarefaction_curv/ -p 14_alpha_diversity/parameters_alphaDiver.txt -m /home/mh_user/Desktop/clus_97_bacteria/mapbac.txt

#############Adding metadata to BIOM file to conduct beta-diversity analyses. This step needs first to construct a txt file with samples and metadata (here named “mapbac.txt”)

cd ~/Desktop/clus_97_bacteria

biom add-metadata -i /home/mh_user/Desktop/clus_97_bacteria/11_clustering/otu_table_definitiva2_wout_mito_chloro_sorted.biom -o /home/mh_user/Desktop/clus_97_bacteria/11_clustering/table_BIOM_metadata.biom --sample-metadata-fp /home/mh_user/Desktop/clus_97_bacteria/mapbac.txt

#############Normalize OTU table by CSS method

cd ~/Desktop/clus_97_bacteria

mkdir 15_normalized_BIOM_table

normalize_table.py -i /home/mh_user/Desktop/clus_97_bacteria/11_clustering/table_BIOM_metadata.biom -a CSS -o 15_normalized_BIOM_table/CSS_normalized_otu_table.biom

##############Beta-diversity and PCoA

cd ~/Desktop/clus_97_bacteria

mkdir 16_bdiversity

beta_diversity.py -i /home/mh_user/Desktop/clus_97_bacteria/15_normalized_BIOM_table/CSS_normalized_otu_table.biom -o /home/mh_user/Desktop/clus_97_bacteria/16_bdiversity/bdiv_BrayCurtis/ -m bray_curtis

```
principal_coordinates.py -i
/home/mh_user/Desktop/clus_97_bacteria/16_bdiversity/bdiv_BrayCurtis/bray_curtis_CSS_normalized_otu_table.txt -o
/home/mh_user/Desktop/clus_97_bacteria/16_bdiversity/bdiv_BrayCurtis/bdiv_BrayCurt_coords.txt

make_2d_plots.py -i
/home/mh_user/Desktop/clus_97_bacteria/16_bdiversity/bdiv_BrayCurtis/bdiv_BrayCurt_coords.txt -m /home/mh_user/Desktop/clus_97_bacteria/mapbac.txt -o
/home/mh_user/Desktop/clus_97_bacteria/16_bdiversity/bdiv_BrayCurtis/bdiv_BrayCurtis/
```

#############Comparing Categories. Basic Question: Do different sample groupings significantly differ in their microbial composition?

```
cd ~/Desktop/clus_97_bacteria

mkdir 17_bdiver_statistical_tests

compare_categories.py --method anosim -i
/home/mh_user/Desktop/clus_97_bacteria/16_bdiversity/bdiv_BrayCurtis/bray_curtis_CSS_normalized_otu_table.txt -m
/home/mh_user/Desktop/clus_97_bacteria/mapbac.txt -c Source -o
/home/mh_user/Desktop/clus_97_bacteria/17_bdiver_statistical_tests/1_overall_source/anosim_out

compare_categories.py --method adonis -i
/home/mh_user/Desktop/clus_97_bacteria/16_bdiversity/bdiv_BrayCurtis/bray_curtis_CSS_normalized_otu_table.txt -m
/home/mh_user/Desktop/clus_97_bacteria/mapbac.txt -c Source -o
/home/mh_user/Desktop/clus_97_bacteria/17_bdiver_statistical_tests/1_overall_source/adonis_out
```

#A)Splitting BIOM table according to metadata file and category

```
cd ~/Desktop/clus_97_bacteria/17_bdiver_statistical_tests

mkdir 2_bdiv_anal_per_category

cd ..

filter_samples_from_otu_table.py -i
/home/mh_user/Desktop/clus_97_bacteria/11_clustering/otu_table_definitiva2_wout_mito_chloro_sorted.biom -o
/home/mh_user/Desktop/clus_97_bacteria/17_bdiver_statistical_tests/2_bdiv_anal_per_category/otu_table_soil.biom -m
/home/mh_user/Desktop/clus_97_bacteria/mapbac.txt -s 'Source:Soil'

filter_samples_from_otu_table.py -i
/home/mh_user/Desktop/clus_97_bacteria/11_clustering/otu_table_definitiva2_wout_mito_chloro_sorted.biom -o
/home/mh_user/Desktop/clus_97_bacteria/17_bdiver_statistical_tests/2_bdiv_anal_per_
```

category/otu_table_rock.biom -m /home/mh_user/Desktop/clus_97_bacteria/mapbac.txt -s 'Source:Rock'

#B)Manually generate two mapping files ("mapbacsoil.txt" y "mapbacrock.txt") for adding metadata to the two BIOM files obtained in the previous step. Save these mapping files in folder "2_bdiv_anal_per_category"

cd ~/Desktop/clus_97_bacteria/17_bdiver_statistical_tests/2_bdiv_anal_per_category

biom add-metadata -i otu_table_soil.biom -o otu_table_soil_metadata.biom --sample-metadata-fp /home/mh_user/Desktop/clus_97_bacteria/17_bdiver_statistical_tests/2_bdiv_anal_per_category/mapbacsoil.txt

biom add-metadata -i otu_table_rock.biom -o otu_table_rock_metadata.biom --sample-metadata-fp /home/mh_user/Desktop/clus_97_bacteria/17_bdiver_statistical_tests/2_bdiv_anal_per_category/mapbacrock.txt

#C)Normalize BIOM tables with CSS method

cd ~/Desktop/clus_97_bacteria/17_bdiver_statistical_tests/2_bdiv_anal_per_category

normalize_table.py -i otu_table_soil_metadata.biom -a CSS -o CSS_norm_otu_table_soil.biom

normalize_table.py -i otu_table_rock_metadata.biom -a CSS -o CSS_norm_otu_table_rock.biom

#######analyses of soil data#######

cd ~/Desktop/clus_97_bacteria/17_bdiver_statistical_tests/2_bdiv_anal_per_category

beta_diversity.py -i CSS_norm_otu_table_soil.biom -o bdiv_soil_BrayCurtis/ -m bray_curtis #hago la matriz de disimilitud de Bray-Curtis

principal_coordinates.py -i ./bdiv_soil_BrayCurtis/bray_curtis_CSS_norm_otu_table_soil.txt -o ./bdiv_soil_BrayCurtis/bdiv_soil_BrayCurt_coords.txt

make_2d_plots.py -i ./bdiv_soil_BrayCurtis/bdiv_soil_BrayCurt_coords.txt -m mapbacsoil.txt -o ./bdiv_soil_BrayCurtis/ #this graphics the PCoA 2d plot

#######analyses of rock data#######

beta_diversity.py -i CSS_norm_otu_table_rock.biom -o bdiv_rock_BrayCurtis/ -m bray_curtis

principal_coordinates.py -i
./bdiv_rock_BrayCurtis/bray_curtis_CSS_norm_otu_table_rock.txt -o
./bdiv_rock_BrayCurtis/bdiv_rock_BrayCurt_coords.txt

make_2d_plots.py -i ./bdiv_rock_BrayCurtis/bdiv_rock_BrayCurt_coords.txt -m
mapbacrock.txt -o ./bdiv_rock_BrayCurtis/ #this graphics the PCoA 2d plot

#D)<u>Run ADONIS y ANOSIM to test whether the sample groupings revealed in the PCoA with respect to time are supported or not</u>

mkdir bdiver_stat_tests_per_category

#######analyses of soil data#######

compare_categories.py --method anosim -i
~/Desktop/clus_97_bacteria/17_bdiver_statistical_tests/2_bdiv_anal_per_category/bdiv_soil_BrayCurtis/bray_curtis_CSS_norm_otu_table_soil.txt -m
~/Desktop/clus_97_bacteria/17_bdiver_statistical_tests/2_bdiv_anal_per_category/mapbacsoil.txt -c Time -o
~/Desktop/clus_97_bacteria/17_bdiver_statistical_tests/2_bdiv_anal_per_category/bdiver_stat_tests_per_category/anosim_out_soil

compare_categories.py --method adonis -i
~/Desktop/clus_97_bacteria/17_bdiver_statistical_tests/2_bdiv_anal_per_category/bdiv_soil_BrayCurtis/bray_curtis_CSS_norm_otu_table_soil.txt -m
~/Desktop/clus_97_bacteria/17_bdiver_statistical_tests/2_bdiv_anal_per_category/mapbacsoil.txt -c Time -o
~/Desktop/clus_97_bacteria/17_bdiver_statistical_tests/2_bdiv_anal_per_category/bdiver_stat_tests_per_category/adonis_out_soil

#######analyses of rock data#######

compare_categories.py --method anosim -i
~/Desktop/clus_97_bacteria/17_bdiver_statistical_tests/2_bdiv_anal_per_category/bdiv_rock_BrayCurtis/bray_curtis_CSS_norm_otu_table_rock.txt -m
~/Desktop/clus_97_bacteria/17_bdiver_statistical_tests/2_bdiv_anal_per_category/mapbacrock.txt -c Time -o
~/Desktop/clus_97_bacteria/17_bdiver_statistical_tests/2_bdiv_anal_per_category/bdiver_stat_tests_per_category/anosim_out_rock

compare_categories.py --method adonis -i
~/Desktop/clus_97_bacteria/17_bdiver_statistical_tests/2_bdiv_anal_per_category/bdiv_rock_BrayCurtis/bray_curtis_CSS_norm_otu_table_rock.txt -m
~/Desktop/clus_97_bacteria/17_bdiver_statistical_tests/2_bdiv_anal_per_category/mapbacrock.txt -c Time -o
~/Desktop/clus_97_bacteria/17_bdiver_statistical_tests/2_bdiv_anal_per_category/bdiver_stat_tests_per_category/adonis_out_rock

##

Ib) Operating procedure for analyzing fungal *ITS1* data to infer 97% OTUs using PIPITS v. 1.5.0 (Gweon et al., 2015) and QIIME1 as implemented in the Microbiome Helper virtual box (Comeau et al., 2017; https://github.com/mlangill/microbiome_helper/wiki/16S-standard-operating-180 procedure) with some custom modifications.

Date: 16-FEB-2018; Author: Isaac Garrido Benavent; Software: MV Microbiome Helper amplicon v 0.3 and PIPITS v.1.5

Primer used for amplification of the ITS region within the rDNA:

ITS1F_KYO2_CS1: ACACTGACGACATGGTTCTACATAGAGGAAGTAAAAGTCGTAA and ITS2_KYO2_CS2: TACGGTAGCAGAGACTTGGTCTTTYRCTRCGTTCTTCATC

Database used for taxonomic assignment: UNITE v.28.06.2017

############

############PIPITS data processing

cd $HOME/pipits-1.5.0/fungi

pipits_getreadpairslist -i rawdata -o readpairslist.txt

pipits_prep -i rawdata -o pipits_prep -l readpairslist.txt

pipits_funits -i pipits_prep/prepped.fasta -o pipits_funits -x ITS1

pipits_process -i pipits_funits/ITS.fasta -o out_process -t 3 --Xmx 3G # -t 3 = uses 3 threads

#################Editing of the BIOM file obtained with PIPITS

########<u>Remove undesired OTUs (e.g. Mitocondria, Cercozoa, Viridiplante, and so on)</u>

source /home/mh_user/anaconda2/bin/activate qiime1

filter_taxa_from_otu_table.py -i $HOME/pipits-1.5.0/fungi/out_process/otu_table.biom -o $HOME/pipits-1.5.0/fungi/out_process/otu_table_wout_unwant_OTUs.biom -n k__Animalia,k__Chromista,k__Protista,k__Plantae

biom convert -i $HOME/pipits-1.5.0/fungi/out_process/otu_table_wout_unwant_OTUs.biom -o $HOME/pipits-1.5.0/fungi/out_process/otu_table_wout_unwant_OTUs.txt --to-tsv --header-key

taxonomy ##this transforms the BIOM into a txt file in which is more easy to check that the undesired OTUs have been already removed

########Remove from the FASTA file the sequences corresponding to these undesired OTUs

filter_fasta.py -f $HOME/pipits-1.5.0/fungi/out_process/repseqs.fasta -b $HOME/pipits-1.5.0/fungi/out_process/otu_table_wout_unwant_OTUs.biom -o $HOME/pipits-1.5.0/fungi/out_process/repseq_filt_unwant_OTUs.fna

########Then, open the OTU table in txt format named "otu_table_wout_unwant_OTUs.txt" in Microsoft Excel and conduct the following steps (#1-#13):

#1) First, create two txt files with the OTU ID of those OTUs whose taxonomic assignment may be improved such as (1) the "Unassignable", and those with incomplete taxonomic assignments, such as (2) "k__Fungi". Thus, one txt file would be named "OTU_IDs_unassign.txt" and the other "OTU_IDs_incompl_tax.txt".

#2) In Microbiome Helper, and with these two txt files, execute the following commands on the BIOM file:

filter_otus_from_otu_table.py -i $HOME/pipits-1.5.0/fungi/out_process/otu_table_wout_unwant_OTUs.biom -o $HOME/pipits-1.5.0/fungi/out_process/otu_table_unassigned.biom -e $HOME/pipits-1.5.0/fungi/out_process/OTU_IDs_unassign.txt --negate_ids_to_exclude

filter_otus_from_otu_table.py -i $HOME/pipits-1.5.0/fungi/out_process/otu_table_wout_unwant_OTUs.biom -o $HOME/pipits-1.5.0/fungi/out_process/otu_table_incompl_taxonomy.biom -e $HOME/pipits-1.5.0/fungi/out_process/OTU_IDs_incompl_tax.txt --negate_ids_to_exclude

#3) Then, use the two new BIOM files to extract the selected sequences from the FASTA file:

filter_fasta.py -f $HOME/pipits-1.5.0/fungi/out_process/repseq_filt_unwant_OTUs.fna -b $HOME/pipits-1.5.0/fungi/out_process/otu_table_unassigned.biom -o $HOME/pipits-1.5.0/fungi/out_process/repseq_unassigned.fna

filter_fasta.py -f $HOME/pipits-1.5.0/fungi/out_process/repseq_filt_unwant_OTUs.fna -b $HOME/pipits-1.5.0/fungi/out_process/otu_table_incompl_taxonomy.biom -o $HOME/pipits-1.5.0/fungi/out_process/repseq_incompl_taxonomy.fna

#4) Reassign taxonomy with SortMerna to these two sequence files based on a custom database of ITS sequences generated from GenBank data:

assign_taxonomy.py -i $HOME/pipits-1.5.0/fungi/out_process/repseq_unassigned.fna -t /home/mh_user/Desktop/Custom_databases/qiime_database_fung_run-home/Taxonomy_final.txt -r /home/mh_user/Desktop/Custom_databases/qiime_database_fung_run-home/DB.fasta -m sortmerna --sortmerna_threads 3 -o $HOME/pipits-1.5.0/fungi/out_process/unassigned/

assign_taxonomy.py -i $HOME/pipits-1.5.0/fungi/out_process/repseq_incompl_taxonomy.fna -t /home/mh_user/Desktop/Custom_databases/qiime_database_fung_run-home/Taxonomy_final.txt -r /home/mh_user/Desktop/Custom_databases/qiime_database_fung_run-home/DB.fasta -m sortmerna --sortmerna_threads 3 -o $HOME/pipits-1.5.0/fungi/out_process/incomplete_tax/

#5) Manually, in Excel, copy the Tax IDs of the OTUs whose taxonomic assignments have improved and paste it on the original OTU table, whose name is "otu_table_wout_unwant_OTUs.txt". Change the name of this file to "otu_table_wout_unwant_OTUs_edited1.txt".

#6) In Microbiome Helper terminal, transform the latter txt file into BIOM file, and then remove those OTUs whose taxonomy is still "Unassignable".

mkdir $HOME/pipits-1.5.0/fungi/out_process/making_final_OTU_table #this creates a new folder for increase clarity

biom convert -i $HOME/pipits-1.5.0/fungi/out_process/making_final_OTU_table/otu_table_wout_unwant_OTUs_edited1.txt -o $HOME/pipits-1.5.0/fungi/out_process/making_final_OTU_table/otu_table_process1.biom --to-hdf5 --table-type="OTU table" --process-obs-metadata taxonomy

filter_taxa_from_otu_table.py -i $HOME/pipits-1.5.0/fungi/out_process/making_final_OTU_table/otu_table_process1.biom -o $HOME/pipits-1.5.0/fungi/out_process/making_final_OTU_table/otu_table_process2f.biom -n Unassignable

biom convert -i $HOME/pipits-1.5.0/fungi/out_process/making_final_OTU_table/otu_table_process2f.biom -o $HOME/pipits-1.5.0/fungi/out_process/making_final_OTU_table/otu_table_process2f.txt --to-tsv --header-key taxonomy ##With this command, we create again a txt file to further editing the final OTU table

#7) In Excel, use the file "otu_table_process2f.txt" to add the taxonomic atrributes obtained in the second taxonomic assigning to those OTUs whose taxonomy reached only kingdom (Fungi) or Kingdom-Phylum. This is done on the basis of the txt file named "repseq_incompl_taxonomy_tax_assignments.txt". The final, modified file will be named "otu_table_process3.txt".

#8) Copy the latter file and paste it within the folder "making_final_OTU_table". Transform that file again into BIOM format.

```
biom convert -i $HOME/pipits-1.5.0/fungi/out_process/making_final_OTU_table/otu_table_process3.txt -o $HOME/pipits-1.5.0/fungi/out_process/making_final_OTU_table/otu_table_process4.biom --to-hdf5 --table-type="OTU table" --process-obs-metadata taxonomy
```

#9)<u>Sort samples in the BIOM table according to a particular criterion. To do this, first generate a "sample_ID_list.txt" with the desired criterion (e.g. number of sample as well as substrate type –soil and rock).</u>

```
sort_otu_table.py -i $HOME/pipits-1.5.0/fungi/out_process/making_final_OTU_table/otu_table_process4.biom -o $HOME/pipits-1.5.0/fungi/out_process/making_final_OTU_table/otu_table_process5.biom -l $HOME/pipits-1.5.0/fungi/out_process/sample_id_list.txt
```

#10) <u>Filter the BIOM table to remove low-confidence OTUs that are likely due to MISeq bleed-through between runs (reported by Illumina to be 0.1% of reads).</u>

```
remove_low_confidence_otus.py -i $HOME/pipits-1.5.0/fungi/out_process/making_final_OTU_table/otu_table_process5.biom -o $HOME/pipits-1.5.0/fungi/out_process/making_final_OTU_table/otu_table_process6.biom
```

#11) <u>Obtain a summary file of the BIOM table with and without removing these low confidence OTUs to check the changes.</u>

```
biom summarize-table -i $HOME/pipits-1.5.0/fungi/out_process/making_final_OTU_table/otu_table_process5.biom -o $HOME/pipits-1.5.0/fungi/out_process/making_final_OTU_table/otu_table_process5_without_remove_summary.txt
```

```
biom summarize-table -i $HOME/pipits-1.5.0/fungi/out_process/making_final_OTU_table/otu_table_process6.biom -o $HOME/pipits-1.5.0/fungi/out_process/making_final_OTU_table/otu_table_process6_with_remove_summary.txt
```

#12) <u>Remove samples with less than 10000 reads, and also sample R2_6H because it seems to be contaminated. To do this, create a txt file with the name of each samples to be removed in an independent row.</u>

```
filter_samples_from_otu_table.py -i $HOME/pipits-1.5.0/fungi/out_process/making_final_OTU_table/otu_table_process6.biom -o $HOME/pipits-1.5.0/fungi/out_process/making_final_OTU_table/otu_table_process7.biom --sample_id_fp $HOME/pipits-1.5.0/fungi/out_process/making_final_OTU_table/samples_IDs_to_remove.txt --negate_sample_id_fp
```

```
biom summarize-table -i $HOME/pipits-1.5.0/fungi/out_process/making_final_OTU_table/otu_table_process7.biom -o $HOME/pipits-1.5.0/fungi/out_process/making_final_OTU_table/otu_table_process7_samples_removed_summary.txt #to check the changes
```

#13) Add metadata (use a "mapAfun.txt").

```
biom add-metadata -i $HOME/pipits-1.5.0/fungi/out_process/making_final_OTU_table/otu_table_process7.biom -o $HOME/pipits-1.5.0/fungi/out_process/making_final_OTU_table/otu_table_def.biom --sample-metadata-fp $HOME/pipits-1.5.0/fungi/out_process/mapAfun.txt
```

########Summarize and PLOT TAXONOMY (answer: proportionally, what fungi are found in each sample community?)

```
mkdir $HOME/pipits-1.5.0/fungi/out_process/1_sum_plot_tax

summarize_taxa.py -i $HOME/pipits-1.5.0/fungi/out_process/making_final_OTU_table/otu_table_def.biom -L 2 -o $HOME/pipits-1.5.0/fungi/out_process/1_sum_plot_tax/

plot_taxa_summary.py -i $HOME/pipits-1.5.0/fungi/out_process/1_sum_plot_tax/otu_table_def_L2.txt -l phylum -c pie,bar,area -o $HOME/pipits-1.5.0/fungi/out_process/1_sum_plot_tax/phylum_charts/
```

##########ALPHA DIVERSITY (using rarefied and non rarefied datasets)

```
mkdir $HOME/pipits-1.5.0/fungi/out_process/2_final_otu_tables_rarefaction

single_rarefaction.py -i $HOME/pipits-1.5.0/fungi/out_process/making_final_OTU_table/otu_table_def.biom -o $HOME/pipits-1.5.0/fungi/out_process/2_final_otu_tables_rarefaction/otu_table_rarified.biom -d 13520

mkdir $HOME/pipits-1.5.0/fungi/out_process/3_alpha_diversity

alpha_diversity.py -i $HOME/pipits-1.5.0/fungi/out_process/making_final_OTU_table/otu_table_def.biom -m chao1,observed_otus,goods_coverage,shannon,simpson -o $HOME/pipits-1.5.0/fungi/out_process/3_alpha_diversity/alpha_diversity_metrics_not_rarif.txt

alpha_diversity.py -i $HOME/pipits-1.5.0/fungi/out_process/2_final_otu_tables_rarefaction/otu_table_rarified.biom -m chao1,observed_otus,goods_coverage,shannon,simpson -o $HOME/pipits-1.5.0/fungi/out_process/3_alpha_diversity/alpha_diversity_metrics_rarif.txt
```

#Generate rarefaction curves for the different metrics:

```
cd $HOME/pipits-1.5.0/fungi/out_process/3_alpha_diversity

echo 'alpha_diversity:metrics observed_species,shannon,chao1' > parameters_alphaDiver.txt

alpha_rarefaction.py -i $HOME/pipits-1.5.0/fungi/out_process/2_final_otu_tables_rarefaction/otu_table_rarified.biom -o $HOME/pipits-1.5.0/fungi/out_process/3_alpha_diversity/raref_curves -p $HOME/pipits-1.5.0/fungi/out_process/3_alpha_diversity/parameters_alphaDiver.txt -m $HOME/pipits-1.5.0/fungi/out_process/mapAfun.txt -e 13520
```

##########Normalize the OTU table by CSS method

```
mkdir $HOME/pipits-1.5.0/fungi/out_process/4_normalized_BIOM_table

normalize_table.py -i $HOME/pipits-1.5.0/fungi/out_process/making_final_OTU_table/otu_table_def.biom -a CSS -o $HOME/pipits-1.5.0/fungi/out_process/4_normalized_BIOM_table/CSS_normal_otu_table.biom
```

##########Beta-DIVERSITY and PCoA based on a Bray-Curtis dissimilarity matrix

```
mkdir $HOME/pipits-1.5.0/fungi/out_process/5_beta_diversity

beta_diversity.py -i $HOME/pipits-1.5.0/fungi/out_process/4_normalized_BIOM_table/CSS_normal_otu_table.biom -o $HOME/pipits-1.5.0/fungi/out_process/5_beta_diversity/ -m bray_curtis

principal_coordinates.py -i $HOME/pipits-1.5.0/fungi/out_process/5_beta_diversity/bray_curtis_CSS_normal_otu_table.txt -o $HOME/pipits-1.5.0/fungi/out_process/5_beta_diversity/bdiv_BrayCurt_coords.txt

make_2d_plots.py -i $HOME/pipits-1.5.0/fungi/out_process/5_beta_diversity/bdiv_BrayCurt_coords.txt -m $HOME/pipits-1.5.0/fungi/out_process/mapAfun.txt -o $HOME/pipits-1.5.0/fungi/out_process/5_beta_diversity/ #to graphic the PCoA 2d plot
```

##########Comparing Categories. Basic Question: Do different sample groupings significantly differ in their microbial composition?

##A) Compare whether there are significant differences between the fungal communities occurring on rocks and soils.

```
mkdir $HOME/pipits-1.5.0/fungi/out_process/6_statistical_tests

mkdir $HOME/pipits-1.5.0/fungi/out_process/6_statistical_tests/1_source_overall

compare_categories.py --method anosim -i $HOME/pipits-1.5.0/fungi/out_process/5_beta_diversity/bray_curtis_CSS_normal_otu_table.txt -m $HOME/pipits-1.5.0/fungi/out_process/mapAfun.txt -c Source -o $HOME/pipits-1.5.0/fungi/out_process/6_statistical_tests/1_source_overall/anosim_out
```

```
compare_categories.py --method adonis -i $HOME/pipits-1.5.0/fungi/out_process/5_beta_diversity/bray_curtis_CSS_normal_otu_table.txt -m $HOME/pipits-1.5.0/fungi/out_process/mapAfun.txt -c Source -o $HOME/pipits-1.5.0/fungi/out_process/6_statistical_tests/1_source_overall/adonis_out
```

##B) Compare whether there are significant differences between the fungal communities occurring on rocks and soils in the three-stage chronosequence.

#B1)Splitting BIOM table according to metadata file and category

```
mkdir $HOME/pipits-1.5.0/fungi/out_process/6_statistical_tests/2_otu_tables_bdiv_per_category

filter_samples_from_otu_table.py -i $HOME/pipits-1.5.0/fungi/out_process/making_final_OTU_table/otu_table_process7.biom -o $HOME/pipits-1.5.0/fungi/out_process/6_statistical_tests/2_otu_tables_bdiv_per_category/otu_table_rock.biom -m $HOME/pipits-1.5.0/fungi/out_process/mapAfun.txt -s 'Source:Rock'

filter_samples_from_otu_table.py -i $HOME/pipits-1.5.0/fungi/out_process/making_final_OTU_table/otu_table_process7.biom -o $HOME/pipits-1.5.0/fungi/out_process/6_statistical_tests/2_otu_tables_bdiv_per_category/otu_table_soil.biom -m $HOME/pipits-1.5.0/fungi/out_process/mapAfun.txt -s 'Source:Soil'
```

#B2)Manually generate two mapping files (“mapfunsoil.txt” and “mapfunrock.txt”) to add metadata to the two previous BIOM files. Save them in folder "2_otu_tables_bdiv_per_category".

#B3)Add metadata

```
biom add-metadata -i $HOME/pipits-1.5.0/fungi/out_process/6_statistical_tests/2_otu_tables_bdiv_per_category/otu_table_rock.biom -o $HOME/pipits-1.5.0/fungi/out_process/6_statistical_tests/2_otu_tables_bdiv_per_category/otu_table_rock_metadata.biom --sample-metadata-fp $HOME/pipits-1.5.0/fungi/out_process/6_statistical_tests/2_otu_tables_bdiv_per_category/mapfunrock.txt

biom add-metadata -i $HOME/pipits-1.5.0/fungi/out_process/6_statistical_tests/2_otu_tables_bdiv_per_category/otu_table_soil.biom -o $HOME/pipits-1.5.0/fungi/out_process/6_statistical_tests/2_otu_tables_bdiv_per_category/otu_table_soil_metadata.biom --sample-metadata-fp $HOME/pipits-1.5.0/fungi/out_process/6_statistical_tests/2_otu_tables_bdiv_per_category/mapfunsoil.txt
```

#B4)Normalize data in the two BIOM files by the CSS method

normalize_table.py -i $HOME/pipits-1.5.0/fungi/out_process/6_statistical_tests/2_otu_tables_bdiv_per_category/otu_table_rock_metadata.biom -a CSS -o $HOME/pipits-1.5.0/fungi/out_process/6_statistical_tests/2_otu_tables_bdiv_per_category/CSS_norm_otu_table_rock.biom

normalize_table.py -i $HOME/pipits-1.5.0/fungi/out_process/6_statistical_tests/2_otu_tables_bdiv_per_category/otu_table_soil_metadata.biom -a CSS -o $HOME/pipits-1.5.0/fungi/out_process/6_statistical_tests/2_otu_tables_bdiv_per_category/CSS_norm_otu_table_soil.biom

#B5)Create a new directory before conducting the analyses.

mkdir $HOME/pipits-1.5.0/fungi/out_process/6_statistical_tests/3_bdiv_per_category_PCoA

#######analyses of soil data#######

beta_diversity.py -i $HOME/pipits-1.5.0/fungi/out_process/6_statistical_tests/2_otu_tables_bdiv_per_category/CSS_norm_otu_table_soil.biom -o $HOME/pipits-1.5.0/fungi/out_process/6_statistical_tests/2_otu_tables_bdiv_per_category/3_bdiv_per_category_PCoA/bdiv_soil_BrayCurtis/ -m bray_curtis

principal_coordinates.py -i $HOME/pipits-1.5.0/fungi/out_process/6_statistical_tests/2_otu_tables_bdiv_per_category/3_bdiv_per_category_PCoA/bdiv_soil_BrayCurtis/bray_curtis_CSS_norm_otu_table_soil.txt -o $HOME/pipits-1.5.0/fungi/out_process/6_statistical_tests/2_otu_tables_bdiv_per_category/3_bdiv_per_category_PCoA/bdiv_soil_BrayCurtis/bdiv_soil_BrayCurt_coords.txt

make_2d_plots.py -i $HOME/pipits-1.5.0/fungi/out_process/6_statistical_tests/2_otu_tables_bdiv_per_category/3_bdiv_per_category_PCoA/bdiv_soil_BrayCurtis/bdiv_soil_BrayCurt_coords.txt -m $HOME/pipits-1.5.0/fungi/out_process/6_statistical_tests/2_otu_tables_bdiv_per_category/mapfunsoil.txt -o $HOME/pipits-1.5.0/fungi/out_process/6_statistical_tests/2_otu_tables_bdiv_per_category/3_bdiv_per_category_PCoA/bdiv_soil_BrayCurtis/

#######analyses of rock data#######

beta_diversity.py -i $HOME/pipits-1.5.0/fungi/out_process/6_statistical_tests/2_otu_tables_bdiv_per_category/CSS_norm_otu_table_rock.biom -o $HOME/pipits-1.5.0/fungi/out_process/6_statistical_tests/2_otu_tables_bdiv_per_category/3_bdiv_per_category_PCoA/bdiv_rock_BrayCurtis/ -m bray_curtis

principal_coordinates.py -i $HOME/pipits-1.5.0/fungi/out_process/6_statistical_tests/2_otu_tables_bdiv_per_category/3_bdiv_per_category_PCoA/bdiv_rock_BrayCurtis/bray_curtis_CSS_norm_otu_table_rock.txt -o $HOME/pipits-1.5.0/fungi/out_process/6_statistical_tests/2_otu_tables_bdiv_per_category/3_bdiv_per_category_PCoA/bdiv_rock_BrayCurtis/bdiv_rock_BrayCurt_coords.txt

make_2d_plots.py -i $HOME/pipits-1.5.0/fungi/out_process/6_statistical_tests/2_otu_tables_bdiv_per_category/3_bdiv_per_category_PCoA/bdiv_rock_BrayCurtis/bdiv_rock_BrayCurt_coords.txt -m $HOME/pipits-1.5.0/fungi/out_process/6_statistical_tests/2_otu_tables_bdiv_per_category/mapfunrock.txt -o $HOME/pipits-1.5.0/fungi/out_process/6_statistical_tests/2_otu_tables_bdiv_per_category/3_bdiv_per_category_PCoA/bdiv_rock_BrayCurtis/

#B6)Run ADONIS y ANOSIM to test whether the sample groupings revealed in the PCoA with respect to time are supported or not

#######analyses of soil data#######

mkdir $HOME/pipits-1.5.0/fungi/out_process/6_statistical_tests/4_time_soil

compare_categories.py --method anosim -i $HOME/pipits-1.5.0/fungi/out_process/6_statistical_tests/2_otu_tables_bdiv_per_category/3_bdiv_per_category_PCoA/bdiv_soil_BrayCurtis/bray_curtis_CSS_norm_otu_table_soil.txt -m $HOME/pipits-1.5.0/fungi/out_process/6_statistical_tests/2_otu_tables_bdiv_per_category/mapfunsoil.txt -c Time -o $HOME/pipits-1.5.0/fungi/out_process/6_statistical_tests/4_time_soil/anosim_out_time_soil

compare_categories.py --method adonis -i $HOME/pipits-1.5.0/fungi/out_process/6_statistical_tests/2_otu_tables_bdiv_per_category/3_bdiv_per_category_PCoA/bdiv_soil_BrayCurtis/bray_curtis_CSS_norm_otu_table_soil.txt -m $HOME/pipits-1.5.0/fungi/out_process/6_statistical_tests/2_otu_tables_bdiv_per_category/mapfunsoil.txt -c Time -o $HOME/pipits-1.5.0/fungi/out_process/6_statistical_tests/4_time_soil/adonis_out_time_soil

#######analyses of rock data#######

mkdir $HOME/pipits-1.5.0/fungi/out_process/6_statistical_tests/5_time_rocks

compare_categories.py --method anosim -i $HOME/pipits-1.5.0/fungi/out_process/6_statistical_tests/2_otu_tables_bdiv_per_category/3_bdiv_per_category_PCoA/bdiv_rock_BrayCurtis/bray_curtis_CSS_norm_otu_table_rock.txt -m $HOME/pipits-

1.5.0/fungi/out_process/6_statistical_tests/2_otu_tables_bdiv_per_category/mapfunrock.txt -c Time -o $HOME/pipits-1.5.0/fungi/out_process/6_statistical_tests/5_time_rocks/anosim_out_time_rock

compare_categories.py --method adonis -i $HOME/pipits-1.5.0/fungi/out_process/6_statistical_tests/2_otu_tables_bdiv_per_category/3_bdiv_per_category_PCoA/bdiv_rock_BrayCurtis/bray_curtis_CSS_norm_otu_table_rock.txt -m $HOME/pipits-1.5.0/fungi/out_process/6_statistical_tests/2_otu_tables_bdiv_per_category/mapfunrock.txt -c Time -o $HOME/pipits-1.5.0/fungi/out_process/6_statistical_tests/5_time_rocks/adonis_out_time_rock

##

Ic) Operating procedure for analyzing fungal *rbcL* data to infer 97% OTUs using the Microbiome Helper virtual box (Comeau et al., 2017; https://github.com/mlangill/microbiome_helper/wiki/16S-standard-operating-180 procedure), with some custom modifications.

Date: 26-APR-2018; Author: Isaac Garrido Benavent; Software: MV Microbiome Helper amplicon v 0.3

Primer used for amplification of RuBisCO region (Nozaki et al. 1995):

CS1-rbcL650 (F): GTTTCCTTTTCGTAGCTGAAGC and CS2-rbcL830 (R): TTAGCTGTGAAACCACCTGTTA

#########

source /home/mh_user/anaconda2/bin/activate qiime1 #esto es para activar qiime; se debe hacer siempre

#########Check that the pair-end data is sorted

cd /home/mh_user/Desktop/clus_97_algae

mkdir 1_algae_sorted_fastqs

parallel -j 20 'cat {} | paste - - - - | sort -k1,1 -t " " | tr "\t" "\n" > 1_algae_sorted_fastqs/{/.}.sorted.fastq' ::: rawdata/*.fastq

#########Stitching paired-end reads

cd ~/Desktop/clus_97_algae

run_pear.pl -p 2 -o 3_stitched_reads 1_algae_sorted_fastqs/*fastq ## ("-p 1" indicates this job should be run on 1 CPU and "-o stitched_reads" indicates that the output folder)

#########Quality metrics of stitched reads with FastQC.

mkdir 4_fastqc_out_combined

cat 3_stitched_reads/*.assembled.fastq | fastqc -t 1 stdin -o 4_fastqc_out_combined

cd 4_fastqc_out_combined

mv stdin_fastqc.html combined_fastqc.html

mv stdin_fastqc.zip combined_fastqc.zip

#########Filter reads based on Phred values

cd ~/Desktop/clus_97_algae

read_filter.pl -f GTTTCCTTTTCGTAGCTGAAGC -r TAACAGGTGGTTTCACAGCTAA -q 28 -p 90 -l 120 -thread 3 -o 5_filtered_reads -c both -t 3_stitched_reads/*.assembled*fastq

#########Conversion to FASTA

cd ~/Desktop/clus_97_algae

run_fastq_to_fasta.pl -p 2 -o 6_fasta_files 5_filtered_reads/*fastq #this command also allows removing sequences containing "N"

#########Removal of chimeric reads using two consecutive strategies: *de novo*, and reference-based

#1)*De novo* removal

mkdir 7_fasta_files_derep

mkdir 8_non_denovo_chimeras

mkdir 9_non_denovo_chimeras_rerep

parallel -j 2 'vsearch --derep_fulllength {} --sizeout --output 7_fasta_files_derep/{/.}.derep.fasta' ::: 6_fasta_files/*fasta

parallel --eta -j 2 'vsearch --uchime_denovo {} --nonchimeras 8_non_denovo_chimeras/{/.}.nonchimera.fasta 2> 8_non_denovo_chimeras/{/.}.nonchimera.log' ::: 7_fasta_files_derep/*.derep.fasta

parallel -j 2 'vsearch --rereplicate {} --output 9_non_denovo_chimeras_rerep/{/.}.rerep.fasta' ::: 8_non_denovo_chimeras/*fasta

#2)Removal based on a reference database (in this case, a custom database generated with GenBank *rbcL* data) and VSEARCH

chimera_filter.pl -type 1 -thread 3 -db /home/mh_user/Desktop/Custom_databases/qiime_database_algae/DB.fasta 9_non_denovo_chimeras_rerep/*fasta

#########Formatting the input FASTA files and generate a single FASTA file with adequate labels. But, first, create mapping file ("mapalga.txt") that includes a list of the samples as well as any metadata.

add_qiime_labels.py -i non_chimeras/ -m mapalga.txt -c FileInput -o 10_combined_fasta

#########Remove any further sequence artifact (e.gg extremely long sequence reads) using seqkit (Shen et al. 2016).

sudo conda install -c bioconda seqkit #to install seqkit (Shen et al. 2016).

#Generate some sequence statistics (e.g. length in bp) before and after trimming with seqkit

```
count_seqs.py -i 10_combined_fasta/combined_seqs.fna -o 10_combined_fasta/stat_combined_seqs.txt

cd 10_combined_fasta

cat combined_seqs.fna | seqkit seq | seqkit stats >> seq_stats_previous_trimming.txt

cat combined_seqs.fna | '/home/mh_user/anaconda2/bin/seqkit' seq -m 120 -M 150 | '/home/mh_user/anaconda2/bin/seqkit' stats >> seq_stats_after_trimming.txt #hacer lo mismo que antes aquí

'/home/mh_user/anaconda2/bin/seqkit' seq -m 120 -M 150 combined_seqs.fna > lengthfiltered_combined_seqs.fna #hacer lo mismo con la palabra seqkit

cd ..

count_seqs.py -i combined_fasta/lengthfiltered_combined_seqs.fna -o combined_fasta/after_seqkit_stat_combined_seqs.txt
```

#########Run open-reference OTU picking pipeline: using SORTMERNA and the custom database generated with GenBank *rbcL* data

#First, generate an alignment of the rcbL sequences downloaded from GenBank with mafft.

```
align_seqs.py -i /home/mh_user/Desktop/Custom_databases/qiime_database_algae/DB.fasta -m mafft -o /home/mh_user/Desktop/Custom_databases/qiime_database_algae/

mkdir ~/Desktop/clus_97_algae/11_clustering

cd ~/Desktop/clus_97_algae/11_clustering

echo "pick_otus:threads 3" >> clustering_params.txt

echo "pick_otus:enable_rev_strand_match True" >> clustering_params.txt

echo "pick_otus:sortmerna_coverage 0.8" >> clustering_params.txt

echo "pick_otus:similarity 0.97" >> clustering_params.txt
```

```
echo "align_seqs:template_fp /home/mh_user/Desktop/Custom_databases/qiime_database_algae/DB_aligned.fasta" >> clustering_params.txt

echo "filter_alignment:allowed_gap_frac 0.80" >> clustering_params.txt

echo "filter_alignment:entropy_threshold 0.10" >> clustering_params.txt

echo "filter_alignment:suppress_lane_mask_filter True" >> clustering_params.txt

echo "assign_taxonomy:reference_seqs_fp /home/mh_user/Desktop/Custom_databases/qiime_database_algae/DB.fasta" >> clustering_params.txt

echo "assign_taxonomy:id_to_taxonomy_fp /home/mh_user/Desktop/Custom_databases/qiime_database_algae/Taxonomy_final.txt" >> clustering_params.txt

pick_open_reference_otus.py -i /home/mh_user/Desktop/clus_97_algae/10_combined_fasta/lengthfiltered_combined_seqs.fna -o /home/mh_user/Desktop/clus_97_algae/11_clustering/1_pick_otus_sortmerna/ -p /home/mh_user/Desktop/clus_97_algae/11_clustering/clustering_params.txt -m sortmerna_sumaclust -r /home/mh_user/Desktop/Custom_databases/qiime_database_algae/DB.fasta -s 0.1 -v --min_otu_size 1
```

#########<u>Remove low confidence OTUs</u>

```
mkdir ~/Desktop/clus_97_algae/11_clustering/2_processed_BIOM_tables

cd ~/Desktop/clus_97_algae/11_clustering

remove_low_confidence_otus.py -i $PWD/1_pick_otus_sortmerna/otu_table_mc1_w_tax_no_pynast_failures.biom -o $PWD/2_processed_BIOM_tables/1_otu_table_high_conf.biom
```

#########<u>Compare results of the two BIOM files.</u>

```
mkdir ~/Desktop/clus_97_algae/11_clustering/3_BIOM_tables_summaries

biom summarize-table -i $PWD/1_pick_otus_sortmerna/otu_table_mc1_w_tax_no_pynast_failures.biom -o $PWD/3_BIOM_tables_summaries/1_otu_table_mc1_w_tax_no_pynast_failures_summary.txt

biom summarize-table -i $PWD/2_processed_BIOM_tables/1_otu_table_high_conf.biom -o $PWD/3_BIOM_tables_summaries/2_otu_table_high_conf_summary.txt
```

#########<u>Inspect the OTU table with its taxonomy assignments in Excel</u>

biom convert -i $PWD/2_processed_BIOM_tables/1_otu_table_high_conf.biom -o $PWD/3_BIOM_tables_summaries/1_otu_table_high_conf_txt_EXCEL.txt --to-tsv --header-key taxonomy

#########Further editing of the BIOM table (steps #a-#i)#########

#########Reassign the taxonomy of "Unassignable" OTUs with the SortMerna method.

#a) First, create a txt file with the OTU ID of those OTUs whose taxonomic assignment may be improved such as the "Unassignable". Thus, the txt file would be named "OTU_IDs_unassign.txt". Send this file to folder "2_processed_BIOM_tables".

#b)Again in the Microbiome Helper terminal, execute the following command on the BIOM file:

filter_otus_from_otu_table.py -i $PWD/2_processed_BIOM_tables/1_otu_table_high_conf.biom -o $PWD/2_processed_BIOM_tables/2_otu_table_unassigned.biom -e /home/mh_user/Desktop/clus_97_algae/11_clustering/2_processed_BIOM_tables/OTU_IDs_unassign.txt --negate_ids_to_exclude

#c)Use the modified BIOM file to select the corresponding sequences from the representative, general FASTA file.

filter_fasta.py -f /home/mh_user/Desktop/clus_97_algae/11_clustering/1_pick_otus_sortmerna/rep_set.fna -b $PWD/2_processed_BIOM_tables/2_otu_table_unassigned.biom -o /home/mh_user/Desktop/clus_97_algae/11_clustering/2_processed_BIOM_tables/repseq_unassigned.fna

#d)Assign taxonomy to the extracted fasta sequences.

assign_taxonomy.py -i /home/mh_user/Desktop/clus_97_algae/11_clustering/2_processed_BIOM_tables/repseq_unassigned.fna -t /home/mh_user/Desktop/Custom_databases/qiime_database_algae/Taxonomy_final.txt -r /home/mh_user/Desktop/Custom_databases/qiime_database_algae/DB.fasta -m sortmerna --sortmerna_threads 3 -o /home/mh_user/Desktop/clus_97_algae/11_clustering/2_processed_BIOM_tables/

#e)Manually, in Excel, copy the Tax IDs of the OTUs whose taxonomic assignments have improved and paste it on the original OTU table, whose name is "1_otu_table_high_conf_txt_EXCEL.txt". Change the name of this file to "2_otu_table_high_conf_edited.txt". Copy that file and paste it into folder "2_processed_BIOM_tables".

#f) In Microbiome Helper terminal, transform the latter txt file into BIOM file, and then remove those OTUs whose taxonomy is still "Unassigned".

biom convert -i /home/mh_user/Desktop/clus_97_algae/11_clustering/2_processed_BIOM_tables/2_otu_table_high_conf_edited.txt -o /home/mh_user/Desktop/clus_97_algae/11_clustering/2_processed_BIOM_tables/3_otu_table_improved_tax.biom --to-hdf5 --table-type="OTU table" --process-obs-metadata taxonomy

filter_taxa_from_otu_table.py -i /home/mh_user/Desktop/clus_97_algae/11_clustering/2_processed_BIOM_tables/3_otu_table_improved_tax.biom -o /home/mh_user/Desktop/clus_97_algae/11_clustering/2_processed_BIOM_tables/4_otu_table_improved_tax_filt.biom -n Unassigned

#g)Sort samples in the BIOM table according to a particular criterion. To do this, first generate a "sample_ID_list.txt" with the desired criterion (e.g. number of sample as well as substrate type –soil and rock).

sort_otu_table.py -i /home/mh_user/Desktop/clus_97_algae/11_clustering/2_processed_BIOM_tables/4_otu_table_improved_tax_filt.biom -o /home/mh_user/Desktop/clus_97_algae/11_clustering/2_processed_BIOM_tables/5_otu_table_sorted.biom -l /home/mh_user/Desktop/clus_97_algae/sample_ID_list.txt

#h)Add metadata to BIOM file.

biom add-metadata -i /home/mh_user/Desktop/clus_97_algae/11_clustering/2_processed_BIOM_tables/5_otu_table_sorted.biom -o /home/mh_user/Desktop/clus_97_algae/11_clustering/2_processed_BIOM_tables/6_otu_table_DEF.biom --sample-metadata-fp /home/mh_user/Desktop/clus_97_algae/mapalga.txt

#i)Create a summary of the BIOM and save it.

biom summarize-table -i /home/mh_user/Desktop/clus_97_algae/11_clustering/2_processed_BIOM_tables/6_otu_table_DEF.biom -o /home/mh_user/Desktop/clus_97_algae/11_clustering/3_BIOM_tables_summaries/3_otu_table_DEF_summary.txt.

############

############ALPHA- and BETA-DIVERSITY ANALYSES############

############Summarize and PLOT TAXONOMY (answer: proportionally, what fungi are found in each sample community?)

mkdir ~/Desktop/clus_97_algae/13_sum_plot_tax

summarize_taxa.py -i /home/mh_user/Desktop/clus_97_algae/11_clustering/2_processed_BIOM_tables/6_otu_table_DEF.biom -L 4 -o /home/mh_user/Desktop/clus_97_algae/13_sum_plot_tax/

plot_taxa_summary.py -i /home/mh_user/Desktop/clus_97_algae/13_sum_plot_tax/6_otu_table_DEF_L4.txt -l order -c pie,bar,area -o /home/mh_user/Desktop/clus_97_algae/13_sum_plot_tax/phylum_charts/

##########ALPHA-DIVERSITY (based on rarefied and non-rarefied datasets)

mkdir ~/Desktop/clus_97_algae/14_alpha_diversity

mkdir ~/Desktop/clus_97_algae/14_alpha_diversity/1_final_otu_tables_rarefaction

single_rarefaction.py -i /home/mh_user/Desktop/clus_97_algae/11_clustering/2_processed_BIOM_tables/6_otu_table_DEF.biom -o /home/mh_user/Desktop/clus_97_algae/14_alpha_diversity/1_final_otu_tables_rarefaction/otu_table_rarified.biom -d 18063

mkdir ~/Desktop/clus_97_algae/14_alpha_diversity/2_alpha_diversity

alpha_diversity.py -i /home/mh_user/Desktop/clus_97_algae/11_clustering/2_processed_BIOM_tables/6_otu_table_DEF.biom -m ace,chao1,observed_otus,goods_coverage,shannon,simpson -o /home/mh_user/Desktop/clus_97_algae/14_alpha_diversity/2_alpha_diversity/1_alpha_diversity_metrics_not_rarif.txt

alpha_diversity.py -i /home/mh_user/Desktop/clus_97_algae/14_alpha_diversity/1_final_otu_tables_rarefaction/otu_table_rarified.biom -m ace,chao1,observed_otus,goods_coverage,shannon,simpson -o /home/mh_user/Desktop/clus_97_algae/14_alpha_diversity/2_alpha_diversity/2_alpha_diversity_metrics_rarif.txt

#Generate rarefaction curves for the different metrics:

cd ~/Desktop/clus_97_algae/14_alpha_diversity

echo 'alpha_diversity:metrics observed_species,shannon,chao1' > parameters_alphaDiver.txt

alpha_rarefaction.py -i /home/mh_user/Desktop/clus_97_algae/14_alpha_diversity/1_final_otu_tables_rarefaction/otu_table_rarified.biom -o /home/mh_user/Desktop/clus_97_algae/14_alpha_diversity/3_rarefaction_curves -p /home/mh_user/Desktop/clus_97_algae/14_alpha_diversity/parameters_alphaDiver.txt -m /home/mh_user/Desktop/clus_97_algae/mapalga.txt -e 18063

##########Normalize the OTU table by the CSS method

mkdir ~/Desktop/clus_97_algae/15_normalized_BIOM_table

```
normalize_table.py -i /home/mh_user/Desktop/clus_97_algae/11_clustering/2_processed_BIOM_tables/6_otu_table_DEF.biom -a CSS -o /home/mh_user/Desktop/clus_97_algae/15_normalized_BIOM_table/CSS_normal_otu_table.biom
```

##########Beta-DIVERSITY and PCoA using the Bray-Curtis dissimilarity matrix

```
mkdir ~/Desktop/clus_97_algae/16_beta_diversity

beta_diversity.py -i /home/mh_user/Desktop/clus_97_algae/15_normalized_BIOM_table/CSS_normal_otu_table.biom -o /home/mh_user/Desktop/clus_97_algae/16_beta_diversity/ -m bray_curtis

principal_coordinates.py -i /home/mh_user/Desktop/clus_97_algae/16_beta_diversity/bray_curtis_CSS_normal_otu_table.txt -o /home/mh_user/Desktop/clus_97_algae/16_beta_diversity/bdiv_BrayCurt_coords.txt #para hacer PCoA basado en matriz de distancias Bray-Curtis

make_2d_plots.py -i /home/mh_user/Desktop/clus_97_algae/16_beta_diversity/bdiv_BrayCurt_coords.txt -m /home/mh_user/Desktop/clus_97_algae/mapalga.txt -o /home/mh_user/Desktop/clus_97_algae/16_beta_diversity/ #to graphic PCoA 2d plot
```

##########Comparing Categories. Basic Question: Do different sample groupings significantly differ in their microbial composition?

##A) Compare whether there are significant differences between the algal communities occurring on rocks and soils.

```
mkdir ~/Desktop/clus_97_algae/17_statistical_tests

mkdir ~/Desktop/clus_97_algae/17_statistical_tests/1_source_overall

compare_categories.py --method anosim -i /home/mh_user/Desktop/clus_97_algae/16_beta_diversity/bray_curtis_CSS_normal_otu_table.txt -m /home/mh_user/Desktop/clus_97_algae/mapalga.txt -c Source -o /home/mh_user/Desktop/clus_97_algae/17_statistical_tests/1_source_overall/anosim_out

compare_categories.py --method adonis -i /home/mh_user/Desktop/clus_97_algae/16_beta_diversity/bray_curtis_CSS_normal_otu_table.txt -m /home/mh_user/Desktop/clus_97_algae/mapalga.txt -c Source -o /home/mh_user/Desktop/clus_97_algae/17_statistical_tests/1_source_overall/adonis_out
```

##B)Compare whether there are significant differences between the fungal communities occurring on rocks and soils in the three-stage chronosequence

#B1)Splitting BIOM table according to metadata file and category

```
mkdir ~/Desktop/clus_97_algae/17_statistical_tests/2_otu_tables_bdiv_per_category
```

```
filter_samples_from_otu_table.py -i /home/mh_user/Desktop/clus_97_algae/11_clustering/2_processed_BIOM_tables/5_otu_table_sorted.biom -o /home/mh_user/Desktop/clus_97_algae/17_statistical_tests/2_otu_tables_bdiv_per_category/otu_table_rock.biom -m /home/mh_user/Desktop/clus_97_algae/mapalga.txt -s 'Source:Rock'

filter_samples_from_otu_table.py -i /home/mh_user/Desktop/clus_97_algae/11_clustering/2_processed_BIOM_tables/5_otu_table_sorted.biom -o /home/mh_user/Desktop/clus_97_algae/17_statistical_tests/2_otu_tables_bdiv_per_category/otu_table_soil.biom -m /home/mh_user/Desktop/clus_97_algae/mapalga.txt -s 'Source:Soil'
```

#B2) Manually generate two mapping files (“mapalgasoil.txt” and “mapalgarock.txt”) to add metadata to the two previous BIOM files. Save them in folder "2_otu_tables_bdiv_per_category".

#B3)Add metadata to these two BIOM files.

```
biom add-metadata -i /home/mh_user/Desktop/clus_97_algae/17_statistical_tests/2_otu_tables_bdiv_per_category/otu_table_rock.biom -o /home/mh_user/Desktop/clus_97_algae/17_statistical_tests/2_otu_tables_bdiv_per_category/otu_table_rock_metadata.biom --sample-metadata-fp /home/mh_user/Desktop/clus_97_algae/17_statistical_tests/2_otu_tables_bdiv_per_category/mapalgarock.txt

biom add-metadata -i /home/mh_user/Desktop/clus_97_algae/17_statistical_tests/2_otu_tables_bdiv_per_category/otu_table_soil.biom -o /home/mh_user/Desktop/clus_97_algae/17_statistical_tests/2_otu_tables_bdiv_per_category/otu_table_soil_metadata.biom --sample-metadata-fp /home/mh_user/Desktop/clus_97_algae/17_statistical_tests/2_otu_tables_bdiv_per_category/mapalgasoil.txt
```

#B4)Normalize the BIOM files by the CSS method.

```
mkdir ~/Desktop/clus_97_algae/17_statistical_tests/3_otu_tables_bdiv_NORM

normalize_table.py -i /home/mh_user/Desktop/clus_97_algae/17_statistical_tests/2_otu_tables_bdiv_per_category/otu_table_rock_metadata.biom -a CSS -o /home/mh_user/Desktop/clus_97_algae/17_statistical_tests/3_otu_tables_bdiv_NORM/CSS_norm_otu_table_rock.biom

normalize_table.py -i /home/mh_user/Desktop/clus_97_algae/17_statistical_tests/2_otu_tables_bdiv_per_category/otu_table_soil_metadata.biom -a CSS -o
```

/home/mh_user/Desktop/clus_97_algae/17_statistical_tests/3_otu_tables_bdiv_NORM/CSS_norm_otu_table_soil.biom

#B5)Create a new directory before conducting the analyses.

```
mkdir ~/Desktop/clus_97_algae/17_statistical_tests/4_bdiv_per_category_PCoA
```

#######analyses of soil data#######

```
beta_diversity.py -i /home/mh_user/Desktop/clus_97_algae/17_statistical_tests/3_otu_tables_bdiv_NORM/CSS_norm_otu_table_soil.biom -o /home/mh_user/Desktop/clus_97_algae/17_statistical_tests/4_bdiv_per_category_PCoA/bdiv_soil_BrayCurtis/ -m bray_curtis

principal_coordinates.py -i /home/mh_user/Desktop/clus_97_algae/17_statistical_tests/4_bdiv_per_category_PCoA/bdiv_soil_BrayCurtis/bray_curtis_CSS_norm_otu_table_soil.txt -o /home/mh_user/Desktop/clus_97_algae/17_statistical_tests/4_bdiv_per_category_PCoA/bdiv_soil_BrayCurtis/bdiv_soil_BrayCurt_coords.txt

make_2d_plots.py -i /home/mh_user/Desktop/clus_97_algae/17_statistical_tests/4_bdiv_per_category_PCoA/bdiv_soil_BrayCurtis/bdiv_soil_BrayCurt_coords.txt -m /home/mh_user/Desktop/clus_97_algae/17_statistical_tests/2_otu_tables_bdiv_per_category/mapalgasoil.txt -o /home/mh_user/Desktop/clus_97_algae/17_statistical_tests/4_bdiv_per_category_PCoA/bdiv_soil_BrayCurtis/
```

#######analyses of rock data#######

```
beta_diversity.py -i /home/mh_user/Desktop/clus_97_algae/17_statistical_tests/3_otu_tables_bdiv_NORM/CSS_norm_otu_table_rock.biom -o /home/mh_user/Desktop/clus_97_algae/17_statistical_tests/4_bdiv_per_category_PCoA/bdiv_rock_BrayCurtis/ -m bray_curtis

principal_coordinates.py -i /home/mh_user/Desktop/clus_97_algae/17_statistical_tests/4_bdiv_per_category_PCoA/bdiv_rock_BrayCurtis/bray_curtis_CSS_norm_otu_table_rock.txt -o /home/mh_user/Desktop/clus_97_algae/17_statistical_tests/4_bdiv_per_category_PCoA/bdiv_rock_BrayCurtis/bdiv_rock_BrayCurt_coords.txt

make_2d_plots.py -i /home/mh_user/Desktop/clus_97_algae/17_statistical_tests/4_bdiv_per_category_PCoA/bdiv_rock_BrayCurtis/bdiv_rock_BrayCurt_coords.txt -m /home/mh_user/Desktop/clus_97_algae/17_statistical_tests/2_otu_tables_bdiv_per_category/mapalgarock.txt -o /home/mh_user/Desktop/clus_97_algae/17_statistical_tests/4_bdiv_per_category_PCoA/bdiv_rock_BrayCurtis/
```

#B6)Run ADONIS y ANOSIM to test whether the sample groupings revealed in the PCoA with respect to time are supported or not

#######analyses of soil data#######

```
mkdir ~/Desktop/clus_97_algae/17_statistical_tests/5_stat_tests_time_soil

compare_categories.py --method anosim -i
/home/mh_user/Desktop/clus_97_algae/17_statistical_tests/4_bdiv_per_category_PCoA
/bdiv_soil_BrayCurtis/bray_curtis_CSS_norm_otu_table_soil.txt -m
/home/mh_user/Desktop/clus_97_algae/17_statistical_tests/2_otu_tables_bdiv_per_cate
gory/mapalgasoil.txt -c Time -o
/home/mh_user/Desktop/clus_97_algae/17_statistical_tests/5_stat_tests_time_soil/anosi
m_out_time_soil

compare_categories.py --method adonis -i
/home/mh_user/Desktop/clus_97_algae/17_statistical_tests/4_bdiv_per_category_PCoA
/bdiv_soil_BrayCurtis/bray_curtis_CSS_norm_otu_table_soil.txt -m
/home/mh_user/Desktop/clus_97_algae/17_statistical_tests/2_otu_tables_bdiv_per_cate
gory/mapalgasoil.txt -c Time -o
/home/mh_user/Desktop/clus_97_algae/17_statistical_tests/5_stat_tests_time_soil/adonis
_out_time_soil
```

#######analyses of rock data#######

```
mkdir ~/Desktop/clus_97_algae/17_statistical_tests/6_stat_tests_time_rock

compare_categories.py --method anosim -i
/home/mh_user/Desktop/clus_97_algae/17_statistical_tests/4_bdiv_per_category_PCoA
/bdiv_rock_BrayCurtis/bray_curtis_CSS_norm_otu_table_rock.txt -m
/home/mh_user/Desktop/clus_97_algae/17_statistical_tests/2_otu_tables_bdiv_per_cate
gory/mapalgarock.txt -c Time -o
/home/mh_user/Desktop/clus_97_algae/17_statistical_tests/6_stat_tests_time_rock/anosi
m_out_time_rock

compare_categories.py --method adonis -i
/home/mh_user/Desktop/clus_97_algae/17_statistical_tests/4_bdiv_per_category_PCoA
/bdiv_rock_BrayCurtis/bray_curtis_CSS_norm_otu_table_rock.txt -m
/home/mh_user/Desktop/clus_97_algae/17_statistical_tests/2_otu_tables_bdiv_per_cate
gory/mapalgarock.txt -c Time -o
/home/mh_user/Desktop/clus_97_algae/17_statistical_tests/6_stat_tests_time_rock/adon
is_out_time_rock
```

##

II) INFERENCE OF Amplicon Sequence Variants (ASVs)

IIa) Operating procedure for analyzing bacterial 16S data to infer ASVs using the Microbiome Helper virtual box (Comeau et al., 2017; https://github.com/mlangill/microbiome_helper/wiki/16S-standard-operating-180 procedure) and dada2 (Callahan et al., 2016) scripts, with some custom modifications.

Date: 25-APR-2018; Author: Isaac Garrido Benavent; Software: MV Microbiome Helper amplicon v 0.3 and dada2 (Callahan et al., 2016)

Primer used for amplification of the V1-V2 region within the 16AS:

27F: AGAGTTTGATCMTGGCTCAG and 338R: CATGCTGCCTCCCGTAGGAGT

#########

```
source /home/mh_user/anaconda2/bin/activate qiime1
```

#########Check paired-end data and order them according to sample name

```
cd ~/Desktop/dadabac

mkdir 1_bac_sorted_fastqs

parallel -j 20 'cat {} | paste - - - - | sort -k1,1 -t " " | tr "\t" "\n" > 1_bac_sorted_fastqs/{/.}.sorted.fastq' ::: rawdata/*.fastq
```

#########Remove primer sequences

```
cd ~/Desktop/dadabac

mkdir 2_primer_trimmed_fastqs

parallel --link --jobs 9 'cutadapt --pair-filter any --no-indels --discard-untrimmed -g AGAGTTTGATCNTGGCTCAG -G CATGCTGCCTCCCGTAGGAGT -o 2_primer_trimmed_fastqs/{1/}.gz -p 2_primer_trimmed_fastqs/{2/}.gz {1} {2} > 2_primer_trimmed_fastqs/{1/}_cutadapt_log.txt' ::: 1_bac_sorted_fastqs/*_R1_*.fastq ::: 1_bac_sorted_fastqs/*_R2_*.fastq
```

#in the primer sequence, any ambiguous base should be denoted by "N"; don't use R, S, M, and so on.

#To summarize cutadapt results in a single file:

```
parse_cutadapt_logs.py -i 2_primer_trimmed_fastqs/*log.txt
```

#########Obtaining quality metrics of stitched reads with FASTQC

```
mkdir 3_primer_trimmed_fastqc_out

fastqc -t 9 2_primer_trimmed_fastqs/*fastq.gz -o 3_primer_trimmed_fastqc_out/
```

#########Filtering reads by quality (take a look at the dada2 tutorial to understand each portion in the command below)

dada2_filter.R -f 2_primer_trimmed_fastqs --trimLeft 10 --truncLen 260,160 --maxN 0 --maxEE 3,7 --truncQ 2 --threads 9 --f_match _R1_.*fastq.gz --r_match _R2_.*fastq.gz

#IMPORTANT: this command generates a default folder named "filtered_fastqs". Rename to "4_filtered_fastqs".

#########Infer sequence variants (i.e. ASVs)

cd ~/Desktop/dadabac

dada2_inference.R -f 4_filtered_fastqs --seed 4124 -t 9 --verbose --plot_errors

#########Chimera checking and Taxonomy assignment

dada2_chimera_taxa.R -i seqtab.rds -r ~/Documents/dada2_rdp_ref/silva_nr_v128_train_set.fa.gz --skip_species -t 9

#Important: to download the SILVA file use the following link (https://zenodo.org/record/801832#.WquXYpdG3IU)

#########Combining logfiles to this point

merge_logfiles.R -i cutadapt_log.txt,dada2_filter_read_counts.txt,dada2_inferred_read_counts.txt,dada2_nonchimera_counts.txt -n cutadapt,filter,infer,chimera -o combined_log.txt

mkdir 5_output_and_log_files

#########Convert dada2 output to BIOM and FASTA

convert_dada2_out.R -i seqtab_final.rds -b seqtab.biom -f seqtab.fasta --taxa_in tax_final.rds

##########Remove any further sequence artifact (e.gg extremely long sequence reads) using seqkit (Shen et al. 2016).

sudo conda install -c bioconda seqkit #this command installs seqkit

#Generate some sequence statistics (e.g. length in bp) before and after trimming with seqkit.

mkdir 6_seq_stats

count_seqs.py -i seqtab.fasta -o 6_seq_stats/1_stats_original_data.txt

```
cat seqtab.fasta | '/home/mh_user/anaconda2/bin/seqkit' seq | '/home/mh_user/anaconda2/bin/seqkit' stats >> 6_seq_stats/2_seq_stats_previous_trimming.txt

cat seqtab.fasta | '/home/mh_user/anaconda2/bin/seqkit' seq -m 265 -M 305 | '/home/mh_user/anaconda2/bin/seqkit' stats >> 6_seq_stats/3_seq_stats_after_trimming.txt

'/home/mh_user/anaconda2/bin/seqkit' seq -m 265 -M 305 seqtab.fasta > 6_seq_stats/lengthfiltered_seqtab.fna

count_seqs.py -i 6_seq_stats/lengthfiltered_seqtab.fna -o 6_seq_stats/4_after_seqkit_stat_combined_seqs.txt
```

##################### EDITING THE BIOM TABLE #################

##########a)Add "taxonomy labels" as metadata to the BIOM table

```
cd ~/Desktop/dadabac

mkdir 7_BIOM_table_processing

biom add-metadata -i seqtab.biom -o /home/mh_user/Desktop/dadabac/7_BIOM_table_processing/1_seqtab_tax.biom --observation-metadata-fp  taxa_metadata.txt --observation-header OTUID,taxonomy --sc-separated taxonomy
```

#b)Filter the BIOM table to keep only the ASVs with a desired

```
filter_otus_from_otu_table.py -i /home/mh_user/Desktop/dadabac/7_BIOM_table_processing/1_seqtab_tax.biom -o /home/mh_user/Desktop/dadabac/7_BIOM_table_processing/2_BIOM_length_filtered_RSVs.biom --otu_ids_to_exclude_fp /home/mh_user/Desktop/dadabac/6_seq_stats/lengthfiltered_seqtab.fna --negate_ids_to_exclude
```

#c)Inspect the BIOM table to check if there is any taxonomic assignation to Chloroplast and Mitochondria

```
biom convert -i /home/mh_user/Desktop/dadabac/7_BIOM_table_processing/2_BIOM_length_filtered_RSVs.biom -o /home/mh_user/Desktop/dadabac/7_BIOM_table_processing/1_BIOM_tax_inspection_1.txt --to-tsv --header-key taxonomy
```

#d)Remove those ASVs assigned to Chloroplast and Mitochondria from the BIOM table

```
filter_taxa_from_otu_table.py -i /home/mh_user/Desktop/dadabac/7_BIOM_table_processing/2_BIOM_length_filtered_
```

RSVs.biom -o /home/mh_user/Desktop/dadabac/7_BIOM_table_processing/3_BIOM_without_mito_chloro.biom -n Chloroplast,Mitochondria

#e)Check that these ASVs have been correctly removed.

biom convert -i /home/mh_user/Desktop/dadabac/7_BIOM_table_processing/3_BIOM_without_mito_chloro.biom -o /home/mh_user/Desktop/dadabac/7_BIOM_table_processing/2_BIOM_tax_inspection_2.txt --to-tsv --header-key taxonomy

#f) Sort samples in the BIOM table according to a particular criterion. To do this, first generate a "sample_ID_list.txt" with the desired criterion (e.g. number of sample as well as substrate type –soil and rock).

sort_otu_table.py -i /home/mh_user/Desktop/dadabac/7_BIOM_table_processing/3_BIOM_without_mito_chloro.biom -o /home/mh_user/Desktop/dadabac/7_BIOM_table_processing/4_BIOM_sorted.biom -l /home/mh_user/Desktop/dadabac/7_BIOM_table_processing/sample_id_list.txt

#g)Add metadata to BIOM table. The mapping file is only composed of three columns (#Sample ID, Source, Time).

biom add-metadata -i /home/mh_user/Desktop/dadabac/7_BIOM_table_processing/4_BIOM_sorted.biom -o /home/mh_user/Desktop/dadabac/7_BIOM_table_processing/5_BIOM_sorted_meta.biom --sample-metadata-fp mapbac_dada2.txt

#h)Obtain a "summary report" for the BIOM table before filtering.

biom summarize-table -i /home/mh_user/Desktop/dadabac/7_BIOM_table_processing/5_BIOM_sorted_meta.biom -o /home/mh_user/Desktop/dadabac/7_BIOM_table_processing/5_BIOM_sorted_meta_summary_PREFILT.txt

#i)Remove any spurious ASVs according to Dal Grande et al. (2018, New Phytologist), which stated the following "As we did not include mock communities in the sequencing runs, we followed the general recommendation to filter the final OTUs (me: or ASVs) table by removing OTUs (me: or ASVs) that represented < 0.005% of the total read abundance on a per-sample basis and that were present in < 5% of the total samples (see also Bokulich et al., 2013; Callahan et al., 2016; Krohn et al., 2016).

filter_otus_from_otu_table.py -i /home/mh_user/Desktop/dadabac/7_BIOM_table_processing/5_BIOM_sorted_meta.biom -o /home/mh_user/Desktop/dadabac/7_BIOM_table_processing/6_BIOM_filt.biom --min_count_fraction 0.0000004167

#j)Obtain an additional summary of the post-filtered BIOM table.

biom summarize-table -i /home/mh_user/Desktop/dadabac/7_BIOM_table_processing/6_BIOM_filt.biom -o /home/mh_user/Desktop/dadabac/7_BIOM_table_processing/6_BIOM_filt_summary_POSTFILT.txt

#k)Convert the BIOM table into txt, and use Excel, to fix the text in the Nomenclature column in order that the BIOM file could be read in other programs such as Calypso.

biom convert -i /home/mh_user/Desktop/dadabac/7_BIOM_table_processing/6_BIOM_filt.biom -o /home/mh_user/Desktop/dadabac/7_BIOM_table_processing/3_BIOM_tax_inspection_3.txt --to-tsv --header-key taxonomy

#l)Save the resulting file as "4_BIOM_tax_inspection_4.txt" and then copy and paste it into the folder "7_BIOM_table_processing"

#m)Convert the txt file into BIOM again.

biom convert -i /home/mh_user/Desktop/dadabac/7_BIOM_table_processing/4_BIOM_tax_inspection_4.txt -o /home/mh_user/Desktop/dadabac/7_BIOM_table_processing/7_BIOM_good_taxonomy.biom --to-hdf5 --table-type="OTU table" --process-obs-metadata taxonomy

#n)Add again the metadata as it was lost in the previous step

biom add-metadata -i /home/mh_user/Desktop/dadabac/7_BIOM_table_processing/7_BIOM_good_taxonomy.biom -o /home/mh_user/Desktop/dadabac/7_BIOM_table_processing/8_BIOM_def.biom --sample-metadata-fp mapbac_dada2.txt

#p)Obtain a final summary of the final BIOM table and check it visually.

biom summarize-table -i /home/mh_user/Desktop/dadabac/7_BIOM_table_processing/8_BIOM_def.biom -o /home/mh_user/Desktop/dadabac/7_BIOM_table_processing/8_BIOM_def_summary.txt

##

Next steps: analyses of Alpha- and Beta-diversity. These are done identically as in the OTU analysis (operating procedure Ia) (see above)

##

IIb) Operating procedure for analyzing fungal *ITS1* data to infer ASVs using Microbiome Helper virtual box (Comeau et al., 2017) and dada2 (Callahan et al., 2016) scripts, with some custom modifications.

Date: 25-APR-2018; Author: Isaac Garrido Benavent; Software: MV Microbiome Helper amplicon v 0.3 and dada2

Primers used for amplification of the ITS region within the rDNA (Toju et al. 2012):

ITS1F_KYO2_CS1: ACACTGACGACATGGTTCTACATAGAGGAAGTAAAAGTCGTAA and ITS2_KYO2_CS2: TACGGTAGCAGAGACTTGGTCTTTYRCTRCGTTCTTCATC

Database used for taxonomic assignment: UNITE v.28.06.2017

#########

```
source /home/mh_user/anaconda2/bin/activate qiime1
```

#########Check that the pair-end data is sorted

```
cd ~/Desktop/dadafun

mkdir 1_fun_sorted_fastqs

parallel -j 20 'cat {} | paste - - - - | sort -k1,1 -t " " | tr "\t" "\n" >
1_algae_sorted_fastqs/{/.}.sorted.fastq' ::: rawdata/*.fastq
```

#########Extraction step with ITXs (Bengtsson-Palme et al., 2013) using a script made available by J.L. Darcy at GitHub (https://github.com/darcyj).

#First of all, create a new folder with just the R1 files. Name it "2_only_forward_reads". Paste these files into this.

```
mkdir 2_only_forward_reads
```

#Running Darcy's script in loop:

```
for f in 2_only_forward_reads/*_R1_*.fastq; do \

  mkdir temp_itsx \

  fastq_to_fasta.r -i $f -o temp_itsx/converted.fasta \

  ITSx -i temp_itsx/converted.fasta -o temp_itsx/itsxTemp -t "Fungi" --cpu 4 --
graphical F --preserve T --save_regions ITS1 \

  itsx_fastq_extractor.r -f temp_itsx/itsxTemp.ITS1.fasta -q $f -t 4 -o $f"_extracted.fastq"
\
```

  rm -r temp_itsx \

done

mkdir 3_ITSx_extracted #create this folder and copy here all extracted files

(optional)To conduct the extraction step per each file use:

cd 2_only_forward_reads

f="Rock1x1H_R1_all.sorted.fastq" #cada vez tengo que ir cambiando el nombre del archivo

mkdir temp_itsx

fastq_to_fasta.r -i $f -o temp_itsx/converted.fasta

ITSx -i temp_itsx/converted.fasta -o temp_itsx/itsxTemp -t "Fungi" --cpu 4 --graphical F --preserve T --save_regions ITS1

itsx_fastq_extractor.r -f temp_itsx/itsxTemp.ITS1.fasta -q $f -t 4 -o /home/mh_user/Desktop/dadafun/2_only_forward_reads/$f"_extracted.fastq"

rm -r temp_itsx

#Once all files are copied to folder "3_ITSx_extracted", remove folder "2_only_forward_reads" to free disk space.

#########Analyzing quality of reads with FastQC

mkdir 4_fastqc_out

fastqc -t 1 3_ITSx_extracted/*all.sorted.fastq_extracted.fastq -o 4_fastqc_out

#########Filter by quality

dada2_filter.R -f 3_ITSx_extracted --truncLen 100 --maxN 0 --maxEE 3 --truncQ 2 --threads 9 -o 5_dada2_filtered_fastqs --single # lo de --single es importante para indicarle que sólo el R1 se está teniendo en consideración

#########Infer Amplicon Sequence Variants (ASVs)

cd ~/Desktop/dadafun

dada2_inference.R -f 5_dada2_filtered_fastqs --single --seed 4124 -t 9 --verbose --plot_errors #the parameter --single indicates that only R1 files are being taken in consideration

#########Chimera checking and Taxonomy assignment

dada2_chimera_taxa.R -i seqtab.rds -r ~/Documents/dada2_rdp_ref/sh_general_release_dynamic_s_01.12.2017.fasta --skip_species -t 9 ##According to information available in dada2's GitHub, use the "general fasta release" of UNITE fungal database.

#########Combining logfiles to this point

merge_logfiles.R -i dada2_filter_read_counts.txt,dada2_inferred_read_counts.txt,dada2_nonchimera_counts.txt -n filter,infer,chimera -o combined_log.txt

mkdir 6_output_and_log_files

#########Convert dada2 output to BIOM and FASTA

convert_dada2_out.R -i seqtab_final.rds -b seqtab.biom -f seqtab.fasta --taxa_in tax_final.rds

#####################   EDITING THE BIOM TABLE   #################

#a)Add "taxonomy labels" as metadata to the BIOM table, and then inspect the BIOM table.

cd ~/Desktop/dadafun

mkdir 7_BIOM_table_processing #manually, copy in this folder the files named "seqtab.biom", "seqtab.fasta" and "taxa_metadata.txt"

cd ~/Desktop/dadafun/7_BIOM_table_processing

biom add-metadata -i seqtab.biom -o seqtab_tax.biom --observation-metadata-fp taxa_metadata.txt --observation-header OTUID,taxonomy --sc-separated taxonomy

biom convert -i seqtab_tax.biom -o /home/mh_user/Desktop/dadafun/7_BIOM_table_processing/1_seqtab_tax_txt.txt --to-tsv --header-key taxonomy

#In Excel, check whether there are any taxonomic assignment as: 1) Mitochondria and Chloroplast, 2)k__Metazoa, 3) k__Plantae, 4) k__Rhizaria, 5) k__Stramenopila, 6) k__Chromista, k__Alveolata, Unclassified, k__unidentified

#b) Sort samples in the BIOM table according to a particular criterion. To do this, first generate a "sample_ID_list.txt" with the desired criterion (e.g. number of sample as well as substrate type –soil and rock).

sort_otu_table.py -i seqtab_tax.biom -o /home/mh_user/Desktop/dadafun/7_BIOM_table_processing/1_BIOM_tax_sort.biom -l

/home/mh_user/Desktop/dadafun/7_BIOM_table_processing/1_samples_id_list_sorting.txt

#c)Remove any ASVs that was not classified as a fungus.

filter_taxa_from_otu_table.py -i /home/mh_user/Desktop/dadafun/7_BIOM_table_processing/1_BIOM_tax_sort.biom -o /home/mh_user/Desktop/dadafun/7_BIOM_table_processing/2_BIOM_filt1.biom -n k__Metazoa,k__Plantae,k__Rhizaria,k__Alveolata,k__Chromista,k__unidentified

#d) Add metadata to BIOM table. The mapping file is only composed of three columns (#Sample ID, Source, Time).

biom add-metadata -i /home/mh_user/Desktop/dadafun/7_BIOM_table_processing/2_BIOM_filt1.biom -o /home/mh_user/Desktop/dadafun/7_BIOM_table_processing/3_BIOM_filt1_meta.biom --sample-metadata-fp /home/mh_user/Desktop/dadafun/mapfun_dada2.txt

#e)Obtain a "summary report" for the BIOM table before filtering.

biom summarize-table -i /home/mh_user/Desktop/dadafun/7_BIOM_table_processing/3_BIOM_filt1_meta.biom -o /home/mh_user/Desktop/dadafun/7_BIOM_table_processing/2_BIOM_PREFILT.txt

#f)Remove any spurious ASVs according to Dal Grande et al. (2018, New Phytologist), which stated the following “As we did not include mock communities in the sequencing runs, we followed the general recommendation to filter the final OTUs (me: or ASVs) table by removing OTUs (me: or ASVs) that represented < 0.005% of the total read abundance on a per-sample basis and that were present in < 5% of the total samples (see also Bokulich et al., 2013; Callahan et al., 2016; Krohn et al., 2016).

filter_otus_from_otu_table.py -i /home/mh_user/Desktop/dadafun/7_BIOM_table_processing/3_BIOM_filt1_meta.biom -o /home/mh_user/Desktop/dadafun/7_BIOM_table_processing/4_BIOM_filt2_espuris.biom --min_count_fraction 0.0000004386

#g)Obtain an additional summary of the post-filtered BIOM table.

biom summarize-table -i /home/mh_user/Desktop/dadafun/7_BIOM_table_processing/4_BIOM_filt2_espuris.biom -o /home/mh_user/Desktop/dadafun/7_BIOM_table_processing/3_BIOM_POSTFILT.txt

#h)Remove samples that contain less than expected number of reads after filtering.
filter_samples_from_otu_table.py -i /home/mh_user/Desktop/dadafun/7_BIOM_table_processing/4_BIOM_filt2_espuris.biom -o /home/mh_user/Desktop/dadafun/7_BIOM_table_processing/5_BIOM_filt2_espuris_samplesOk.biom --sample_id_fp 2_remove_empty_lowcoverag_samples.txt --

negate_sample_id_fp # "2_remove_empty_lowcoverag_samples.txt" is a text file with the samples to be eliminated located in different rows

#i)Check that the previous modifications have been conducted adequately.

biom summarize-table -i /home/mh_user/Desktop/dadafun/7_BIOM_table_processing/5_BIOM_filt2_espuris_samplesOk.biom -o /home/mh_user/Desktop/dadafun/7_BIOM_table_processing/4_BIOM_samplesOk.txt

#j)Transform to txt the latest BIOM table.

biom convert -i /home/mh_user/Desktop/dadafun/7_BIOM_table_processing/5_BIOM_filt2_espuris_samplesOk.biom -o /home/mh_user/Desktop/dadafun/7_BIOM_table_processing/5_BIOM_filt2_espuris_samplesOK_txt.txt --to-tsv --header-key taxonomy

#k)Improve the taxonomic assignments of the "Unclassified" based on a GenBank-custom ITS1 database.

cd ~/Desktop/dadafun

mkdir 8_BIOM_improved_taxonomy

#ki)First, create a txt file with the ASV ID of those ASV whose taxonomic assignment may be improved (e.g. the "Unassignable"), and those with incomplete taxonomic assignments, such as "k__Fungi". Thus, the txt file would be named "1_ASVs_IDs_bad_taxonomy.txt". Save it in folder "8_BIOM_improved_taxonomy".

#kii)Execute the following command on the BIOM table named "4_BIOM_filt2_espuris.biom" (in case no samples were removed in previous steps) or table "5_BIOM_filt2_espuris_samplesOk.biom" (in case some samples were removed in previous steps).

filter_otus_from_otu_table.py -i /home/mh_user/Desktop/dadafun/7_BIOM_table_processing/5_BIOM_filt2_espuris_samplesOk.biom -o /home/mh_user/Desktop/dadafun/8_BIOM_improved_taxonomy/1_BIOM_bad_taxonomy_ASVs.biom -e /home/mh_user/Desktop/dadafun/8_BIOM_improved_taxonomy/1_ASVs_IDs_bad_taxonomy.txt --negate_ids_to_exclude

###kiii)Use the new BIOM to select the fasta files from the representative file of sequences.

filter_fasta.py -f /home/mh_user/Desktop/dadafun/7_BIOM_table_processing/seqtab.fasta -b

/home/mh_user/Desktop/dadafun/8_BIOM_improved_taxonomy/1_BIOM_bad_taxonomy_ASVs.biom -o /home/mh_user/Desktop/dadafun/8_BIOM_improved_taxonomy/1_repseq_bad_taxonomy.fna

###kiv)Assign taxonomy with SortMerna to the selected sequences.

assign_taxonomy.py -i /home/mh_user/Desktop/dadafun/8_BIOM_improved_taxonomy/1_repseq_bad_taxonomy.fna -t /home/mh_user/Desktop/Custom_databases/qiime_database_fung/Taxonomy_final.txt -r /home/mh_user/Desktop/Custom_databases/qiime_database_fung/DB.fasta -m sortmerna --sortmerna_threads 3 -o /home/mh_user/Desktop/dadafun/8_BIOM_improved_taxonomy/

###kv) Manually, in Excel, copy the Tax IDs of the ASVs whose taxonomic assignments have improved and paste it on the original BIOM ASV table, whose name is "5_BIOM_filt2_espuris_samplesOK_txt.txt", which is located in folder "7_BIOM_table_processing". Change the name of this file to "6_BIOM_impro_taxono_txt.txt ". Copy that file and paste it into folder "8_BIOM_improved_taxonomy".

#Important: Identify ASVs whose taxonomy has still a "Unclassified" label. Change this label to "aUnclassified" to remove these from the final table in the next step.

###kvi)Remove the "aUnclassified".

biom convert -i /home/mh_user/Desktop/dadafun/8_BIOM_improved_taxonomy/2_BIOM_impro_taxono_txt.txt -o /home/mh_user/Desktop/dadafun/8_BIOM_improved_taxonomy/3_BIOM_still_with_unclassi.biom --to-hdf5 --table-type="OTU table" --process-obs-metadata taxonomy

filter_taxa_from_otu_table.py -i /home/mh_user/Desktop/dadafun/8_BIOM_improved_taxonomy/3_BIOM_still_with_unclassi.biom -o /home/mh_user/Desktop/dadafun/8_BIOM_improved_taxonomy/4_BIOM_without_unclassi.biom -n aUnclassified

biom convert -i /home/mh_user/Desktop/dadafun/8_BIOM_improved_taxonomy/4_BIOM_without_unclassi.biom -o /home/mh_user/Desktop/dadafun/8_BIOM_improved_taxonomy/4_BIOM_without_unclassi_txt.txt --to-tsv --header-key taxonomy ##This command is to convert again the BIOM table in txt and check that the "aUnclassified" were correctly removed.

###kvii)Remove sample "R2_6H" because it is the product of a contamination.

filter_samples_from_otu_table.py -i /home/mh_user/Desktop/dadafun/8_BIOM_improved_taxonomy/4_BIOM_without_unclassi.biom -o /home/mh_user/Desktop/dadafun/8_BIOM_improved_taxonomy/5_BIOM_no_cont_sa

mples.biom --sample_id_fp /home/mh_user/Desktop/dadafun/8_BIOM_improved_taxonomy/3_remove_sample.txt --negate_sample_id_fp

###kviii) Add again the metadata as it was lost in the previous step. However, use a different mapping file because there were some samples removed in previous steps. The new mapping file is named "mapfun_dada2_def.txt" and it is saved in folder "8_BIOM_improved_taxonomy".

biom add-metadata -i /home/mh_user/Desktop/dadafun/8_BIOM_improved_taxonomy/5_BIOM_no_cont_samples.biom -o /home/mh_user/Desktop/dadafun/8_BIOM_improved_taxonomy/6_BIOM_def.biom --sample-metadata-fp /home/mh_user/Desktop/dadafun/8_BIOM_improved_taxonomy/mapfun_dada2_def.txt

###kix)Obtain a final summary of the final BIOM table and check it visually.

biom summarize-table -i /home/mh_user/Desktop/dadafun/8_BIOM_improved_taxonomy/6_BIOM_def.biom -o /home/mh_user/Desktop/dadafun/8_BIOM_improved_taxonomy/6_BIOM_def_summary.txt

##

Next steps: analyses of Alpha- and Beta-diversity. These are done identically as in the OTU analysis (operating procedure Ib) (see above)

##

IIc) Operating procedure for analyzing fungal *rbcL* data to infer ASVs using the Microbiome Helper virtual box (Comeau et al., 2017; https://github.com/mlangill/microbiome_helper/wiki/16S-standard-operating-180 procedure) and dada2 (Callahan et al., 2016) scripts, with some custom modifications..

Date: 26-APR-2018; Author: Isaac Garrido Benavent; Software: MV Microbiome Helper amplicon v 0.3 and dada2

Primer used for amplification of RuBisCO region (Nozaki et al. 1995):

CS1-rbcL650 (F): GTTTCCTTTTCGTAGCTGAAGC and CS2-rbcL830 (R): TTAGCTGTGAAACCACCTGTTA

#########

```
source /home/mh_user/anaconda2/bin/activate qiime1   #esto es para activar qiime; se debe hacer siempre
```

#########Check that the pair-end data is sorted

```
cd ~/Desktop/dadalga

mkdir 1_algae_sorted_fastqs

parallel -j 20 'cat {} | paste - - - - | sort -k1,1 -t " " | tr "\t" "\n" > 1_algae_sorted_fastqs/{/.}.sorted.fastq' ::: rawdata/*.fastq
```

#########Remove primer sequences

```
cd ~/Desktop/dadalga

mkdir 2_primer_trimmed_fastqs

parallel --link --jobs 9 'cutadapt --pair-filter any --no-indels --discard-untrimmed -g GTTTCCTTTTCGTAGCTGAAGC -G TTAGCTGTGAAACCACCTGTTA -o 2_primer_trimmed_fastqs/{1/}.gz -p 2_primer_trimmed_fastqs/{2/}.gz {1} {2} > 2_primer_trimmed_fastqs/{1/}_cutadapt_log.txt' ::: 1_algae_sorted_fastqs/*_R1_*.fastq ::: 1_algae_sorted_fastqs/*_R2_*.fastq
```

#to summarize the results of cutadapt in a single file

```
parse_cutadapt_logs.py -i 2_primer_trimmed_fastqs/*log.txt
```

#########Inspect quality of reads with FastQC

```
mkdir 3_primer_trimmed_fastqc_out

fastqc -t 9 2_primer_trimmed_fastqs/*fastq.gz -o 3_primer_trimmed_fastqc_out/
```

#########Filter by quality

dada2_filter.R -f 2_primer_trimmed_fastqs --trimLeft 10 --truncLen 150,120 --maxN 0 --maxEE 3,7 --truncQ 2 --threads 9 --f_match _R1_.*fastq.gz --r_match _R2_.*fastq.gz

#Important: A default folder named "filtered_fastqs" is generated with the previous command. Rename it manually to "4_filtered_fastqs".

#########Infer Amplicon Sequence Variants (ASVs)

cd ~/Desktop/dadalga

dada2_inference.R -f 4_filtered_fastqs --seed 4124 -t 9 --verbose --plot_errors

#########Chimera checking and Taxonomy assignment

dada2_chimera_taxa.R -i seqtab.rds -r ~/Documents/dada2_rdp_ref/DB.fasta --skip_species -t 9 #the database used is a custom database constructed with rbcL data downloaded from GenBank

#Important: The taxonomy is assigned in this step. However, I prefer to use the QIIME script assign_taxonomy.py. This will be done later.

#########Combining logfiles to this point

merge_logfiles.R -i cutadapt_log.txt,dada2_filter_read_counts.txt,dada2_inferred_read_counts.txt,dada2_nonchimera_counts.txt -n cutadapt,filter,infer,chimera -o combined_log.txt

mkdir 5_output_and_log_files ## Save in this folder all the log files in txt

#########Convert dada2 output to BIOM and FASTA

convert_dada2_out.R -i seqtab_final.rds -b seqtab.biom -f seqtab.fasta --taxa_in tax_final.rds

######### Remove any further sequence artifact (e.gg extremely long sequence reads) using seqkit (Shen et al. 2016).

sudo conda install -c bioconda seqkit #To install seqkit (Shen et al. 2016).

Generate some sequence statistics (e.g. length in bp) before and after trimming with seqkit.

mkdir 6_seq_stats

count_seqs.py -i seqtab.fasta -o 6_seq_stats/1_stats_original_data.txt

cat seqtab.fasta | '/home/mh_user/anaconda2/bin/seqkit' seq | '/home/mh_user/anaconda2/bin/seqkit' stats >> 6_seq_stats/2_seq_stats_previous_trimming.txt

```
cat seqtab.fasta | '/home/mh_user/anaconda2/bin/seqkit' seq -m 130 -M 180 | '/home/mh_user/anaconda2/bin/seqkit' stats >> 6_seq_stats/3_seq_stats_after_trimming.txt

'/home/mh_user/anaconda2/bin/seqkit' seq -m 130 -M 180 seqtab.fasta > 6_seq_stats/lengthfiltered_seqtab.fna

count_seqs.py -i 6_seq_stats/lengthfiltered_seqtab.fna -o 6_seq_stats/4_after_seqkit_stat_combined_seqs.txt
```

#########Assign taxonomy with QIIME, using the same methdology as in the Operating procedure Ic (i.e. clustering of OTUs at 97%).

```
mkdir 7_assigning_taxonomy

assign_taxonomy.py -i /home/mh_user/Desktop/dadalga/6_seq_stats/lengthfiltered_seqtab.fna -t /home/mh_user/Desktop/Custom_databases/qiime_database_algae/Taxonomy_final.txt -r /home/mh_user/Desktop/Custom_databases/qiime_database_algae/DB.fasta -m sortmerna --sortmerna_threads 3 -o /home/mh_user/Desktop/dadalga/7_assigning_taxonomy/
```

##################### EDITING THE BIOM TABLE #################

#a)Remove the "Unassigned" from the BIOM table named "seqtab.biom". To do this, select the "lengthfiltered_seqtab_tax_assignments.txt" file, which is localted in folder "7_assigning_taxonomy", and that corresponds with the taxonomic assignments based on SortMerna. Identify the ASV IDs corresponding with the "Unassigned", and create a txt file named "1_ASVs_to_filter_by_taxonomy.txt", and write the name of each of those ASV IDs in a separate row. Save this txt file in a new folder "8_BIOM_table_processing".

```
cd ~/Desktop/dadalga

mkdir 8_BIOM_table_processing

filter_otus_from_otu_table.py -i seqtab.biom -o /home/mh_user/Desktop/dadalga/8_BIOM_table_processing/1_BIOM_without_unassigned.biom --otu_ids_to_exclude_fp /home/mh_user/Desktop/dadalga/8_BIOM_table_processing/1_ASVs_to_filter_by_taxonomy.txt
```

#b)Remove from the BIOM table those ASVs with still too long or too short sequences.

```
biom convert -i /home/mh_user/Desktop/dadalga/8_BIOM_table_processing/1_BIOM_without_unassigned.biom -o /home/mh_user/Desktop/dadalga/8_BIOM_table_processing/1_BIOM_without_unassigned_txt.txt --to-tsv --header-key taxonomy
```

filter_otus_from_otu_table.py -i /home/mh_user/Desktop/dadalga/8_BIOM_table_processing/1_BIOM_without_unassigned.biom -o /home/mh_user/Desktop/dadalga/8_BIOM_table_processing/2_BIOM_without_unassigned_and_bad_lenghts.biom --otu_ids_to_exclude_fp /home/mh_user/Desktop/dadalga/8_BIOM_table_processing/2_ASVs_to_filter_by_length.txt

#c)Manually copy the taxonomic data from the file named "lengthfiltered_seqtab_tax_assignments.txt" to the previous BIOM table. To do this, first convert the BIOM table to txt.

biom convert -i /home/mh_user/Desktop/dadalga/8_BIOM_table_processing/2_BIOM_without_unassigned_and_bad_lenghts.biom -o /home/mh_user/Desktop/dadalga/8_BIOM_table_processing/2_BIOM_without_unassigned_and_bad_lenghts_txt.txt --to-tsv --header-key taxonomy

#d)In Excel, take this opportunity to remove any “NA” from the taxonomic assignments. Save file with the name "3_BIOM_taxonomy.txt", and transfer it to folder "8_BIOM_table_processing".

#e)Transform the latter txt file back to BIOM format.

biom convert -i /home/mh_user/Desktop/dadalga/8_BIOM_table_processing/3_BIOM_taxonomy.txt -o /home/mh_user/Desktop/dadalga/8_BIOM_table_processing/3_BIOM_taxonomy.biom --to-hdf5 --table-type="OTU table" --process-obs-metadata taxonomy

#f) Sort samples in the BIOM table according to a particular criterion. To do this, first generate a “sample_ID_list.txt” with the desired criterion (e.g. number of sample as well as substrate type –soil and rock).

sort_otu_table.py -i /home/mh_user/Desktop/dadalga/8_BIOM_table_processing/3_BIOM_taxonomy.biom -o /home/mh_user/Desktop/dadalga/8_BIOM_table_processing/4_BIOM_taxonomy_sorted.biom -l /home/mh_user/Desktop/dadalga/8_BIOM_table_processing/sample_id_list.txt

#g) Add metadata to BIOM table. The mapping file is only composed of three columns (#Sample ID, Source, Time).

biom add-metadata -i /home/mh_user/Desktop/dadalga/8_BIOM_table_processing/4_BIOM_taxonomy_sorted.biom -o /home/mh_user/Desktop/dadalga/8_BIOM_table_processing/5_BIOM_taxonomy_sorted_meta.biom --sample-metadata-fp mapalga_dada2.txt

#h)Obtain a "summary report" for the BIOM table before filtering.

```
biom summarize-table -i /home/mh_user/Desktop/dadalga/8_BIOM_table_processing/5_BIOM_taxonomy_sorted_meta.biom -o /home/mh_user/Desktop/dadalga/8_BIOM_table_processing/5_BIOM_taxonomy_sorted_meta_summary_PREFILT.txt
```

#i) Remove any spurious ASVs according to Dal Grande et al. (2018, New Phytologist), which stated the following "As we did not include mock communities in the sequencing runs, we followed the general recommendation to filter the final OTUs (me: or ASVs) table by removing OTUs (me: or ASVs) that represented < 0.005% of the total read abundance on a per-sample basis and that were present in < 5% of the total samples (see also Bokulich et al., 2013; Callahan et al., 2016; Krohn et al., 2016).

```
filter_otus_from_otu_table.py -i /home/mh_user/Desktop/dadalga/8_BIOM_table_processing/5_BIOM_taxonomy_sorted_meta.biom -o /home/mh_user/Desktop/dadalga/8_BIOM_table_processing/6_BIOM_def.biom --min_count_fraction 0.0000005
```

#j)Obtain an additional summary of the post-filtered BIOM table.

```
biom summarize-table -i /home/mh_user/Desktop/dadalga/8_BIOM_table_processing/6_BIOM_def.biom -o /home/mh_user/Desktop/dadalga/8_BIOM_table_processing/6_BIOM_def_summary_POSTFILT.txt
```

##

Next steps: analyses of Alpha- and Beta-diversity. These are done identically as in the OTU analysis (operating procedure Ic) (see above)

##

R SCRIPTS

FURTHER ANALYSES OF THE ABIOTIC VARIABLES OF SOIL, ALPHA- AND BETA-DIVERSITY AS WELL AS THE COMBINED ANALYSIS ABIOTIC-BIOTIC VARIABLES.

RStudio version R 3.5.1 "Feather Spray" in a 64-bit Windows PC

Data: a) BIOM table with taxonomic assignments

b) OTUs or ASVs in rows

c) Samples in columns

d) Taxonomy labels displaying 7 levels.

e) BIOM having metadata already

f) Three files with beta-diversity: Bray-Curtis and (un-)weigthed UNIFRAC

g) Independent file with the metadata (in case it is necessary)

Install the following R packages and functions:

```
install.packages("devtools")

devtools::install_github("biomformat", "joey711") install.packages("GGally")

install.packages("dplyr")

install.packages("tidyr")

install.packages("ggfortify")

install.packages("githubinstall")

githubinstall("ggvegan")

install.packages("betapart")

install.packages("ape")

install.packages("picante")
```

Activate libraries

```
library(biomformat)

library(GGally)

library(dplyr)

library(tidyr)

library(grid)

library(gridExtra)

library(ggplot2)

library(ggvegan)

library(vegan)

source("C:/Users/phyloramalina_post/Desktop/antarctic_glacier/R/cleanplot.pca.R")

source("C:/Users/phyloramalina_post/Desktop/antarctic_glacier/R/myplotbetadisp.R")

library(githubinstall)

library(ggfortify)

library(betapart)

library(picante)

library(ape)
```

Upload the BIOM table into R, and transform it into a data.frame

```
file_path <-
"C:/Users/phyloramalina_post/Desktop/antarctic_glacier/bacterias_97clus/data/CSS_no
rm_otu_table_met_quim.biom" ## the BIOM table is already normalized by CSS
method and included the abiotic soil variables as metadata

dat <- read_biom(file_path) # Read the BIOM file and load it to an object

otu_table_CSS <- as.data.frame(as.matrix(biom_data(dat))) # Coerce dat into a matrix
(with a lowercase ‘m’) and then into a data frame
```

Create two files from the BIOM table: one with the taxonomy and the other with the sample metadata

```
taxonomy <- observation_metadata(dat) # Get taxonomy

metadata <- sample_metadata(dat) # Get sample metadata
```

Take a look at them

```
head(otu_table_CSS[,1:15])

head(metadata[,1:11])

head(taxonomy)
```

##

##

ANALYSES OF ABIOTIC VARIABLES

##

##

1) Editing of metadata table based on the category Time (three-stage chronosequence)

#Transform to numeric the values of cuantitative variables

```
metadata$ratio_c_n <- as.numeric(metadata$ratio_c_n)

metadata$ppm_fosforo <- as.numeric(metadata$ppm_fosforo)

metadata$ppm_amonio <- as.numeric(metadata$ppm_amonio)

metadata$ppm_nitrato <- as.numeric(metadata$ppm_nitrato)

metadata$Ctotal <- as.numeric(metadata$Ctotal)

metadata$Ntotal <- as.numeric(metadata$Ntotal)

metadata$conductividad <- as.numeric(metadata$conductividad)

metadata$pH <- as.numeric(metadata$pH)

metadata$mat_org <- as.numeric(metadata$mat_org)
```

#Negative values should be transformed to absolute

```
metadata[4] <- lapply(metadata[4], abs)
```

#Remove any rows with NA

```
metadata_noNA <- filter(metadata, !is.na(ppm_nitrato))
```

#Obtain the numeric values for each variable in a sequential way according to “Time”

```
metadata_num_TIME <- select(metadata_noNA, ppm_nitrato, ppm_amonio,
ppm_fosforo, mat_org, pH, conductividad, Ntotal, Ctotal, ratio_c_n, Time)

tapply(metadata_num_TIME$ppm_nitrato, metadata_num_TIME$Time, summary)
#here change the name of the variable each time
```

#Change the name of "row.names" (e.j. st1_1, st_2, etc)

```
row.names(metadata_num_TIME) <- paste(metadata_num_TIME$Time,
row.names(metadata_num_TIME), sep="_")
```

#This is for representing visually the data in boxplots

```
var_ambient_summ <- metadata_num_TIME %>%

  group_by(Time) %>%

  gather(variable, value, ppm_nitrato:ratio_c_n) %>%

  ggplot() +

  geom_boxplot(aes(x = Time, y = value)) +

  facet_wrap(~ variable, scales = "free") +

  xlab("Succession stage") +

  ylab("")

ggsave(filename =
"C:/Users/phyloramalina_post/Desktop/antarctic_glacier/bacterias_97clus/output/env_s
ummary_graphics.pdf",

       plot = var_ambient_summ,

       device = "pdf")
```

Remove the “Time” column from metadata and create a new table that will be needed in next steps.

```
metadata_def <- select(metadata_num_TIME, ppm_nitrato, ppm_amonio, ppm_fosforo, mat_org, pH, conductividad, Ntotal, Ctotal, ratio_c_n)

str(metadata_def)

summary(metadata_def)
```

2) PCA (abiotic variables without TRANSFORMATION)

PCA based on a correlation matrix

```
env.pca <- rda(metadata_def, scale = TRUE) ## Argument scale=TRUE calls for a standardization (=normalización) of the variables

env.pca

summary(env.pca) #Default scaling 2

summary(env.pca, scaling = 1)
```

Eigenvalues

```
(ev <- env.pca$CA$eig)

# Apply Kaiser-Guttman criterion to select axes

ev[ev > mean( ev )]
```

Plot eigenvalues and % of variance for each axis

```
barplot(ev, main = "Eigenvalues for PCA on ENV", col = "bisque", las = 2)

abline(h = mean(ev), col = "red" )  #average eigenvalue

legend("topright", "Average eigenvalue", lwd = 1, col = 2, bty = "n")
```

Two PCA biplots: scaling 1 and scaling 2

Plots using cleanplot.pca()

```
par(mfrow = c(1,2))
```

```
cleanplot.pca(env.pca, point = TRUE)
```

Biplots on separate plots

Get the site scores, scaling 1, display = "wa" gives weighted average

```
par(mfrow = c(1,1))

cleanplot.pca(env.pca, point = TRUE)
```

##An further strategy for obtaining PCA

```
vf <- envfit(env.pca, scale(metadata_def), perm = 999)
```

Producing better plots step by step

```
plot(env.pca, type="n", scaling=2, xlab=("PC1 (50.65 %)"), ylab=("PC2 (15.55 %)"))

text(env.pca, display="sites", cex=1.0)

plot(vf, col=c("#EE7600"))
```

##Other high-quality PCA graphic generated with ggplot

```
pca_plot <- autoplot(prcomp(metadata_def, scale. = TRUE), data =
metadata_num_TIME, colour = 'Time', label = TRUE, label.size = 3, loadings = TRUE,
loadings.label = TRUE, loadings.label.size = 3)



ggsave(filename =
"C:/Users/phyloramalina_post/Desktop/antarctic_glacier/bacterias_97clus/output/pca_pl
ot.pdf",

       plot = pca_plot,

       device = "pdf")
```

3) Inspect abiotic variable to decide whether they need transformation.

#colnames(metadata_def) #to determine the number of variables

```
i <- 8 ## Select number, from 1 to i

ambient <- metadata_def[,i]

windows(width=12, height=12)

par(mfrow=c(3,4), mar = c(2,2,2,2), oma =c(0,0,2,0))

hist(ambient, xlab = "", col = "bisque", main = "raw data")

hist(sqrt(ambient),  xlab = "", col = "light green", main = "square root")

hist(sqrt(sqrt(ambient)),  xlab = "", col = "light green", main = "double square root")

hist(log10(ambient+1),  xlab = "", col = "light green", main = "log10")

boxplot(ambient,  xlab = "", col = "bisque", main = "raw data")

boxplot(ambient,  xlab = "", col = "light green", main = "square root")

boxplot(ambient,  xlab = "", col = "light green", main = " double square root ")

boxplot(log10(ambient),  xlab = "", col = "light green", main = "log10")

dotchart(ambient) ## Cleveland plots

dotchart(sqrt(ambient)) ## Cleveland plots

dotchart(sqrt(sqrt(ambient))) ## Cleveland plots

dotchart(log10(ambient)) ## Cleveland plots

mtext(side = 3, text = colnames(metadata_num_noNA)[i], outer = TRUE, line = 0.1,
cex = 1.3)

windows(width=12, height=12)
```

4) Result: the variables that need transformation are conductividad, mat_org, Ctotal, ratio_c_n, Ntotal.

```
metadata_def[4] <- log10(metadata_def[4]) #trans mat_org

metadata_def[6] <- log10(metadata_def[6]) #trans conductividad

metadata_def[7] <- sqrt(sqrt(metadata_def[7])) #trans Ntotal
```

```
metadata_def[8] <- sqrt(sqrt(metadata_def[8])) #trans Ctotal

metadata_def[9] <- log10(metadata_def[9]) #trans ratio_c_n

write.table(metadata_def, file = "C:/Users/phyloramalina_post/Desktop/antarctic_glacier/bacterias_97clus/output/env_transf_not_norm.txt", append = FALSE, dec = ",")
```

5) Detect correlations (multicollinearity) between transformed abiotic variables and eliminte possible correlations according to a logical criterion.

```
plot(metadata_def)

myCorr = cor(metadata_def)

myCorr



pdf("multicollinearity_plot.pdf", height = 10, width = 15)

multico_plot <- ggpairs(metadata_def)

print(multico_plot)

dev.off()
```

Use a collinearity threshold of |r| < 0.7 recommended by Dormann et al. (2013)

```
metadata_def_nocorr <- select(metadata_def, ppm_nitrato, ppm_amonio, ppm_fosforo, mat_org, pH, conductividad, ratio_c_n)
```

6) Calculate a dissimilarity matrix for the transformed abiotic variables. But, first, these have to be normalized.

```
env_norm <- wisconsin(metadata_def_nocorr) #normalization step

env_norm_bray <- vegdist(env_norm, method = "bray") #using scaled soil data

env_norm_bray



write.table(env_norm, file = "C:/Users/phyloramalina_post/Desktop/antarctic_glacier/bacterias_97clus/output/env_transf_and_norm.txt", append = FALSE, dec = ",")
```

##

###

BIOLOGICAL VARIABLES

###

###

1) Calculate a NMDS based on a BIOM table normalized by CSS. We need to transpose the OTU table so that samples = rows and OTUs = columns, because this is how the vegan package likes it:

```
t_otu_table_CSS <- as.data.frame(t(otu_table_CSS))

head(t_otu_table_CSS[,1:10])
```

Calculate the distance matrix with Bray-Curtis

```
otus_css_bray = as.matrix((vegdist(t_otu_table_CSS, "bray")))

write.table(otus_css_bray, file =
"C:/Users/phyloramalina_post/Desktop/antarctic_glacier/bacterias_97clus/output/otus_c
ss_bray.txt", append = FALSE, dec = ",")
```

Construct the NMDS. Represent it with ggplot

```
NMDS_BC = metaMDS(otus_css_bray)

NMDS_BC #entonces aparecerá el valor de stress, entre otros aspectos

MDS1_BC = NMDS_BC$points[,1]

MDS2_BC = NMDS_BC$points[,2]

NMDS_BC = data.frame(MDS1_BC = MDS1_BC, MDS2_BC = MDS2_BC, Time =
metadata$Time, Habitat = metadata$Source)

head(NMDS_BC)



# NMDS representation and save graphic

NMDS_BC_plot_source <- ggplot(NMDS_BC, aes(x=MDS1_BC, y=MDS2_BC,
col=Habitat)) +
```

```
 geom_point() +
 stat_ellipse() +
 theme_bw() +
 labs(title = "NMDS_BC_source Plot") ## plotting according to habitat ("Source")


ggsave(filename =
"C:/Users/phyloramalina_post/Desktop/antarctic_glacier/bacterias_97clus/output/NMDS_BC_Habitat.pdf",
       plot = NMDS_BC_plot_source,
       device = "pdf")


NMDS_BC_plot_time <- ggplot(NMDS_BC, aes(x=MDS1_BC, y=MDS2_BC, col=Time)) +
 geom_point() +
 stat_ellipse() +
 theme_bw() +
 labs(title = "NMDS_BC_time Plot") ## plotting according to habitat "Time"


ggsave(filename =
"C:/Users/phyloramalina_post/Desktop/antarctic_glacier/bacterias_97clus/output/NMDS_BC_Time.pdf",
       plot = NMDS_BC_plot_time,
       device = "pdf")
```

#Obtain a OTU table with just the soil samples which are the ones that have associated abiotic variables. Then, generate a Bray-Curtis dissimilarity matrix for doing analysis later.

```
t_otu_table_CSS_soil <- slice(t_otu_table_CSS, 61:120)
```

#Remove columnas in which all abundances are 0 and check it.

```
otus_css_soil_Rdef <- t_otu_table_CSS_soil[, which(colSums(t_otu_table_CSS_soil) != 0)]

str(otus_css_soil_Rdef)



write.table(otus_css_soil_Rdef, file = "C:/Users/phyloramalina_post/Desktop/antarctic_glacier/bacterias_97clus/output/otus_css_soil_Rdef.txt", append = FALSE, dec = ",")
```

Bray-Curtis

```
otus_css_soil_bray = as.matrix((vegdist(otus_css_soil_Rdef, "bray")))
```

2) betadisper test (permutation dispersion) (Anderson, Ellingsen and Mcardle 2006).

##1) Considering the samples of soil and rocks in general

```
groups_rs <- factor(c(rep(1,60), rep(2,60)), labels = c("rock","soil"))
```

Bray-Curtis distances between samples

```
dis_rs <- vegdist(t_otu_table_CSS, "bray")
```

Calculate multivariate dispersions

```
mod_rs <- betadisper(dis_rs, groups_rs)

mod_rs
```

Perform test

```
anova(mod_rs)
```

Permutation test for F

```
permutest(mod_rs, pairwise = TRUE, permutations = 99)
```

Tukey's Honest Significant Differences

```
(mod_rs.HSD <- TukeyHSD(mod_rs))
```

Plot the groups and distances to centroids on the first two PCoA axes

```
myplotbetadisper(mod_rs, ellipse = FALSE, hull = TRUE,main= "MultiVariate Permutation RS")
```

Draw a boxplot of the distances to centroid for each group

```
boxplot(mod_rs)
```

##2) Considering the three times in rocks and soil in general

```
groups_r3s3 <- factor(c(rep(1,20), rep(2,20), rep(3,20), rep(4,20), rep(5,20), rep(6,20)),
labels = c("rt1","rt2","rt3","st1","st2","st3"))

dis_r3s3 <- vegdist(t_otu_table_CSS, "bray")

mod_r3s3 <- betadisper(dis_r3s3, groups_r3s3)

mod_r3s3

anova(mod_r3s3)

permutest(mod_r3s3, pairwise = TRUE, permutations = 99)

(mod_r3s3.HSD <- TukeyHSD(mod_r3s3))

myplotbetadisper(mod_r3s3, ellipse = FALSE, hull = TRUE, main= "MultiVariate
Permutation R3S3")

boxplot(mod_r3s3)
```

3) betapart

First, create a csv file with the transposed OTU table

```
write.csv(t_otu_table_CSS, file =
"C:/Users/phyloramalina_post/Desktop/antarctic_glacier/bacterias_97clus/output/t_otu_
table_CSS.csv", append = FALSE, dec = ",")
```

```
otu_table_betapart <- read.csv(file =
"C:/Users/phyloramalina_post/Desktop/antarctic_glacier/bacterias_97clus/output/t_otu_
table_CSS_succ_stage.csv", header = TRUE, sep = ",", row.names = "site")
```

Analysis for rock samples.

```
otu_table_betapart_rock <- slice(otu_table_betapart, 1:3)

otu_table_betapart_rock_Rdef <- otu_table_betapart_rock[,
which(colSums(otu_table_betapart_rock) != 0)]
```

Analysis for soil samples.

```
otu_table_betapart_soil <- slice(otu_table_betapart, 4:6)

otu_table_betapart_soil_Rdef <- otu_table_betapart_soil[, which(colSums(otu_table_betapart_soil) != 0)]
```

Betapart for rock samples

```
otu_table_betapart_rock_Rdef[otu_table_betapart_rock_Rdef>0] <-1

rock_beta <- beta.multi(otu_table_betapart_rock_Rdef, index.family = "sor")

rock_beta

rock_beta_pair <- beta.pair(otu_table_betapart_rock_Rdef, index.family = "sor")

rock_beta_pair
```

Betapart for soil samples

```
otu_table_betapart_soil_Rdef[otu_table_betapart_soil_Rdef>0] <-1

soil_beta <- beta.multi(otu_table_betapart_soil_Rdef, index.family = "sor")

soil_beta

soil_beta_pair <- beta.pair(otu_table_betapart_soil_Rdef, index.family = "sor")

soil_beta_pair
```

2) Calculate alpha-diversity indices

```
file_path <- "C:/Users/phyloramalina_post/Desktop/antarctic_glacier/bacterias_97clus/data/otu_table_rarified.biom" ## aquí estoy incorporando la tabla BIOM rarificada pero no normalizada
```

```
dat_rare <- read_biom(file_path) # Read the BIOM file and load it to an object



otu_table_rarefied <- as.data.frame(as.matrix(biom_data(dat_rare))) # Coerce daat into a matrix (with a lowercase ‘m’) and then into a data frame
```

Transpose table

```
t_otu_table_rarefied <- as.data.frame(t(otu_table_rarefied))
```

<u>Sort rownames</u>

```
rownames_correct_order <- read.csv("C:/Users/phyloramalina_post/Desktop/antarctic_glacier/bacterias_97clus/data/rownames_correctorder.csv", header = FALSE, row.names = 1)

otu_rare_DEF <- t_otu_table_rarefied[match(rownames(rownames_correct_order), rownames(t_otu_table_rarefied)), ]
```

##############################

Evenness

##############################

```
H <- diversity(otu_rare_DEF)

J_even_table <- H/log(specnumber(otu_rare_DEF))

write.csv(J_even_table, file = "C:/Users/phyloramalina_post/Desktop/antarctic_glacier/bacterias_97clus/output/J_even_table.csv", append = FALSE, dec = ".")
```

<u>Add Source y Time factors as variables in dataframe dataframe "J_even_table". Create a new df, and change the name of variable to "Evenness"</u>

```
J_even_table_df <- as.data.frame(J_even_table)

J_even_table_df$Source <- as.factor(c(rep('rock',60), rep('soil',60)))
```

```
J_even_table_df$Time <- as.factor(c(rep('rt1',20), rep('rt2',20), rep('rt3',20), rep('st1',20), rep('st2',20), rep('st3',20)))

colnames(J_even_table_df)[1] <- "Evenness"



evenness_plot <- J_even_table_df %>%

  group_by(Time) %>%

  gather(variable, value, Evenness) %>%

  ggplot() +

  geom_boxplot(aes(x = Time, y = value)) +

  facet_wrap(~ variable, scales = "free") +

  xlab("Succession stage") +

  ylab("")



ggsave(filename = "C:/Users/phyloramalina_post/Desktop/antarctic_glacier/bacterias_97clus/output/evenness_plot_bac_clus97.pdf",

       plot = evenness_plot,

       device = "pdf")
```

```
##############################

##  Phylogenetic FAITH'S D ##

##############################
```

```
tree_bac_clus97 <- read.tree("C:/Users/phyloramalina_post/Desktop/antarctic_glacier/bacterias_97clus/data/bac_clus97_phylo_tree_DEF.tre")

Faith_pd_bac_clus97 <- pd(otu_rare_DEF, tree_bac_clus97, include.root = F)
```

```
write.csv(Faith_pd_bac_clus97, file = "C:/Users/phyloramalina_post/Desktop/antarctic_glacier/bacterias_97clus/output/Faith_pd_bac_clus97.csv", append = FALSE, dec = ".")
```

Add Source y Time factors as variables in dataframe "Faith_pd_bac_clus97". Create a new df, and change the variable's name to "Faiths_PD"

```
Faith_pd_bac_clus97_df <- as.data.frame(Faith_pd_bac_clus97)

Faith_pd_bac_clus97_df$Source <- as.factor(c(rep('rock',60), rep('soil',60)))

Faith_pd_bac_clus97_df$Time <- as.factor(c(rep('rt1',20), rep('rt2',20), rep('rt3',20), rep('st1',20), rep('st2',20), rep('st3',20)))

colnames(Faith_pd_bac_clus97_df)[1] <- "Faiths_PD"
```

#Plot according to Time

```
Faiths_PD_plot <- Faith_pd_bac_clus97_df %>%

  group_by(Time) %>%

  gather(variable, value, Faiths_PD) %>%

  ggplot() +

  geom_boxplot(aes(x = Time, y = value)) +

  facet_wrap(~ variable, scales = "free") +

  xlab("Succession stage") +

  ylab("")



ggsave(filename = "C:/Users/phyloramalina_post/Desktop/antarctic_glacier/bacterias_97clus/output/Faiths_pd_plot_time_bac_clus97.pdf",

       plot = Faiths_PD_plot,

       device = "pdf")
```

3) Import to R distance matrices for la UNWEIGHTED/WEIGHTED UNIFRAC and generate NMDS

Import distance matrices

```
unw_unifra <- read.table(file =
"C:/Users/phyloramalina_post/Desktop/antarctic_glacier/bacterias_97clus/data/unweighted_unifrac_CSS_normalized_otu_table.txt", header = TRUE, row.names = 1, dec = ".",
sep = "\t" )

w_unifra <- read.table(file =
"C:/Users/phyloramalina_post/Desktop/antarctic_glacier/bacterias_97clus/data/weighted_unifrac_CSS_normalized_otu_table.txt", header = TRUE, row.names = 1, dec = ".",
sep = "\t" )
```

Transform to "dist" objects

```
unw_unifra_def <- as.dist(as(unw_unifra, "matrix"))

w_unifra_def <- as.dist(as(w_unifra, "matrix"))
```

Calculate and represent UNWEIGHTED NMDS per Source and Time

```
NMDS_unw_unifr = metaMDS(unw_unifra_def)

NMDS_unw_unifr

MDS1_unw_unifr = NMDS_unw_unifr$points[,1]

MDS2_unw_unifr = NMDS_unw_unifr$points[,2]

NMDS_unw_unifr = data.frame(MDS1_unw_unifr = MDS1_unw_unifr,
MDS2_unw_unifr = MDS2_unw_unifr, Time = Faith_pd_bac_clus97_df$Time, Habitat
= Faith_pd_bac_clus97_df$Source)

NMDS_unw_unifr_plot_source <- ggplot(NMDS_unw_unifr, aes(x=MDS1_unw_unifr,
y=MDS2_unw_unifr, col=Habitat)) +

 geom_point() +

 stat_ellipse() +

 theme_bw() +

 labs(title = "NMDS_unw_unifr_source Plot") ## Plotting according to habitat (source)

ggsave(filename =
"C:/Users/phyloramalina_post/Desktop/antarctic_glacier/bacterias_97clus/output/NMDS_unw_unifrac_Habitat_bac_clus97.pdf",
```

```
      plot = NMDS_unw_unifr_plot_source,

      device = "pdf")

NMDS_unw_unifr_plot_time <- ggplot(NMDS_unw_unifr, aes(x=MDS1_unw_unifr, y=MDS2_unw_unifr, col=Time)) +

 geom_point() +

 stat_ellipse() +

 theme_bw() +

 labs(title = "NMDS_unw_unifr_time Plot") ## Plotting according to Time

ggsave(filename = "C:/Users/phyloramalina_post/Desktop/antarctic_glacier/bacterias_97clus/output/NMDS_unw_unifrac_Time_bac_clus97.pdf",

      plot = NMDS_unw_unifr_plot_time,

      device = "pdf")
```

Calculate and represent WEIGHTED NMDS per Source and Time

```
NMDS_w_unifr = metaMDS(w_unifra_def)

NMDS_w_unifr

MDS1_w_unifr = NMDS_w_unifr$points[,1]

MDS2_w_unifr = NMDS_w_unifr$points[,2]

NMDS_w_unifr = data.frame(MDS1_w_unifr = MDS1_w_unifr, MDS2_w_unifr = MDS2_w_unifr, Time = Faith_pd_bac_clus97_df$Time, Habitat = Faith_pd_bac_clus97_df$Source)

NMDS_w_unifr_plot_source <- ggplot(NMDS_w_unifr, aes(x=MDS1_w_unifr, y=MDS2_w_unifr, col=Habitat)) +

 geom_point() +

 stat_ellipse() +

 theme_bw() +

 labs(title = "NMDS_w_unifr_source Plot") ## Plotting according to habitat (source)
```

```
ggsave(filename =
"C:/Users/phyloramalina_post/Desktop/antarctic_glacier/bacterias_97clus/output/NMDS_w_unifrac_Habitat_bac_clus97.pdf",

       plot = NMDS_w_unifr_plot_source,

       device = "pdf")

NMDS_w_unifr_plot_time <- ggplot(NMDS_w_unifr, aes(x=MDS1_w_unifr,
y=MDS2_w_unifr, col=Time)) +

 geom_point() +

 stat_ellipse() +

 theme_bw() +

 labs(title = "NMDS_w_unifr_time Plot") ## Plotting according to Time

ggsave(filename =
"C:/Users/phyloramalina_post/Desktop/antarctic_glacier/bacterias_97clus/output/NMDS_w_unifrac_Time_bac_clus97.pdf",

       plot = NMDS_w_unifr_plot_time,

       device = "pdf")
```

##

##

Combined analysis of biotic and abiotic variables

##

##

1) Mantel test

```
bray.mantel<-mantel(otus_css_soil_bray, env_norm_bray, method = "spearman", permutations = 999)

bray.mantel
```

2) BIOENV analysis

```
res <- bioenv(otus_css_soil_Rdef, metadata_def_nocorr, method = "spearman", index = "bray", upto = ncol(metadata_def_nocorr), trace = FALSE, partial = NULL,  metric = "euclidean")

res

summary(res)
```

3) dbRDA

```
dbrda_sqrt = dbrda(otus_css_soil_Rdef ~ pH + mat_org + ratio_c_n, metadata_def_nocorr, dist = "bray", sqrt.dist = TRUE)

dbrda_sqrt

plot(dbrda_sqrt)
```

Test axes for significance

```
anova(dbrda_sqrt, by = "axis", perm.max = 500)
```

#test for sig. environ. variables

```
anova(dbrda_sqrt, by = "terms", permutations = 500)
```

To see scores

```
scor = scores(dbrda_sqrt, display=c("sp", "cn", "bp"), scaling=2)
```

4) "Variance Partitioning".

```
otu_env_varpart <- varpart(vegdist(otus_css_soil_Rdef), ~ pH, ~ mat_org, ~ ratio_c_n,
data = metadata_def_nocorr)

otu_env_varpart

showvarparts(3, bg = c("hotpink","skyblue","yellow"))

plot(otu_env_varpart, bg = c("hotpink","skyblue","yellow"))
```

Test fraction pH using dbRDA and anova.

```
phFrac <- dbrda(otus_css_soil_Rdef ~ pH, metadata_def_nocorr, dist = "bray", sqrt.dist
= TRUE)

phFrac

anova.cca(phFrac)
```

Test fraction mat_org using dbRDA and anova

```
matorgFrac <- dbrda(otus_css_soil_Rdef ~ mat_org, metadata_def_nocorr, dist =
"bray", sqrt.dist = TRUE)

matorgFrac

anova.cca(matorgFrac)
```

Test fraction ratio_c_n using dbRDA and anova

```
ratiocnFrac <- dbrda(otus_css_soil_Rdef ~ ratio_c_n, metadata_def_nocorr, dist =
"bray", sqrt.dist = TRUE)

ratiocnFrac

anova.cca(ratiocnFrac)
```
