# Supplementary material for: Differential Colonization and Succession of Microbial Communities in Rock and Soil Substrates on a Maritime Antarctic Glacier Forefield
Source: Front Microbiol. 2020 Feb 7;11:126. doi: 10.3389/fmicb.2020.00126 (PMC7018881; doi:10.3389/fmicb.2020.00126)
Supplement: Supplementary file 3 [file Image_2.PDF]

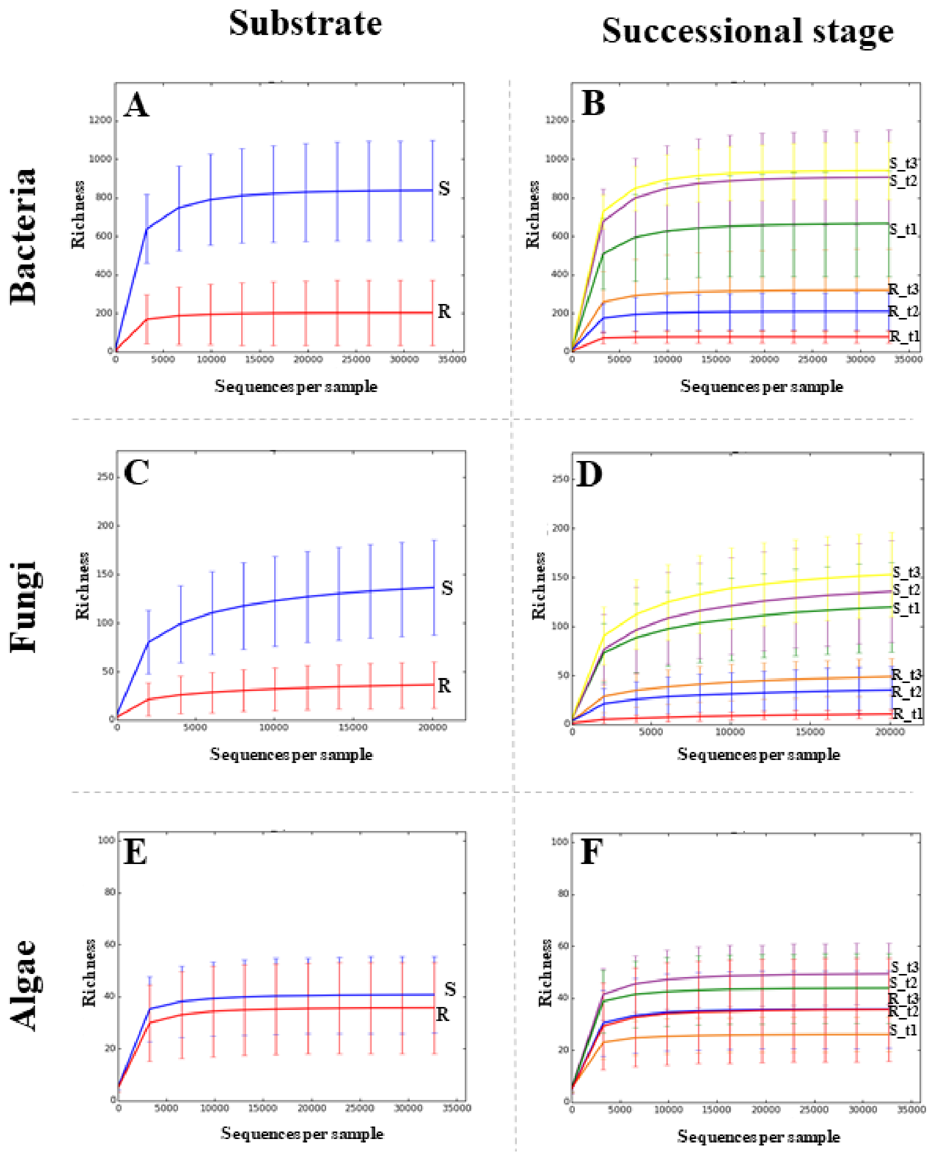

Supplementary Figure S2. Rarefaction curves for bacterial, fungal and alga ASV data and considering substrate type (rocks, R; soil, S) and successional stage.
