# Supplementary figures and images for: Differential Colonization and Succession of Microbial Communities in Rock and Soil Substrates on a Maritime Antarctic Glacier Forefield
Source: Front Microbiol. 2020 Feb 7;11:126. doi: 10.3389/fmicb.2020.00126 (PMC7018881; doi:10.3389/fmicb.2020.00126)

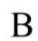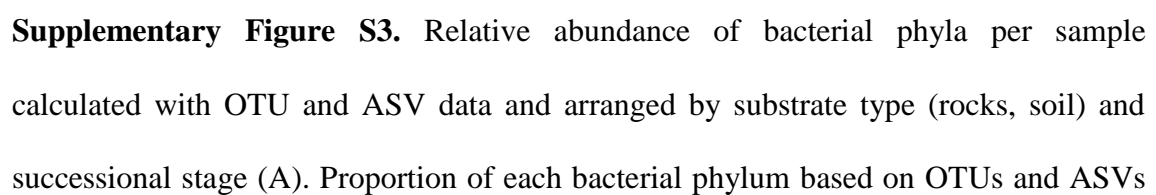

for the entire chronosequence, and considering the two substrate types independently

(B).

Supplement: Supplementary file 4 [file Image_3.PDF]
