# Supplementary material for: Differential Colonization and Succession of Microbial Communities in Rock and Soil Substrates on a Maritime Antarctic Glacier Forefield
Source: Front Microbiol. 2020 Feb 7;11:126. doi: 10.3389/fmicb.2020.00126 (PMC7018881; doi:10.3389/fmicb.2020.00126)
Supplement: Supplementary file 5 [file Image_4.PDF]

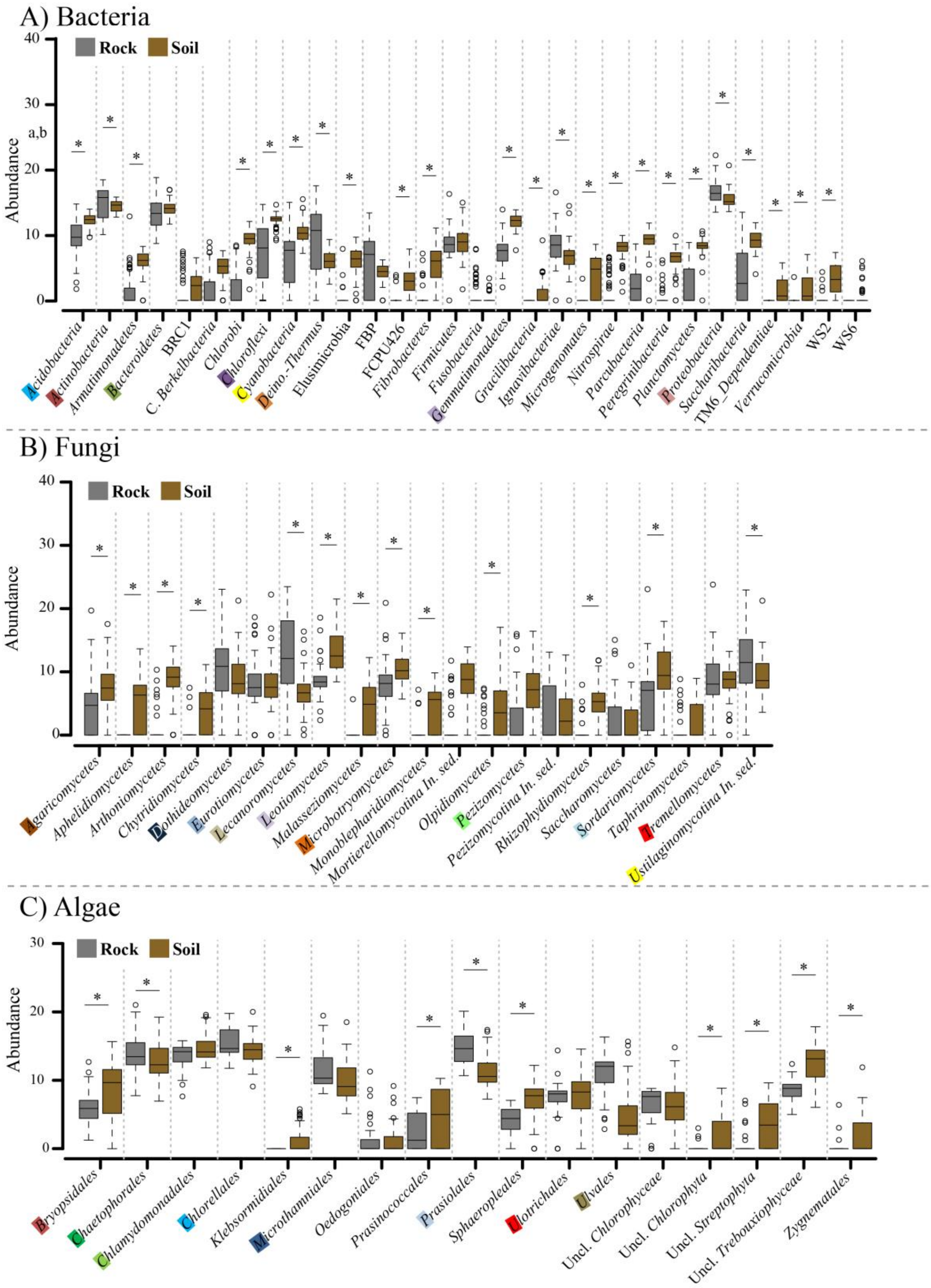

Supplementary Figure S4. Differential abundance of bacterial phyla (A), fungal classes (B) and algal orders (C) in the two substrates (rocks, soils) based on OTU tables normalized by the cumulative sum scaling (CSS) method. Asterisks indicate significant

support for differential abundances based on both the non-parametric Wilcoxon-rank test and DEseq2 implemented in Calypso v.8.56. In the latter analysis, we considered the Bonferroni-adjusted p-values. Color codes for particular bacterial, fungal and algal taxa (first letter) are the same to those used in figures depicting relative abundances in either the main manuscript or supplementary material. “C.” refers to “Candidatus”, and “Uncl.” to “Unclassified”.
