# Supplementary material for: Differential Colonization and Succession of Microbial Communities in Rock and Soil Substrates on a Maritime Antarctic Glacier Forefield
Source: Front Microbiol. 2020 Feb 7;11:126. doi: 10.3389/fmicb.2020.00126 (PMC7018881; doi:10.3389/fmicb.2020.00126)
Supplement: Supplementary file 6 [file Image_5.PDF]

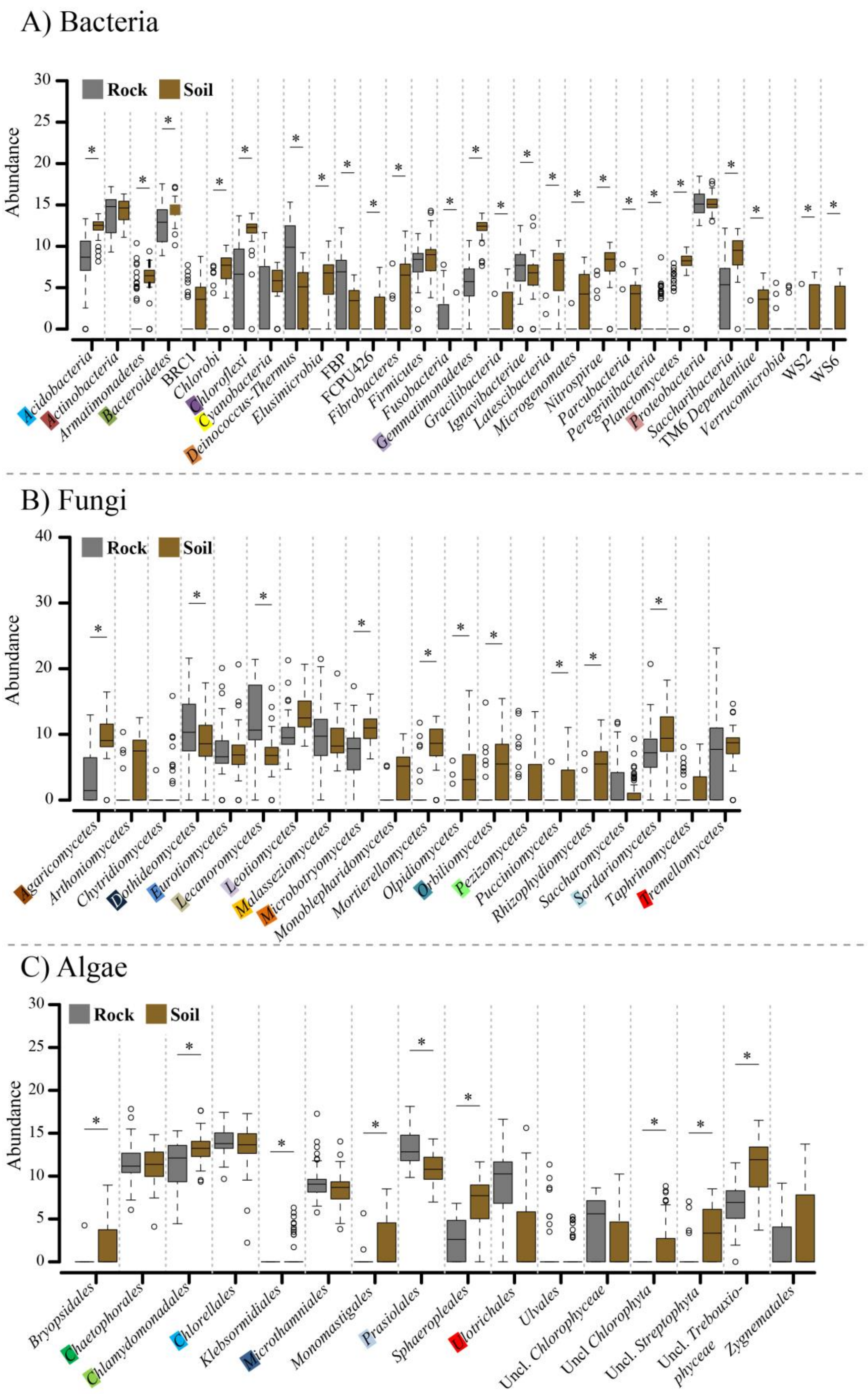
A) Bacteria
Rock
Soil
Abundance
0
5
10
15
20
25
30
Acidobacteria
Actinobacteria
Armatimonadetes
Bacteroidetes
BRC1
Chlorobi
Chloroflexi
Cyanobacteria
Deinococcus-Thermus
Elusimicrobia
FBP
FCPU426
Fibrobacteres
Firmicutes
Fusobacteria
Gemmatimonadetes
Gracilibacteria
Ignavibacteriae
Latescibacteria
Microgenomates
Nitrospirae
Parcubacteria
Peregrinibacteria
Planctomycetes
Proteobacteria
Saccharibacteria
TM6 Dependentiae
Verrucomicrobia
WS2
WS6
B) Fungi
Rock
Soil
Abundance
0
10
20
30
40
Agaricomycetes
Arthoniomycetes
Chytridiomycetes
Dothideomycetes
Eurotiomycetes
Lecanoromycetes
Leotiomycetes
Malasseziomycetes
Microbotryomycetes
Monoblepharidomycetes
Mortierellomycetes
Olpidiomycetes
Orbiliomycetes
Pezizomycetes
Pucciniomycetes
Rhizophydiomycetes
Saccharomycetes
Sordariomycetes
Taphrinomycetes
Tremellomycetes
C) Algae
Rock
Soil
Abundance
0
5
10
15
20
25
30
Bryopsidales
Chaetophorales
Chlamydomonadales
Chlorellales
Klebsormidiales
Microthamniales
Monomastigales
Prasiolales
Sphaeropleales
Ulotrichales
Ulvales
Uncl. Chlorophyceae
Uncl Chlorophyta
Uncl. Streptophyta
Uncl. Trebouxio-phyceae
Zygnematales

Supplementary Figure S5. Differential abundance of bacterial phyla (A), fungal classes (B) and algal orders (C) in the two substrates (rocks, soils) based on ASV tables normalized by the cumulative sum scaling (CSS) method. Asterisks indicate significant support for differential abundances based on both the non-parametric Wilcoxon-rank test and DEseq2 implemented in Calypso v.8.56. In the latter analysis, we considered the Bonferroni-adjusted p-values. Color codes for particular bacterial, fungal and algal taxa (first letter) are the same to those used in figures depicting relative abundances in either the main manuscript or supplementary material. “C.” refers to “Candidatus”, and “Uncl.” to “Unclassified”.
