# Supplementary material for: Differential Colonization and Succession of Microbial Communities in Rock and Soil Substrates on a Maritime Antarctic Glacier Forefield
Source: Front Microbiol. 2020 Feb 7;11:126. doi: 10.3389/fmicb.2020.00126 (PMC7018881; doi:10.3389/fmicb.2020.00126)
Supplement: Supplementary file 8 [file Image_7.PDF]

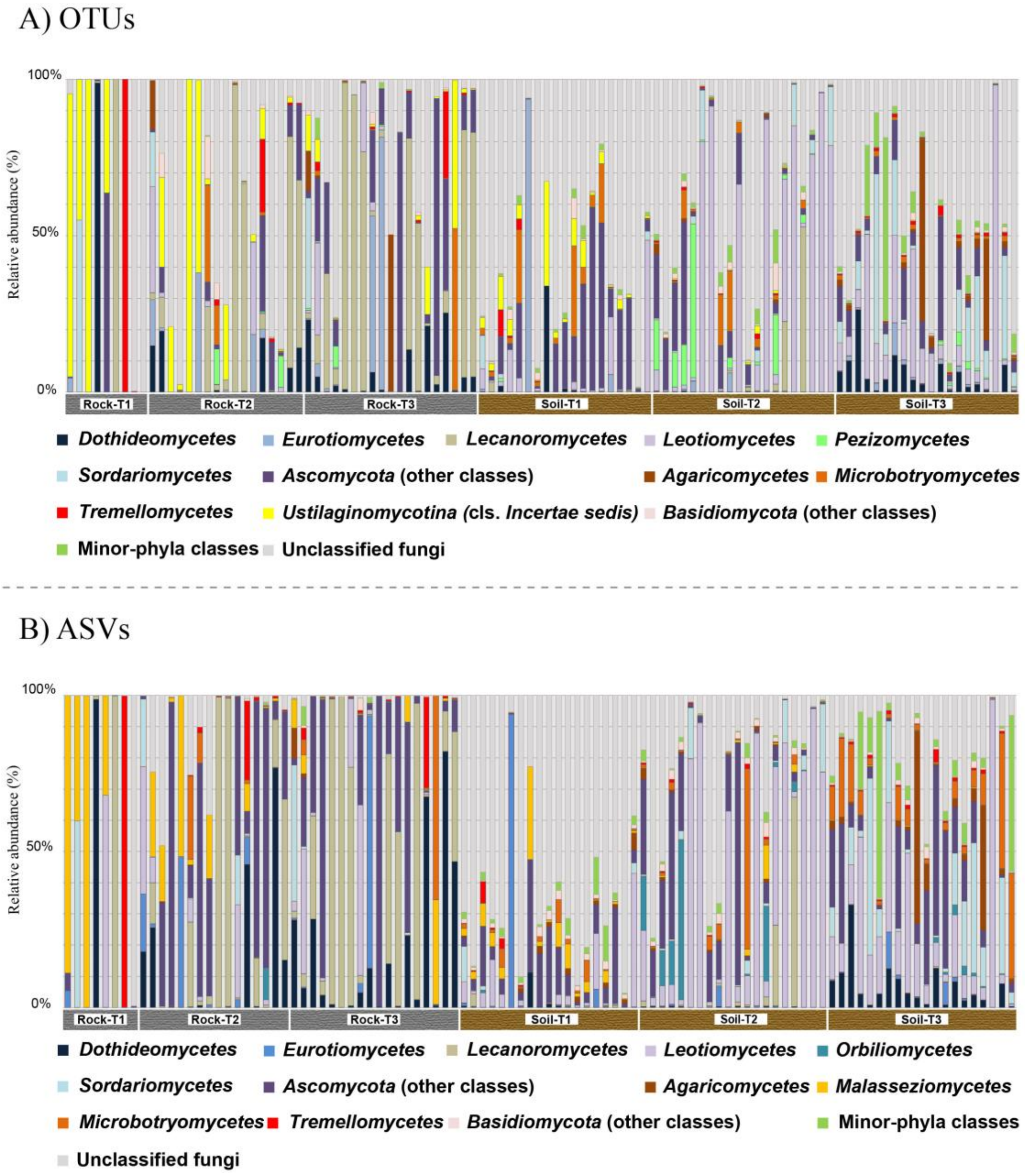

Supplementary Figure S7. Relative abundance of fungal classes per sample calculated with OTU (A) and ASV (B) data and arranged by substrate type (rocks, soil) and successional stage.
