# Supplementary material for: Differential Colonization and Succession of Microbial Communities in Rock and Soil Substrates on a Maritime Antarctic Glacier Forefield
Source: Front Microbiol. 2020 Feb 7;11:126. doi: 10.3389/fmicb.2020.00126 (PMC7018881; doi:10.3389/fmicb.2020.00126)
Supplement: Supplementary file 9 [file Image_8.PDF]

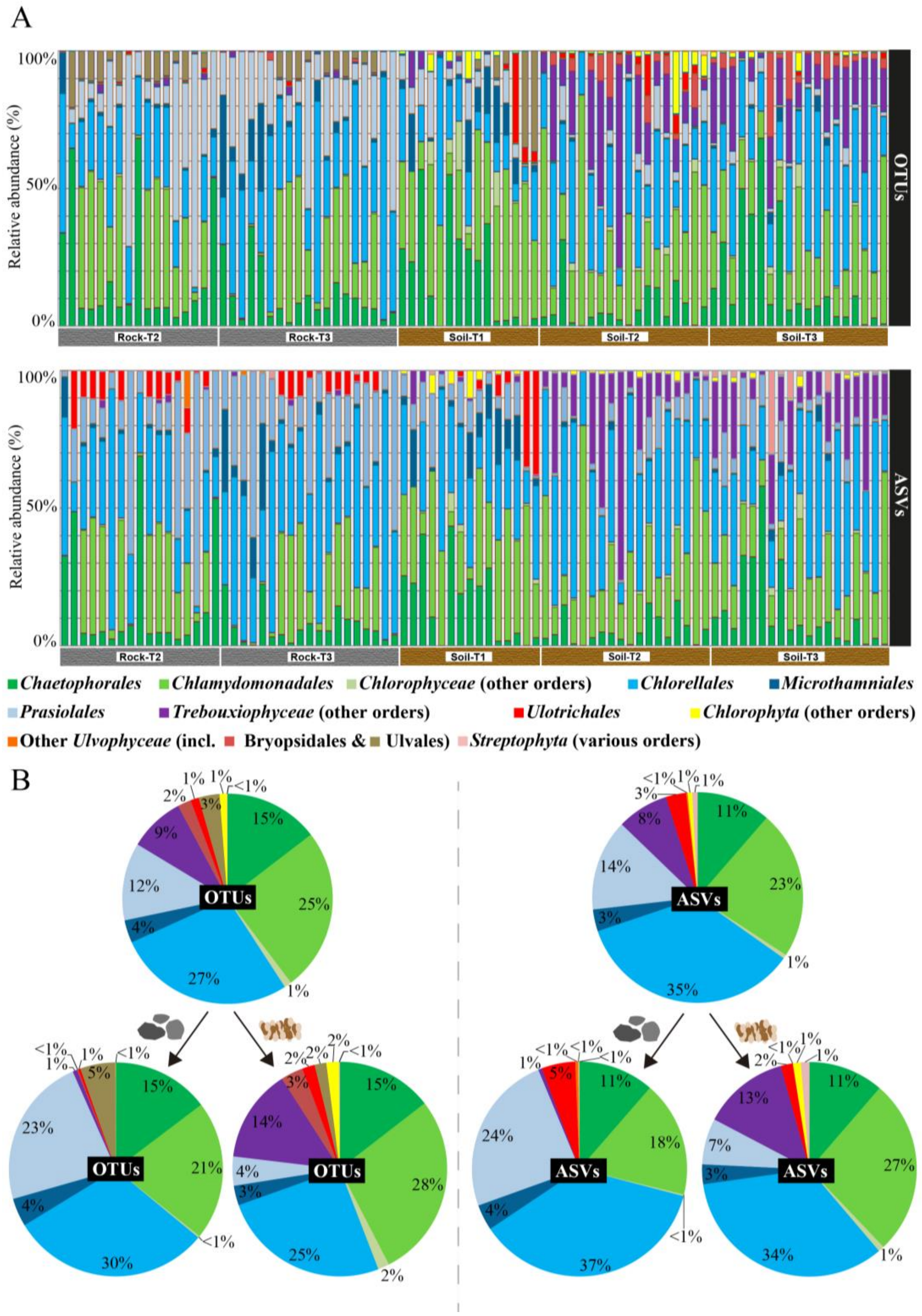

Supplementary Figure S8. Relative abundance of algal orders per sample calculated with OTU and ASV data and arranged by substrate type (rocks, soil) and successional stage (A). Proportion of each algal order based on OTUs and ASVs for the entire chronosequence, and considering the two substrate types independently (B).
