# Supplementary material for: Differential Colonization and Succession of Microbial Communities in Rock and Soil Substrates on a Maritime Antarctic Glacier Forefield
Source: Front Microbiol. 2020 Feb 7;11:126. doi: 10.3389/fmicb.2020.00126 (PMC7018881; doi:10.3389/fmicb.2020.00126)
Supplement: Supplementary file 10 [file Image_9.PDF]

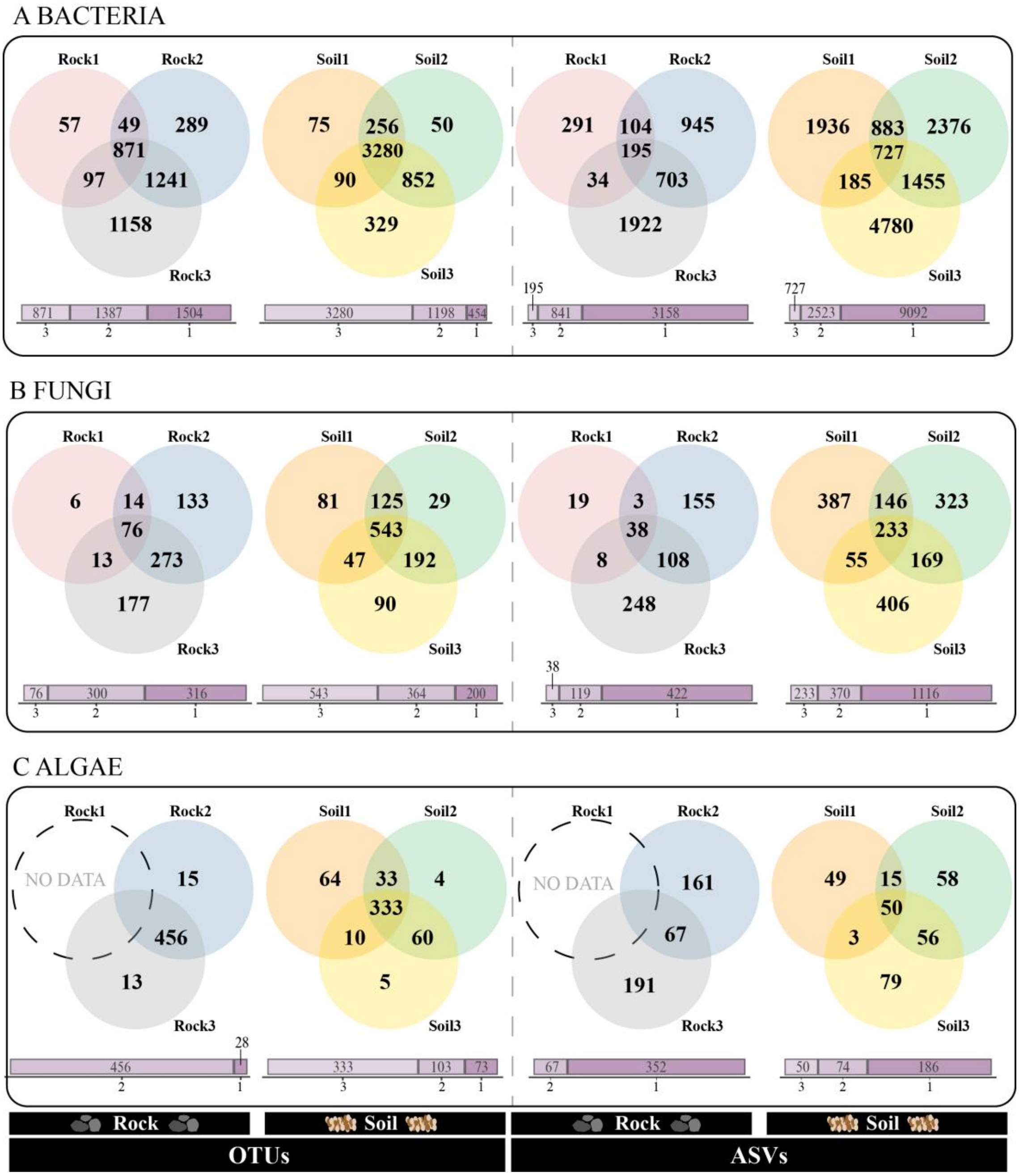

Supplementary Figure S9. Venn diagrams depicting the number of OTUs (left panel) and ASVs (right panel) unique or shared among the three successional stages for rock and soil chronosequences of bacteria (A), fungi (B), and algae (C). Lower horizontal bars indicate the number of OTUs or ASVs that are specific to just one successional stage (number 1) or shared by 2 or 3 stages.
