# Supplementary material for: Differential Colonization and Succession of Microbial Communities in Rock and Soil Substrates on a Maritime Antarctic Glacier Forefield
Source: Front Microbiol. 2020 Feb 7;11:126. doi: 10.3389/fmicb.2020.00126 (PMC7018881; doi:10.3389/fmicb.2020.00126)
Supplement: Supplementary file 11 [file Image_10.PDF]

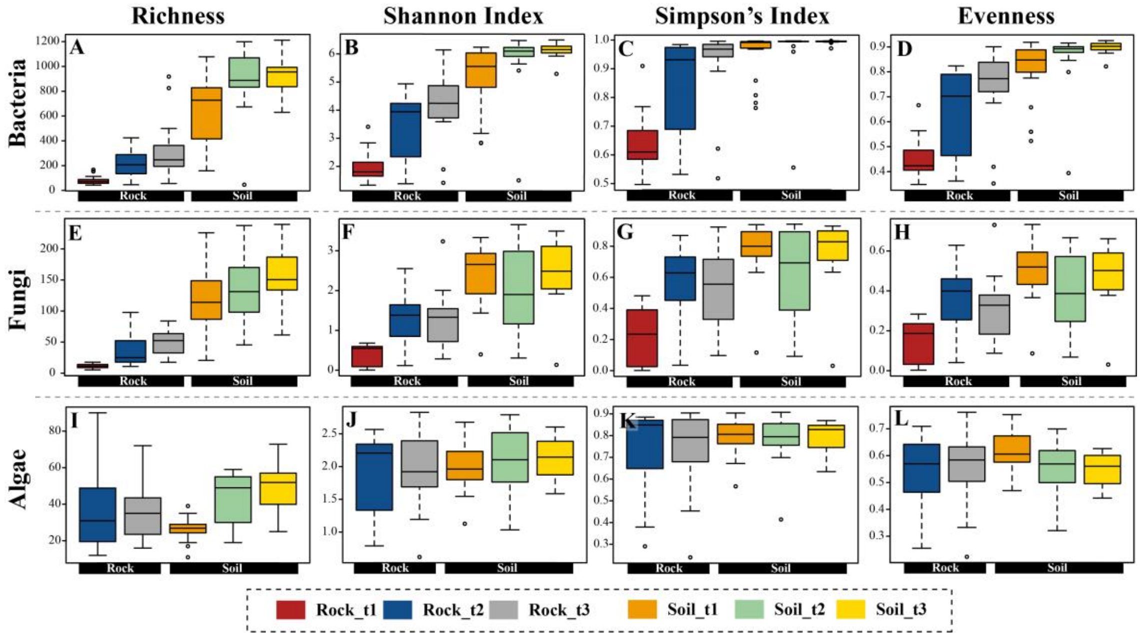

Supplementary Figure S10. Boxplots representing four alpha-diversity estimators (richness, Shannon and Simpson indices, and evenness) for bacteria, fungi and algae calculated with ASV data, and arranged according to substrate type (rocks, soil) and successional stage.
