# Supplementary material for: Differential Colonization and Succession of Microbial Communities in Rock and Soil Substrates on a Maritime Antarctic Glacier Forefield
Source: Front Microbiol. 2020 Feb 7;11:126. doi: 10.3389/fmicb.2020.00126 (PMC7018881; doi:10.3389/fmicb.2020.00126)
Supplement: Supplementary file 12 [file Image_11.PDF]

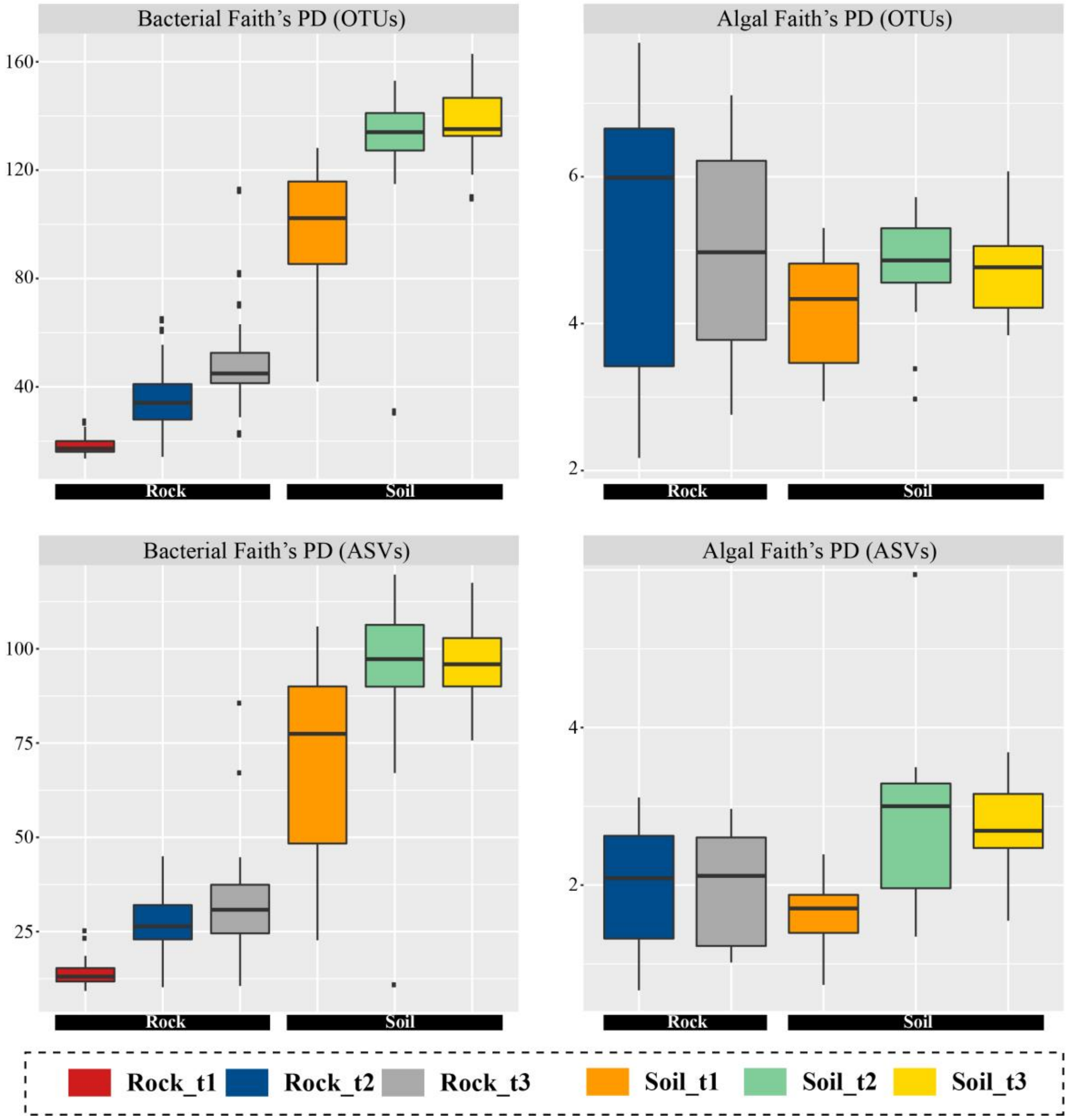

Supplementary Figure S11. Boxplots representing bacterial and algal Faith's phylogenetic diversity (PD) calculated with OTU (left panel) and ASV (right panel) data, and arranged according to substrate type (rocks, soil) and successional stage. These estimators were not computed for fungi because the high number of polymorphisms in ITS sequences precluded building a reliable alignment.
