# Supplementary material for: Differential Colonization and Succession of Microbial Communities in Rock and Soil Substrates on a Maritime Antarctic Glacier Forefield
Source: Front Microbiol. 2020 Feb 7;11:126. doi: 10.3389/fmicb.2020.00126 (PMC7018881; doi:10.3389/fmicb.2020.00126)
Supplement: Supplementary file 13 [file Image_12.PDF]

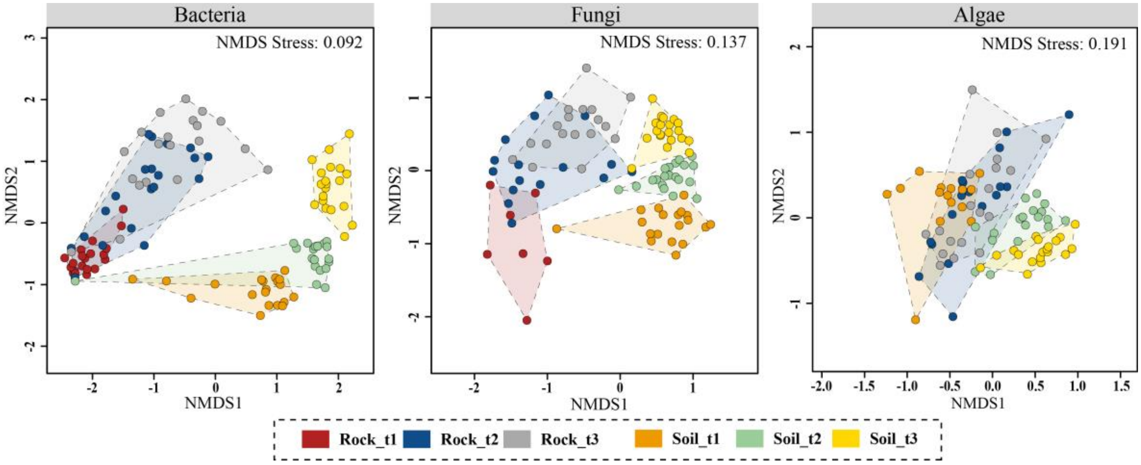

Supplementary Figure S12. Nonmetric multidimensional scaling (NMDS) ordination plots of Bray-Curtis dissimilarities for bacterial, fungal and algal communities across sample categories (i.e. substrate type plus successional stage). Analyses were based on ASV data matrices.
