# Supplementary material for: Differential Colonization and Succession of Microbial Communities in Rock and Soil Substrates on a Maritime Antarctic Glacier Forefield
Source: Front Microbiol. 2020 Feb 7;11:126. doi: 10.3389/fmicb.2020.00126 (PMC7018881; doi:10.3389/fmicb.2020.00126)
Supplement: Supplementary file 14 [file Image_13.PDF]

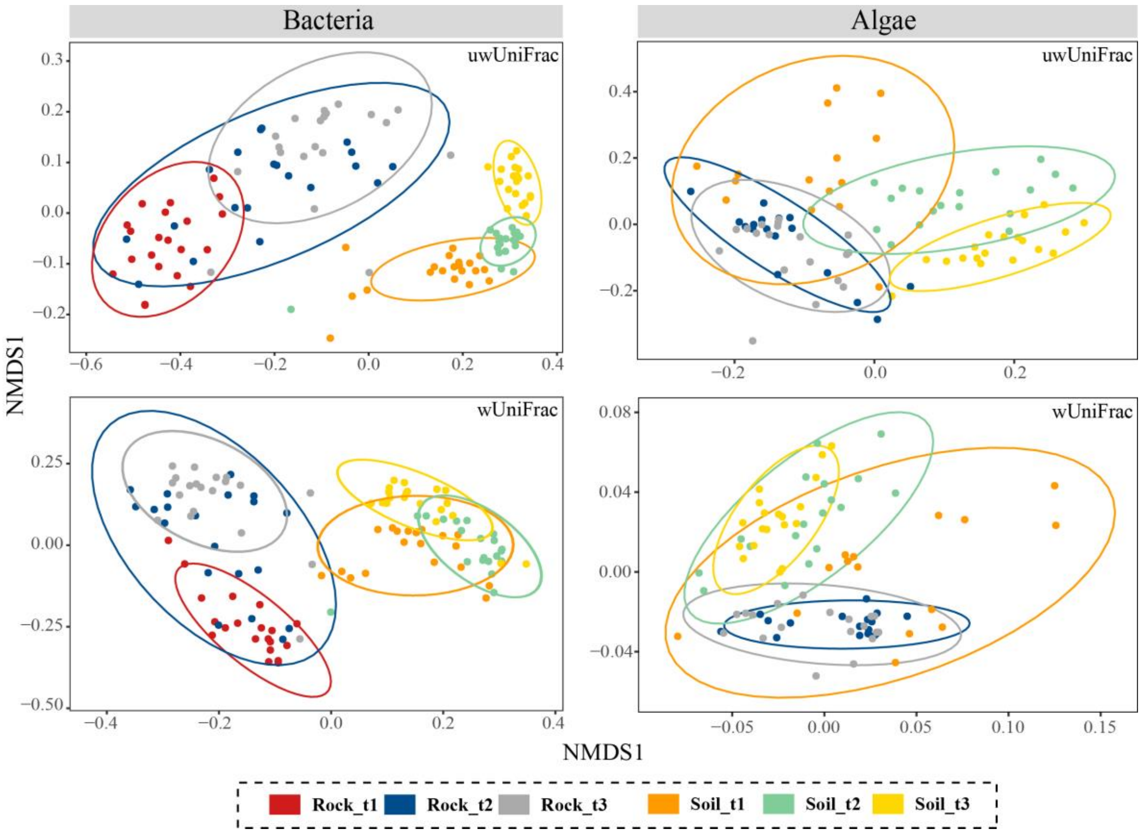

Supplementary Figure S13. Nonmetric multidimensional scaling (NMDS) ordination plots of unweigthed (upper panel) and weighted (lower panel) UniFrac distances for bacterial and algal communities across sample categories (i.e. substrate type plus successional stage). Analyses were based on OTU data matrices.
