# Supplementary material for: Differential Colonization and Succession of Microbial Communities in Rock and Soil Substrates on a Maritime Antarctic Glacier Forefield
Source: Front Microbiol. 2020 Feb 7;11:126. doi: 10.3389/fmicb.2020.00126 (PMC7018881; doi:10.3389/fmicb.2020.00126)
Supplement: Supplementary file 15 [file Image_14.PDF]

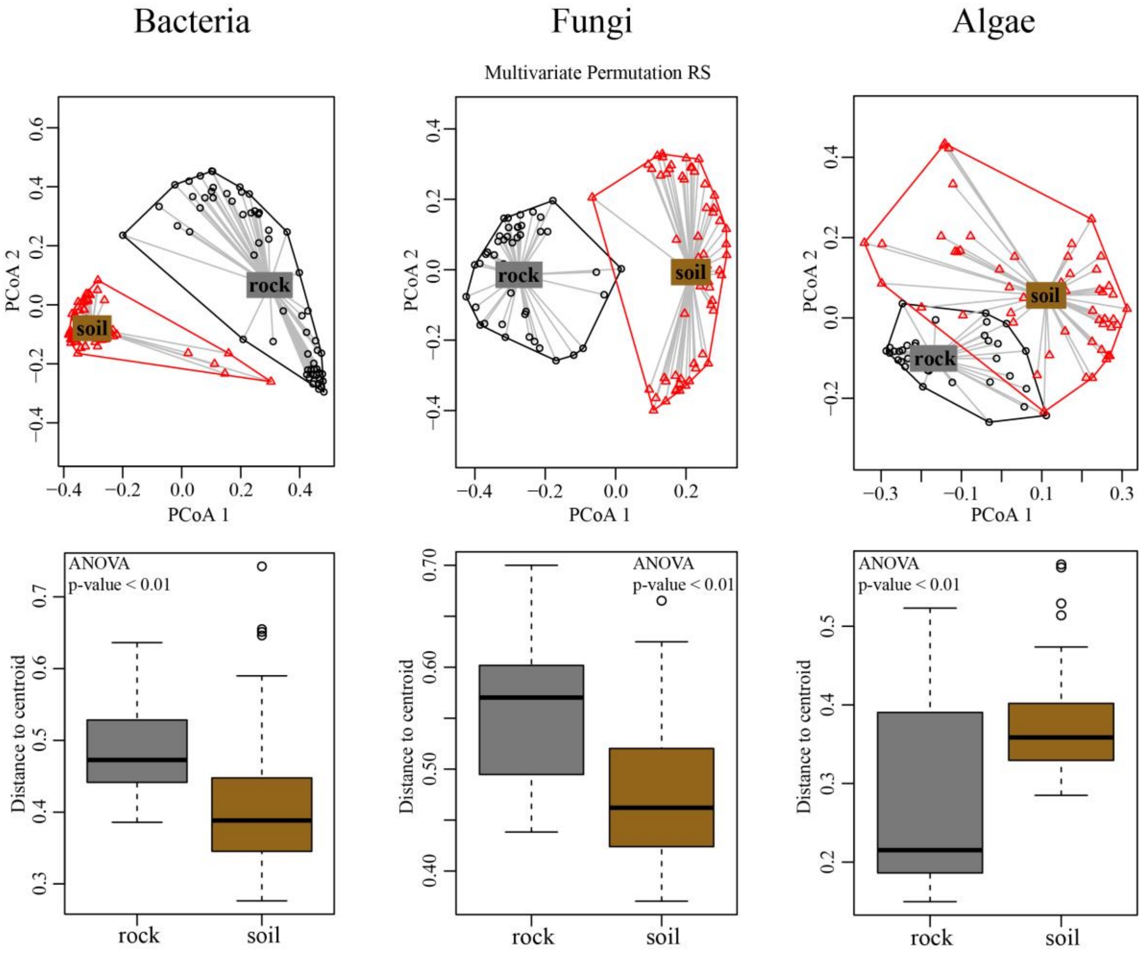

Supplementary Figure S14. Principal Coordinates Analysis (PCoA) plots of Bray-Curtis dissimilarities representing the graphical results of multivariate homogeneity tests that compare sample dispersion between substrate types (rocks, soil) in bacterial, fungal and algal communities. Boxplots in the lower panel depict basic summary data related to distances to centroids.
