# Supplementary material for: Differential Colonization and Succession of Microbial Communities in Rock and Soil Substrates on a Maritime Antarctic Glacier Forefield
Source: Front Microbiol. 2020 Feb 7;11:126. doi: 10.3389/fmicb.2020.00126 (PMC7018881; doi:10.3389/fmicb.2020.00126)
Supplement: Supplementary file 16 [file Image_15.PDF]

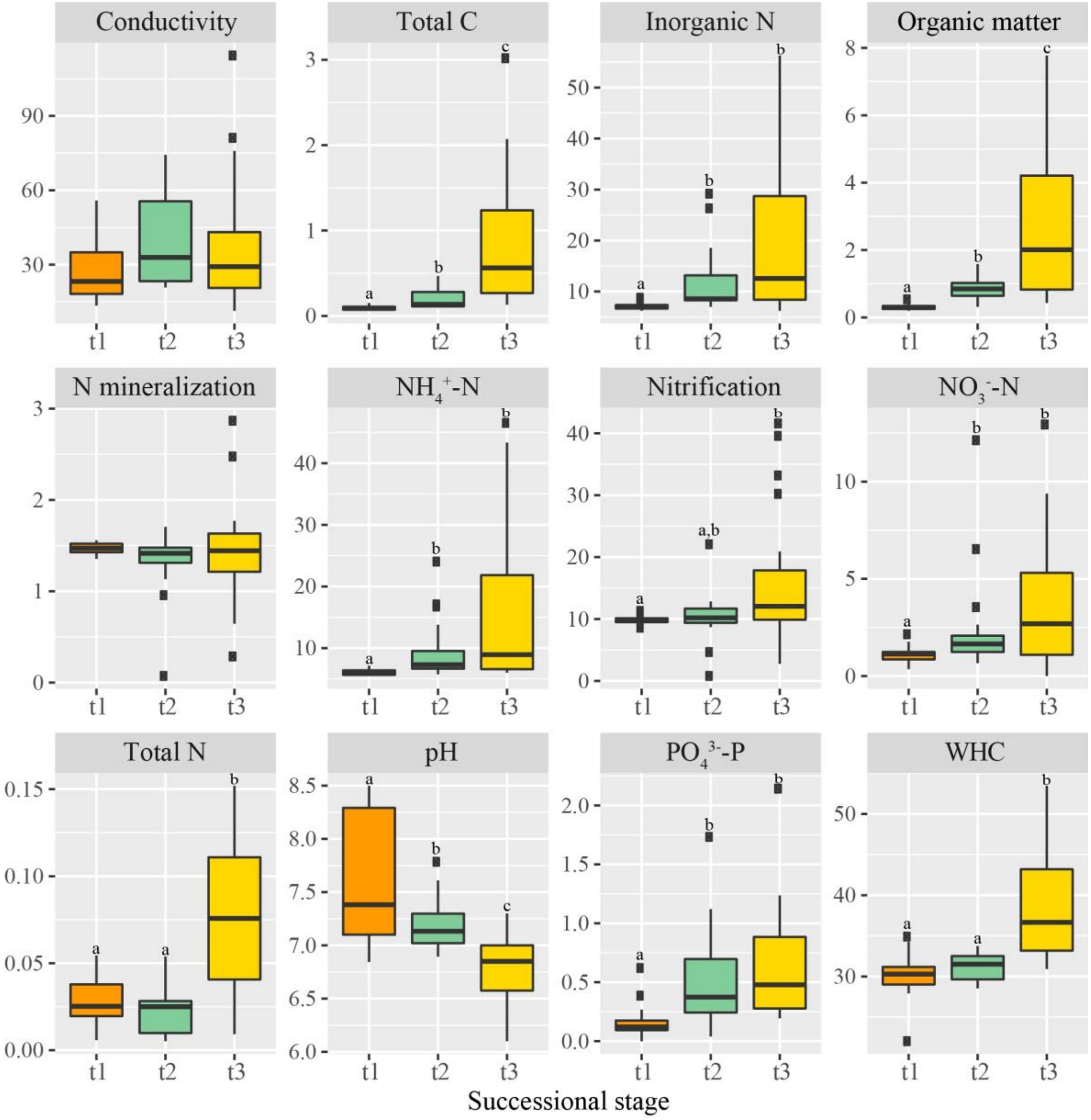

Supplementary Figure S15. Boxplots depicting basic summary data for the 12 soil variables analysed in the present study. Different letters indicate significant differences between successional stages ($P < 0.05$). Variables PO_4^{3-}-P, N mineralization, Nitrification were transformed first to avoid negative values in the data matrix. Supplementary Table S1 contains original and transformed data.
