# Supplementary material for: Differential Colonization and Succession of Microbial Communities in Rock and Soil Substrates on a Maritime Antarctic Glacier Forefield
Source: Front Microbiol. 2020 Feb 7;11:126. doi: 10.3389/fmicb.2020.00126 (PMC7018881; doi:10.3389/fmicb.2020.00126)
Supplement: Supplementary file 17 [file Table_1.DOCX]

**Supplementary Table S1.** Summary information for the obtained sequence data from three organism groups (bacteria, fungi and algae) as well as basic descriptive statistics for the processed sequences under 97%-OTU clustering and DADA2 inferred ASVs.

| Organism group and marker | Samples (rock/soil) | Illumina raw reads | Filtered sequences | Average sequence coverage per sample (rock/soil) | Filtered sequences | Average sequence coverage per sample (rock/soil) |
| --- | --- | --- | --- | --- | --- | --- |
|  |  |  | OTUs | | ASVs | |
| Bacteria (V1-V2) | 120 (60/60) | 12,455,077 | 7,342,953 | 62,404/59,977 | 6,326,580 | 53,392/52,050 |
| Fungi (*ITS1*) | 114 (54/60) | 12,831,030 | 7,385,817 | 79,577/64,488 | 7,053,718 | 80,709/62,100 |
| Algae (*rbcL*) | 88 (36/52) | 9,156,510 | 5,465,524 | 69,498/56,991 | 6,002,455 | 77,510/61,770 |
