# Supplementary material for: Differential Colonization and Succession of Microbial Communities in Rock and Soil Substrates on a Maritime Antarctic Glacier Forefield
Source: Front Microbiol. 2020 Feb 7;11:126. doi: 10.3389/fmicb.2020.00126 (PMC7018881; doi:10.3389/fmicb.2020.00126)
Supplement: Supplementary file 19 [file Table_3.DOCX]

**Supplementary Table S3.** Original data of 60 soil samples for the 12 abiotic variables considered in the present study. Additional columns for PO_4_^3-^-P, N mineralization, and nitrification variables provide transformed values to avoid negative values in the data matrix which could influence subsequent analyses.

| Successional stage (chronosequence) | Sample no | NH_4_^+^-N  (mg kg dsw-1) | Inorganic-N  (mg kg dsw-1) | NO_3_^-^-N  (mg kg dsw-1 ) | PO_4_^3-^-P  (mg kg dsw-1) | Positive  PO_4_^3-^-P | N mineralization  (mg kg-1 day-1) | Positive N  mineralization |
| --- | --- | --- | --- | --- | --- | --- | --- | --- |
| 1 (< 1 year) | 1 | 5,655264848 | 6,781112155 | 1,125847307 | 0,173283278 | 0,223283278 | -0,071649374 | 1,428350626 |
| 1 (< 1 year) | 2 | 5,866943696 | 7,178813067 | 1,311869371 | 0,056252742 | 0,106252742 | -0,080336907 | 1,419663093 |
| 1 (< 1 year) | 3 | 5,884652089 | 6,790279901 | 0,905627812 | 0,12144988 | 0,17144988 | -0,068246424 | 1,431753576 |
| 1 (< 1 year) | 4 | 5,935828708 | 7,134944379 | 1,199115671 | -0,009489489 | 0,040510511 | -0,015866304 | 1,484133696 |
| 1 (< 1 year) | 5 | 5,899505287 | 7,338186777 | 1,438681491 | 0,069281346 | 0,119281346 | -0,114847335 | 1,385152665 |
| 1 (< 1 year) | 6 | 6,323140958 | 7,357598734 | 1,034457776 | 0,003485512 | 0,053485512 | -0,040926 | 1,459074 |
| 1 (< 1 year) | 7 | 7,143542778 | 8,418354835 | 1,274812057 | 0,044939848 | 0,094939848 | -0,079301318 | 1,420698682 |
| 1 (< 1 year) | 8 | 5,604759161 | 6,758130597 | 1,153371437 | 0,335960413 | 0,385960413 | -0,111623862 | 1,388376138 |
| 1 (< 1 year) | 9 | 5,578296784 | 6,51557832 | 0,937281535 | 0,083359305 | 0,133359305 | 0,020505009 | 1,520505009 |
| 1 (< 1 year) | 10 | 5,657163977 | 6,527462755 | 0,870298778 | 0,134776484 | 0,184776484 | 0,058038001 | 1,558038001 |
| 1 (< 1 year) | 11 | 5,877229739 | 6,233590498 | 0,35636076 | 0,110767188 | 0,160767188 | 0,0193214 | 1,5193214 |
| 1 (< 1 year) | 12 | 5,488008323 | 6,638324512 | 1,150316189 | 0,066682802 | 0,116682802 | 0,043286474 | 1,543286474 |
| 1 (< 1 year) | 13 | 5,799434522 | 6,457187705 | 0,657753183 | 0,041051559 | 0,091051559 | -0,011421625 | 1,488578375 |
| 1 (< 1 year) | 14 | 6,084773101 | 6,901838682 | 0,817065582 | -0,049737157 | 0,000262843 | -0,059541945 | 1,440458055 |
| 1 (< 1 year) | 15 | 5,822575933 | 7,05340553 | 1,230829597 | -0,047850072 | 0,002149928 | 0,048758278 | 1,548758278 |
| 1 (< 1 year) | 16 | 7,022382187 | 7,536784576 | 0,51440239 | 0,060064649 | 0,110064649 | -0,057034837 | 1,442965163 |
| 1 (< 1 year) | 17 | 6,576602578 | 7,150484957 | 0,57388238 | 0,073621648 | 0,123621648 | -0,001495462 | 1,498504538 |
| 1 (< 1 year) | 18 | 6,65023135 | 8,413766057 | 1,763534707 | 0,074307789 | 0,124307789 | -0,146871173 | 1,353128827 |
| 1 (< 1 year) | 19 | 6,660223119 | 8,804604109 | 2,144380991 | 0,569562481 | 0,619562481 | 0,038787123 | 1,538787123 |
| 1 (< 1 year) | 20 | 5,478371208 | 6,676319052 | 1,197947844 | 0,217668312 | 0,267668312 | 0,002484038 | 1,502484038 |
| 2 (ca. 13 years) | 1 | 6,81800941 | 8,466524804 | 1,648515394 | 0,267156717 | 0,317156717 | 0,110019823 | 1,610019823 |
| 2 (ca. 13 years) | 2 | 17,06381472 | 29,18849517 | 12,12468045 | 0,650107123 | 0,700107123 | -1,428000384 | 0,071999616 |
| 2 (ca. 13 years) | 3 | 10,28147993 | 12,31264 | 2,031160075 | 0,082899483 | 0,132899483 | -0,053001944 | 1,446998056 |
| 2 (ca. 13 years) | 4 | 13,78710605 | 17,32873332 | 3,541627265 | 0,406238733 | 0,456238733 | -0,328619715 | 1,171380285 |
| 2 (ca. 13 years) | 5 | 5,726229374 | 8,371589762 | 2,645360388 | 0,44193246 | 0,49193246 | 0,205203882 | 1,705203882 |
| 2 (ca. 13 years) | 6 | 7,406657451 | 8,561414515 | 1,154757064 | 0,39429371 | 0,44429371 | -0,034507146 | 1,465492854 |
| 2 (ca. 13 years) | 7 | 9,277281316 | 15,78766252 | 6,5103812 | 0,941471079 | 0,991471079 | -0,544695584 | 0,955304416 |
| 2 (ca. 13 years) | 8 | 7,868827173 | 8,533893963 | 0,66506679 | 0,199150125 | 0,249150125 | -0,114462812 | 1,385537188 |
| 2 (ca. 13 years) | 9 | 7,231249499 | 8,88305311 | 1,651803611 | 0,232484919 | 0,282484919 | -0,145755025 | 1,354244975 |
| 2 (ca. 13 years) | 10 | 7,227560118 | 8,040177359 | 0,81261724 | -0,010493242 | 0,039506758 | -0,054793924 | 1,445206076 |
| 2 (ca. 13 years) | 11 | 16,72528768 | 18,56566126 | 1,840373581 | 1,070247693 | 1,120247693 | -0,36734093 | 1,13265907 |
| 2 (ca. 13 years) | 12 | 6,17877527 | 7,473270737 | 1,294495467 | 0,703521049 | 0,753521049 | -0,041354717 | 1,458645283 |
| 2 (ca. 13 years) | 13 | 8,577813095 | 10,36606694 | 1,788253849 | 0,174443726 | 0,224443726 | -0,175513072 | 1,324486928 |
| 2 (ca. 13 years) | 14 | 6,070899795 | 7,342951843 | 1,272052048 | 0,175017223 | 0,225017223 | 0,015725198 | 1,515725198 |
| 2 (ca. 13 years) | 15 | 5,907928158 | 7,002847796 | 1,094919638 | 1,681874069 | 1,731874069 | 0,028974709 | 1,528974709 |
| 2 (ca. 13 years) | 16 | 6,198324681 | 8,127513915 | 1,929189234 | 0,278956097 | 0,328956097 | -0,153187108 | 1,346812892 |
| 2 (ca. 13 years) | 17 | 6,937558189 | 8,339735347 | 1,402177158 | 0,282537125 | 0,332537125 | -0,05596952 | 1,44403048 |
| 2 (ca. 13 years) | 18 | 7,054388829 | 8,618687466 | 1,564298638 | 0,058947667 | 0,108947667 | 0,140798173 | 1,640798173 |
| 2 (ca. 13 years) | 19 | 24,06758789 | 26,28504028 | 2,217452389 | 0,645618166 | 0,695618166 | -0,227194937 | 1,272805063 |
| 2 (ca. 13 years) | 20 | 8,523828851 | 9,612618004 | 1,088789154 | 0,365186889 | 0,415186889 | -0,126453353 | 1,373546647 |
| 3 (< 23 years) | 1 | 21,69223929 | 27,0490577 | 5,356818405 | 0,277508647 | 0,327508647 | 0,973720075 | 2,473720075 |
| 3 (< 23 years) | 2 | 10,55634269 | 11,93639024 | 1,380047549 | 0,855897708 | 0,905897708 | -0,319581381 | 1,180418619 |
| 3 (< 23 years) | 3 | 17,18296475 | 21,98803512 | 4,805070372 | 0,726931335 | 0,776931335 | 0,125930878 | 1,625930878 |
| 3 (< 23 years) | 4 | 22,24670737 | 35,38105728 | 13,13434991 | 0,76322306 | 0,81322306 | 1,367623879 | 2,867623879 |
| 3 (< 23 years) | 5 | 7,052501894 | 9,795046 | 2,742544105 | 0,421629823 | 0,471629823 | 0,12982206 | 1,62982206 |
| 3 (< 23 years) | 6 | 9,328151059 | 16,0836158 | 6,755464744 | 0,25548371 | 0,30548371 | 0,269682516 | 1,769682516 |
| 3 (< 23 years) | 7 | 6,618413102 | 7,729283465 | 1,110870363 | 0,823923356 | 0,873923356 | 0,139116117 | 1,639116117 |
| 3 (< 23 years) | 8 | 8,531388885 | 8,663869978 | 0,132481093 | 0,436240109 | 0,486240109 | -0,050229749 | 1,449770251 |
| 3 (< 23 years) | 9 | 6,485682983 | 8,383926574 | 1,89824359 | 0,214654186 | 0,264654186 | 0,087340547 | 1,587340547 |
| 3 (< 23 years) | 10 | 6,222359138 | 6,227956777 | 0,005597639 | 0,231924136 | 0,281924136 | -0,010118085 | 1,489881915 |
| 3 (< 23 years) | 11 | 6,356264823 | 7,40068237 | 1,044417548 | 0,16831648 | 0,21831648 | -0,063103443 | 1,436896557 |
| 3 (< 23 years) | 12 | 6,40857307 | 13,99955728 | 7,590984208 | 0,196778806 | 0,246778806 | -0,625038438 | 0,874961562 |
| 3 (< 23 years) | 13 | 5,995099054 | 8,631120041 | 2,636020986 | 0,144379876 | 0,194379876 | -0,208736419 | 1,291263581 |
| 3 (< 23 years) | 14 | 7,331069844 | 8,367324964 | 1,03625512 | 0,236132191 | 0,286132191 | -0,09232734 | 1,40767266 |
| 3 (< 23 years) | 15 | 7,384229211 | 8,249546983 | 0,865317773 | 0,209495761 | 0,259495761 | -0,133257132 | 1,366742868 |
| 3 (< 23 years) | 16 | 28,36372233 | 33,65695644 | 5,293234114 | 0,827371263 | 0,877371263 | -1,215381297 | 0,284618703 |
| 3 (< 23 years) | 17 | 46,90807002 | 56,28682067 | 9,378750644 | 1,186723772 | 1,236723772 | -0,444216878 | 1,055783122 |
| 3 (< 23 years) | 18 | 11,55584379 | 13,18573898 | 1,629895183 | 1,120459791 | 1,170459791 | 0,161600887 | 1,661600887 |
| 3 (< 23 years) | 19 | 39,41705872 | 42,65268639 | 3,235627677 | 0,971612476 | 1,021612476 | -0,276208531 | 1,223791469 |
| 3 (< 23 years) | 20 | 43,36427996 | 48,60107298 | 5,236793022 | 2,116278028 | 2,166278028 | -0,855378897 | 0,644621103 |

(continued)

| Successional stage (chronosequence) | Sample no | Nitrification  (mg kg-1 day-1) | Positive  Nitrification | Organic  matter (%) | Water Holding  Capacity (WHC, %) | pH | Conductivity | Total N  (g/100g) | Total C  (g/100g) |
| --- | --- | --- | --- | --- | --- | --- | --- | --- | --- |
| 1 (< 1 year) | 1 | -1,038374915 | 8,961625085 | 0,325352087 | 31,06159895 | 7,421 | 19,9 | 0,05452 | 0,097 |
| 1 (< 1 year) | 2 | -1,088868153 | 8,911131847 | 0,42916655 | 31,06722015 | 7,597 | 27 | 0,0198 | 0,096 |
| 1 (< 1 year) | 3 | -0,754580499 | 9,245419501 | 0,230473752 | 30,61540028 | 7,59 | 25 | 0,03229 | 0,075 |
| 1 (< 1 year) | 4 | 0,035188244 | 10,03518824 | 0,242742397 | 31,09353317 | 7,341 | 21,5 | 0,02314 | 0,073 |
| 1 (< 1 year) | 5 | -1,142962066 | 8,857037934 | 0,531558119 | 27,91763593 | 8,358 | 50,9 | 0,03789 | 0,117 |
| 1 (< 1 year) | 6 | 1,063643505 | 11,06364351 | 0,299136244 | 27,98667064 | 8,498 | 55,9 | 0,02218 | 0,151 |
| 1 (< 1 year) | 7 | 1,264432833 | 11,26443283 | 0,323964497 | 34,25821983 | 6,844 | 14,9 | 0,02741 | 0,061 |
| 1 (< 1 year) | 8 | -0,325540647 | 9,674459353 | 0,270286151 | 31,48000634 | 8,371 | 33,8 | 0,01824 | 0,102 |
| 1 (< 1 year) | 9 | 0,385754464 | 10,38575446 | 0,197363227 | 29,11634301 | 8,288 | 44,1 | 0,02079 | 0,148 |
| 1 (< 1 year) | 10 | 0,184347809 | 10,18434781 | 0,327772326 | 28,5051114 | 8,452 | 38,6 | 0,0216 | 0,106 |
| 1 (< 1 year) | 11 | -0,189849271 | 9,810150729 | 0,237501343 | 30,89113306 | 7,47 | 18,4 | 0,03785 | 0,08 |
| 1 (< 1 year) | 12 | 0,007974795 | 10,0079748 | 0,251732944 | 29,7702933 | 7,2 | 18,8 | 0,04329 | 0,077 |
| 1 (< 1 year) | 13 | -0,178254114 | 9,821745886 | 0,245367628 | 29,11375517 | 7,1 | 14,7 | 0,03666 | 0,074 |
| 1 (< 1 year) | 14 | -0,400614408 | 9,599385592 | 0,252058478 | 29,68040605 | 7,2 | 13,5 | 0,04603 | 0,072 |
| 1 (< 1 year) | 15 | 0,091514981 | 10,09151498 | 0,310735611 | 32,12840824 | 7 | 16 | 0,019175 | 0,0615 |
| 1 (< 1 year) | 16 | 0,023683108 | 10,02368311 | 0,284799491 | 31,65682488 | 7,1 | 20 | 0,02877 | 0,068 |
| 1 (< 1 year) | 17 | -0,140203094 | 9,859796906 | 0,277834461 | 29,98732453 | 7,2 | 17,6 | 0,01199 | 0,082 |
| 1 (< 1 year) | 18 | -1,4520151 | 8,5479849 | 0,375707119 | 28,75008608 | 7 | 25,6 | 0,01136 | 0,095 |
| 1 (< 1 year) | 19 | 0,778225051 | 10,77822505 | 0,412412015 | 34,90372383 | 7,1 | 26,4 | 0,03824 | 0,115 |
| 1 (< 1 year) | 20 | -0,057224073 | 9,942775927 | 0,385865836 | 22,04935747 | 8,3 | 53,7 | 0,0058 | 0,133 |
| 2 (ca. 13 years) | 1 | 0,200745399 | 10,2007454 | 1,155108478 | 32,22479471 | 7,511 | 57,6 | 0,03952 | 0,322 |
| 2 (ca. 13 years) | 2 | -9,18629129 | 0,81370871 | 1,089604082 | 32,79085289 | 6,923 | 72,7 | 0,02431 | 0,338 |
| 2 (ca. 13 years) | 3 | 2,837624497 | 12,8376245 | 0,732655317 | 29,07263902 | 7,14 | 54,9 | 0,02212 | 0,113 |
| 2 (ca. 13 years) | 4 | 2,72568884 | 12,72568884 | 1,412584847 | 30,59923138 | 7,22 | 64,5 | 0,03595 | 0,396 |
| 2 (ca. 13 years) | 5 | 1,996449466 | 11,99644947 | 0,847569858 | 29,61829399 | 7,61 | 44,6 | 0,0263 | 0,117 |
| 2 (ca. 13 years) | 6 | -0,666867092 | 9,333132908 | 0,562400821 | 31,47732339 | 7,064 | 20,8 | 0,03016 | 0,103 |
| 2 (ca. 13 years) | 7 | -5,308109707 | 4,691890293 | 0,665253308 | 32,51428668 | 6,962 | 33,8 | 0,02678 | 0,145 |
| 2 (ca. 13 years) | 8 | -0,11678842 | 9,88321158 | 0,696406097 | 32,70375644 | 6,998 | 31,9 | 0,00524 | 0,144 |
| 2 (ca. 13 years) | 9 | -1,080516311 | 8,919483689 | 0,505407864 | 31,57185836 | 6,893 | 27,9 | 0,00524 | 0,144 |
| 2 (ca. 13 years) | 10 | 0,369032139 | 10,36903214 | 0,517888986 | 33,00892643 | 6,994 | 37,5 | 0,00965 | 0,099 |
| 2 (ca. 13 years) | 11 | 1,604670331 | 11,60467033 | 1,555102977 | 31,79964393 | 7,064 | 74,3 | 0,00931 | 0,469 |
| 2 (ca. 13 years) | 12 | -0,574653004 | 9,425346996 | 1,584983616 | 32,06591565 | 7,782 | 63,8 | 0,05394 | 0,417 |
| 2 (ca. 13 years) | 13 | -0,172961808 | 9,827038192 | 0,599144574 | 32,5511638 | 7,255 | 34,1 | 0,02573 | 0,13 |
| 2 (ca. 13 years) | 14 | 0,465457368 | 10,46545737 | 1,000508071 | 28,54161581 | 7,385 | 23,2 | 0,00812 | 0,11 |
| 2 (ca. 13 years) | 15 | 0,465194029 | 10,46519403 | 0,847359704 | 28,88486749 | 7,474 | 23,9 | 0,02741 | 0,119 |
| 2 (ca. 13 years) | 16 | -1,293749482 | 8,706250518 | 0,314504124 | 29,65819753 | 7,268 | 22 | 0,01 | 0,102 |
| 2 (ca. 13 years) | 17 | 0,211140537 | 10,21114054 | 0,862262186 | 29,39122984 | 7,027 | 22,3 | 0,01331 | 0,115 |
| 2 (ca. 13 years) | 18 | 1,969349101 | 11,9693491 | 0,852776936 | 29,87648182 | 7,132 | 23,4 | 0,02275 | 0,099 |
| 2 (ca. 13 years) | 19 | 12,0236762 | 22,0236762 | 0,861492849 | 33,7516307 | 7,132 | 32 | 0,03913 | 0,266 |
| 2 (ca. 13 years) | 20 | -0,052910527 | 9,947089473 | 0,657501494 | 31,12275636 | 7,064 | 21,3 | 0,02768 | 0,1575 |
| 3 (< 23 years) | 1 | 20,21165469 | 30,21165469 | 6,238710574 | 40,50987137 | 6,5 | 34,4 | 0,107 | 1,2 |
| 3 (< 23 years) | 2 | -0,330327717 | 9,669672283 | 6,190466088 | 53,45494202 | 6,3 | 40,5 | 0,15193 | 3,05 |
| 3 (< 23 years) | 3 | 10,90960871 | 20,90960871 | 2,920250824 | 43,03290917 | 6,2 | 27,7 | 0,13247 | 1,35 |
| 3 (< 23 years) | 4 | 32,03454612 | 42,03454612 | 2,32389252 | 35,62102756 | 6,1 | 46,6 | 0,07576 | 0,575 |
| 3 (< 23 years) | 5 | 3,095212518 | 13,09521252 | 0,600700595 | 33,00385793 | 6,7 | 14,3 | 0,00924 | 0,18 |
| 3 (< 23 years) | 6 | 6,851305362 | 16,85130536 | 0,73469546 | 31,57461501 | 6,4 | 21,4 | 0,04182 | 0,182 |
| 3 (< 23 years) | 7 | 2,656066504 | 12,6560665 | 0,431705329 | 33,23950838 | 6,9 | 11,4 | 0,04896 | 0,132 |
| 3 (< 23 years) | 8 | 0,61870667 | 10,61870667 | 1,693461692 | 37,71012508 | 6,7 | 18,3 | 0,07575 | 0,552 |
| 3 (< 23 years) | 9 | 1,478224738 | 11,47822474 | 0,586878213 | 35,27876157 | 6,9 | 11,6 | 0,03239 | 0,179 |
| 3 (< 23 years) | 10 | 0,742523489 | 10,74252349 | 0,678633975 | 35,50801186 | 6,8 | 15,5 | 0,03796 | 0,245 |
| 3 (< 23 years) | 11 | -0,234884262 | 9,765115738 | 0,859613091 | 30,92280461 | 7 | 24,3 | 0,02171 | 0,275 |
| 3 (< 23 years) | 12 | -7,218772442 | 2,781227558 | 1,376113826 | 32,87084584 | 7 | 21,8 | 0,0395 | 0,367 |
| 3 (< 23 years) | 13 | -2,253885711 | 7,746114289 | 1,286984063 | 32,1852154 | 7 | 30,7 | 0,04109 | 0,384 |
| 3 (< 23 years) | 14 | 0,125401258 | 10,12540126 | 3,849103534 | 38,4975875 | 6,6 | 22 | 0,1013 | 0,712 |
| 3 (< 23 years) | 15 | -0,063458832 | 9,936541168 | 1,23320782 | 34,26659045 | 7,3 | 41,2 | 0,05696 | 0,443 |
| 3 (< 23 years) | 16 | 5,070908518 | 15,07090852 | 5,294446631 | 43,72913217 | 6,8 | 72,2 | 0,1322 | 1,44 |
| 3 (< 23 years) | 17 | 29,56027463 | 39,56027463 | 7,775567392 | 45,43645095 | 6,9 | 81,1 | 0,13851 | 2,07 |
| 3 (< 23 years) | 18 | 3,750879907 | 13,75087991 | 6,670221843 | 41,99617175 | 7,1 | 42 | 0,12264 | 1,49 |
| 3 (< 23 years) | 19 | 23,18290105 | 33,18290105 | 2,706092428 | 43,67922482 | 7,2 | 75,9 | 0,10059 | 1,09 |
| 3 (< 23 years) | 20 | -3,173036525 | 6,826963475 | 3,597545435 | 44,299646 | 7,1 | 114,4 | 0,10407 | 1,09 |
