# Supplementary material for: Differential Colonization and Succession of Microbial Communities in Rock and Soil Substrates on a Maritime Antarctic Glacier Forefield
Source: Front Microbiol. 2020 Feb 7;11:126. doi: 10.3389/fmicb.2020.00126 (PMC7018881; doi:10.3389/fmicb.2020.00126)
Supplement: Supplementary file 20 [file Table_4.DOCX]

**Supplementary Table S4.** Mantel statistics based on Spearman’s rank correlation *rho* using Bray-Curtis dissimilarity matrices of OTU data and environmental variables.

| Organismal group | Mantel statistic *r* | Significance |
| --- | --- | --- |
| Bacteria | 0.3385 | 0.001 |
| Fungi | 0.3517 | 0.001 |
| Algae | 0.2571 | 0.001 |
