# Supplementary material for: Differential Colonization and Succession of Microbial Communities in Rock and Soil Substrates on a Maritime Antarctic Glacier Forefield
Source: Front Microbiol. 2020 Feb 7;11:126. doi: 10.3389/fmicb.2020.00126 (PMC7018881; doi:10.3389/fmicb.2020.00126)
Supplement: Supplementary file 21 [file Table_5.DOCX]

**Supplementary Table S5.** The three subsets of environmental variables with the highest correlation to community data for each organism group (bacteria, fungi and algae) based on BIOENV analyses. The best subset is highlighted in bold.

| Organismal group | Subset of environmental variables | Correlation coefficient (Spearman) |
| --- | --- | --- |
| Bacteria | **Organic matter + pH** | **0.4863** |
|  | Organic matter + pH + total N | 0.4707 |
|  | Organic matter + pH + total N + Conductivity | 0.4259 |
| Fungi | **Organic matter + pH** | **0.4481** |
|  | Organic matter | 0.4055 |
|  | Organic matter + pH + Conductivity | 0.3694 |
| Algae | **Organic matter** | **0.3026** |
|  | Organic matter + pH | 0.2569 |
|  | Organic matter + pH + Conductivity | 0.2312 |
