# Supplementary material for: Differential Colonization and Succession of Microbial Communities in Rock and Soil Substrates on a Maritime Antarctic Glacier Forefield
Source: Front Microbiol. 2020 Feb 7;11:126. doi: 10.3389/fmicb.2020.00126 (PMC7018881; doi:10.3389/fmicb.2020.00126)
Supplement: Supplementary file 22 [file Table_6.DOCX]

**Supplementary Table S6.** Results of ANOVA tests implemented to assess the significance of the individual axes and environmental variables after conducting distance based redundancy analyses (db-RDAs), using selected edaphic variables that explained most of the variability in soil communities of bacteria, fungi and algae.

| Organismal group |  |  | Df | Sum of Sqs. | F | Pr (> F) |
| --- | --- | --- | --- | --- | --- | --- |
| Bacteria | Axes | *dbRDA1* | 1 | 2.0945 | 8.1714 | 0.001 |
|  |  | *dbRDA2* | 1 | 0.4452 | 1.7370 | 0.010 |
|  |  | Residual | 57 | 14.6105 |  |  |
|  | Environmental  variables | *pH* | 1 | 1.3842 | 5.4003 | 0.002 |
|  |  | *Org. matter* | 1 | 1.1555 | 4.5081 | 0.002 |
|  |  | Residual | 57 | 14.6105 |  |  |
| Fungi | Axes | *dbRDA1* | 1 | 2.0164 | 6.5683 | 0.001 |
|  |  | *dbRDA2* | 1 | 0.4206 | 1.3702 | 0.022 |
|  |  | Residual | 56 | 17.1912 |  |  |
|  | Environmental  variables | *pH* | 1 | 1.2385 | 4.0344 | 0.002 |
|  |  | *Org. matter* | 1 | 1.1985 | 3.9041 | 0.002 |
|  |  | Residual | 56 |  |  |  |
| Algae | Axes | *dbRDA1* | 1 | 1.4048 | 5.7932 | 0.001 |
|  |  | *dbRDA2* | 1 | 0.3302 | 1.3616 | 0.030 |
|  |  | Residual | 49 | 11.8824 |  |  |
|  | Environmental  variables | *Org. matter* | 1 | 1.3885 | 5.7259 | 0.020 |
|  |  | *pH* | 1 | 0.3465 | 1.4289 | 0.050 |
|  |  | Residual | 49 |  |  |  |
